# The Influence of 5′,8-Cyclo-2′-deoxypurines on the Mitochondrial Repair of Clustered DNA Damage in Xrs5 Cells: The Preliminary Study

**DOI:** 10.3390/molecules26227042

**Published:** 2021-11-22

**Authors:** Karolina Boguszewska, Julia Kaźmierczak-Barańska, Bolesław T. Karwowski

**Affiliations:** DNA Damage Laboratory of Food Science Department, Faculty of Pharmacy, Medical University of Lodz, ul. Muszynskiego 1, 90-151 Lodz, Poland; karolina.boguszewska@umed.lodz.pl (K.B.); julia.kazmierczak-baranska@umed.lodz.pl (J.K.-B.)

**Keywords:** 5′,8-cyclo-2′-deoxyadenosine (cdA), 5′,8-cyclo-2′-deoxyguanosine (cdG), clustered DNA damage, DNA repair, base excision repair, mtDNA

## Abstract

The 5′,8-cyclo-2′-deoxypurines (cdPus) affect the DNA structure. When these bulky structures are a part of clustered DNA lesions (CDL), they affect the repair of the other lesions within the cluster. Mitochondria are crucial for cell survival and have their own genome, hence, are highly interesting in the context of CDL repair. However, no studies are exploring this topic. Here, the initial stages of mitochondrial base excision repair (mtBER) were considered—the strand incision and elongation. The repair of a single lesion (apurinic site (AP site)) accompanying the cdPu within the double-stranded CDL has been investigated for the first time. The type of cdPu, its diastereomeric form, and the interlesion distance were taken into consideration. For these studies, the established experimental model of short oligonucleotides (containing AP sites located ≤7 base pairs to the cdPu in both directions) and mitochondrial extracts of the xrs5 cells were used. The obtained results have shown that the presence of cdPus influenced the processing of an AP site within the CDL. Levels of strand incision and elongation were higher for oligos containing RcdA and ScdG than for those with ScdA and RcdG. Investigated stages of mtBER were more efficient for DNA containing AP sites located on 5′-end side of cdPu than on its 3′-end side. In conclusion, the presence of cdPus in mtDNA structure may affect mtBER (processing the second mutagenic lesion within the CDL). As impaired repair processes may lead to serious biological consequences, further studies concerning the mitochondrial repair of CDL are highly demanded.

## 1. Introduction

Mitochondria are unique organelles of bacterial origin that produce energy for the cell through oxidative phosphorylation (OXPHOS) [1]. Mitochondria have their own DNA (mtDNA), which encodes proteins crucial for energy production [2]. One human cell contains from a few up to a few thousands of mtDNA molecules. Mitochondrial genes are often damaged to some extent, and mutations within mtDNA genes often need to occur within at least 60% of mtDNA copies for phenotypic manifestation. A major part of the mitochondrial proteome is encoded by nuclear DNA, translated into the cytosol, and transported into mitochondria [3]. Protein translocation is a process highly susceptible to errors. Its dysfunctions may lead to decreased protein number due to their wrong localization or premature degradation outside the mitochondria, which may lead to energy-production impairment and subsequent cell death. Also, the byproducts of OXPHOS (e.g., mitochondrial reactive oxygen species (mtROS)) can cause mtDNA damage [1]. Oxidative stress may inhibit the ribosome functioning in cytosol affecting the overall protein synthesis [4]. Some neurogenerative, metabolic, and age-related diseases, and cancers are suspected to result at least in part from mutations in mitochondrial and/or nuclear genes encoding mitochondrial proteins [5]. Diseases of mitochondrial origin affect approximately 1 in every 5000 people and are still a therapeutic challenge [5]. They impair the most energy-dependent tissues such as the heart, muscles, brain, and eyes, which manifests as neuropathies, myopathies, blindness, hearing loss, kidney, or liver diseases [5]. Aging and age-related diseases also correlate with increasing mtDNA damage [6]. What is even more interesting, the overall condition of mitochondria may indicate the active SARS-CoV-2 infection [7]. The recent review stated that aging-like degradation and loss of mitochondrial function have been observed in COVID-19 patients. Furthermore, during COVID-19 infection increased number of damaged mitochondria accumulated in the cell and led to a higher inflammation rate [7]. Approximately 30% of mitochondrial proteins are present in both, mitochondria, and cytoplasm, and/or nucleus. Many questions concerning the functions of mitochondrial proteins remain unanswered due to difficulties with isolating specific proteins from this organelle. However, some novel solutions for mitochondrial protein detection are considered e.g., the split-GFP system which gives fluorescent signal only after proteins of interest enter the mitochondria [8].

MtDNA is particularly vulnerable to ^●^OH activity, which may induce the formation of the 5′,8-cyclo-2′-deoxypurines (cdPus). The formation of the additional covalent bond between C5′ and C8 results from one of the hydrogen atom abstraction by ^●^OH from the C5′ group. Approximately 10^2^–10^5^ DNA lesions are formed daily in every human cell [9]. Clustered DNA lesions (CDL) are observed as 2 or more individual lesions occurring within 1–2 helical turns of DNA and may contain a different damage type [10]. CdPus show different biological impact depending on the C5′chirality (5′*S* or 5′*R* diastereomer) [11]. CdPus represent a tandem DNA lesion type within one nucleotide that increases the rigidity of DNA structure [10,12]. The previous studies have shown the geometry of the DNA helix is changed 5′ from the lesion [13,14]. CdPus are removed from DNA via nucleotide excision repair (NER) which does not take place in mitochondria [11]. Moreover, cdPus interfere with replication and/or transcription [10,15,16]. Most cdPus studies focus on the nuclear repair pathways [17,18,19,20]. The results show that the cellular repair capacity is altered depending on the type of cdPu (namely, 5′,8-cyclo-2′-deoxyadenosine (cdA) or 5′,8-cyclo-2′-deoxyguanosine (cdG)), their diastereomeric form (5′*S* or 5′*R*), and the distance between lesions within a cluster. Moreover, these characteristics directly influenced the activity of BER enzymes towards the other lesion within the CDL [10,21]. Due to the high levels of cdPus detected in human cells (cdA: 0.01 (5′*R*) and 0.1 (5′*S*) lesions per 10^6^ DNA nucleosides; cdG: 2 (5′*R*) and 10 (5′*S*) lesions per 10^6^ DNA nucleosides) and their different biological consequences (e.g., mutations, replication blocks, and impaired repair pathways), the impact of cdPus on the mitochondrial BER (mtBER) should be explored [11]. To our knowledge, currently, no study showed the levels of cdPus in the mitochondrial genome.

The BER is the most evolutionarily conserved repair system and the main one in mammalian mitochondria. It may correct a single base (short-patch BER, SP-BER) or a fragment of 2–12 nucleotides (long-patch BER, LP-BER) [22]. It is a multistep process consisting of damage recognition, lesion excision (AP site formation), subsequent strand incision (gap formation), undamaged nucleotide incorporation (gap-filling), and strand rejoining [23]. Other repair systems detected in mitochondria include mismatch repair (MMR), single-strand break repair (SSBR), microhomology-mediated end joining (MMEJ), and homologous recombination (HR) [24,25]. The BER process is impaired when repair enzymes cannot form complexes with DNA due to structural changes in the double helix, such as those resulting from cdPus occurrence [10]. Interestingly, a recent study showed that BER can remove ScdA from the genome, but the lesion location was crucial for proper enzymes’ action [26]. Even though the mitochondrial BER (mtBER) is the best-characterized repair system in mitochondria, many questions remain to be answered.

**Figure 1:**
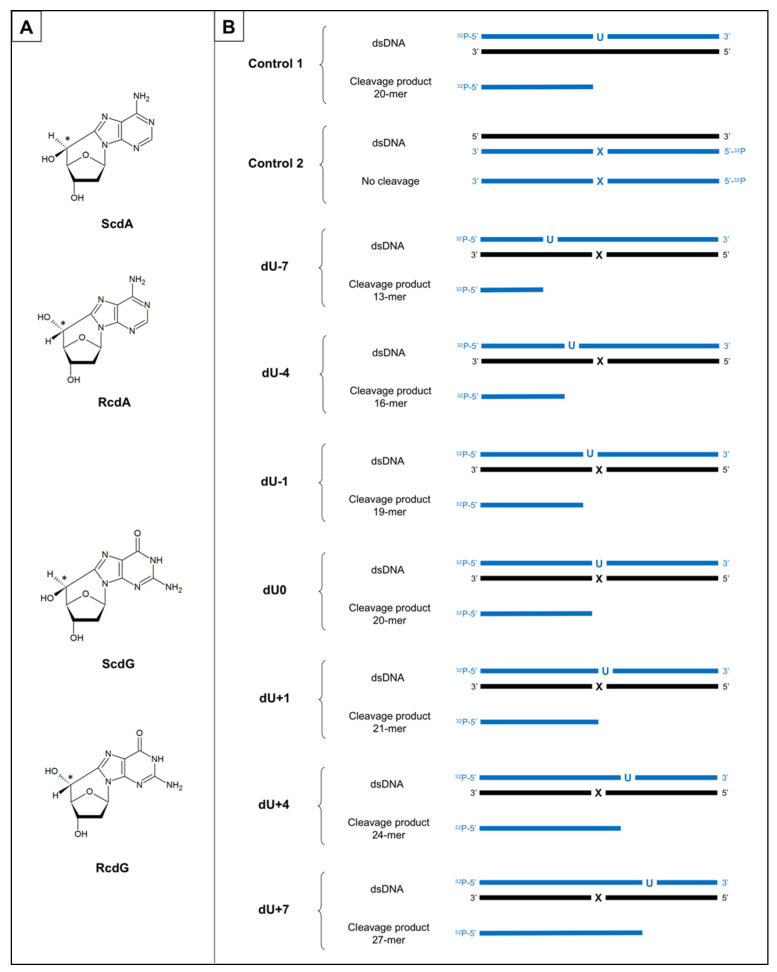
(**A**) The representation of chemical structures of the 5′,8-cyclo-2′-deoxypurines (cdPus): (5′*S*)-5′,8-cyclo-2′-deoxyadenosine (ScdA), (5′*R*)-5′,8-cyclo-2′-deoxyadenosine (RcdA), (5′*S*)-5′,8-cyclo-2′-deoxyguanosine (ScdG), (5′*R*)-5′,8-cyclo-2′-deoxyguanosine (RcdG); (**B**) The schematic presentation of investigated ds-oligonucleotides (40 bp) with labeled ends, lesion positioning and cleavage products. U—represents location of an AP site obtained from 2′-deoxyuridine (dU) after treatment with uracil DNA-glycosylase (UDG); X—represents location of the cdPu; ^32^P—represents strand end labeled with [γ-^32^P]ATP; blue color—represents radiolabeled strands of dsDNA and corresponding cleavage products subsequently observed on radiograms; negative numbers—dsDNA with clustered lesions on two strands where AP site is located 1–7 base pairs in 3′ direction; positive numbers—dsDNA with clustered lesions on two strands where AP site is located 1–7 base pairs in 5′ direction.

This work investigated the two initial steps of the mtBER pathway (strand incision and strand elongation) processing the single AP site lesion which was accompanied by the cdPu within the bi-stranded cluster (Figure 1).

The obtained results have shown that the relative distance between a single lesion (AP site) on one strand and cdPu on the other strand influences the repairability of the first one. In this study, the bi-stranded CDL (Figure 1) were tested in mitochondrial extracts (ME) of xrs5 cells (X-ray sensitive Chinese hamster ovarian mutant cell line), which is an established model for CDL studies. Moreover, the impact of different cyclopurines and their isomers was investigated. To our knowledge, this is the first demonstration of the influence of both 5′*S* and 5′*R* cdPus on the repair of accompanying lesions located within CDL in mitochondria (probably due to difficulties with the chemical synthesis of RcdG). As the cdPus have a diagnostic potential [27,28,29], knowing their implications in mitochondria may lead to new applications in medicine and/or new pharmaceuticals development.

## 2. Results and Discussion

The formation of cdPus results from the hydroxyl radical action upon human genetic material; hence, it is important to determine their impact on molecular processes. The presented work examined whether the type, diastereomeric form of cdPu, and the distance between cdPu and AP site (1–7 base pairs in 3′-end and 5′-end direction) within CDL impact the first two stages of mitochondrial BER pathway—the strand incision (gap formation) resulting from total mitochondrial endonucleolytic activity and strand elongation (incorporation of new nucleotides into the gap) catalyzed by polymerases. The established model [10,20] for the experiments employed 40-mer synthetic double-stranded oligonucleotides containing dU (as the AP site precursor) on one strand and cdPu on the complementary strand (Figure 1, full sequences are listed in Table S1). The model cell line of X-ray-sensitive Chinese hamster ovarian mutant cell line (xrs5, Ku80 deficient) was used to obtain mitochondrial extracts (ME). The functional activity test has been performed to evaluate the quality of ME in the context of mitochondrial proteins involved in BER repair and to exclude the possibility of contamination with the nuclear BER proteins (Figure S14).

Here, the total activity of ME proteins involved in the first two stages of the mtBER pathway was studied in the context of AP site repair located within CDL that also contain cdPu. The activity of ME proteins was tested for a control oligonucleotide with a single lesion, as the reference point for future studies (Control 1, Figure 1, Table S1). For Control 1, the percentage of strand cleavage reached 71.92% after 30 min (Table S2). This endonucleolytic activity was approximately 20% lower in comparison with nuclear extracts (NE) studied previously [20]. It can be assumed it is a result of faster nDNA repair when compared to mtDNA. The lesion recognition and removal must be rapid in the nucleus due to the highest importance of maintaining correct genetic information for the whole cell. Moreover, proteins involved in mitochondrial repair may act slower as they are translocated into the organelle upon need, and only some molecules are present on-site before DNA damage [30].

**Figure 2:**
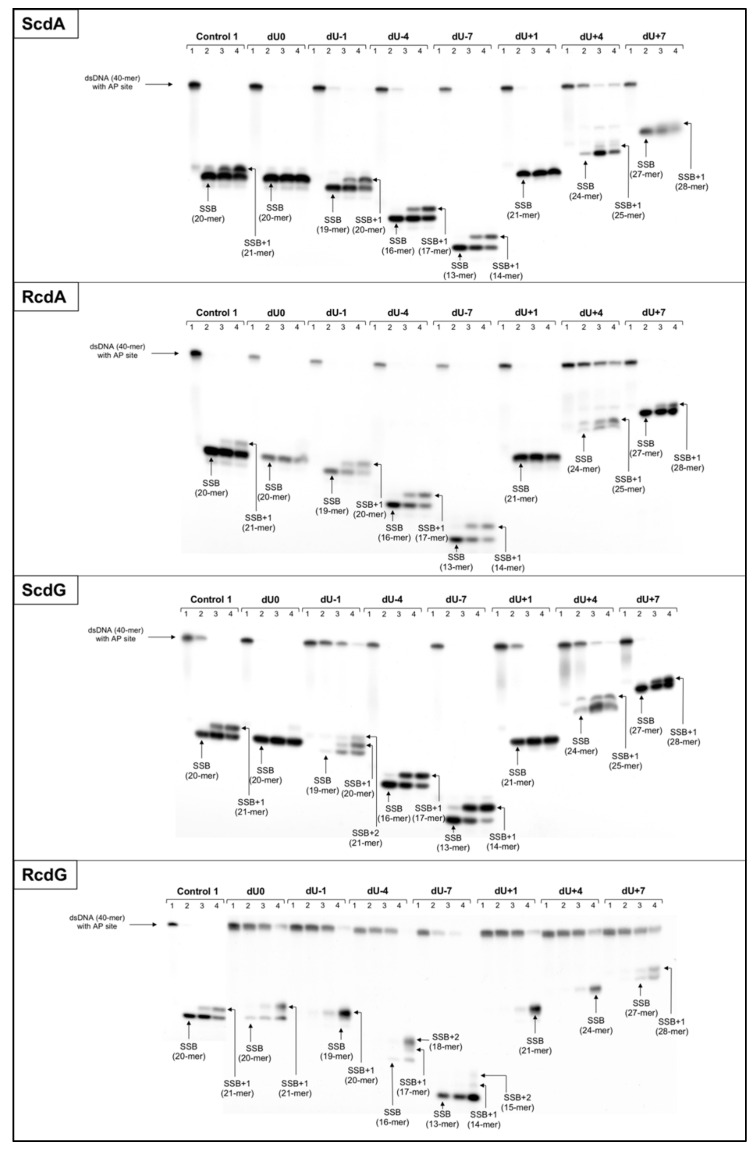
The representative autoradiograms of denaturing PAGE presenting the strand incision and elongation of dsDNA containing clustered damage with AP site on one strand (observed on the gel) and ScdA on the opposing strand (not observed on the gel). Control 1: dsDNA with a single AP site lesion on one strand; dU0: dsDNA with clustered lesions on two strands opposite to each other; negative numbers: dsDNA with clustered lesions on two strands where AP site is located 1–7 base pairs in 3′-end direction; positive numbers: dsDNA with clustered lesions on two strands where AP site is located 1–7 base pairs in 5′-end direction; SSB: dsDNA with single-strand break obtained after AP site incision (gap formation); SSB+1/SSB+2: dsDNA with single-strand break obtained after AP site incision (gap formation) and incorporation of 1 or 2 undamaged nucleotide units. Each lane corresponds to a different assay time: lane 1–0 h; lane 2–0.5 h; lane 3–3 h; lane 4–6 h. Each experiment was performed three times for consistency (individual replications and graphical representation of results with SD are available in Supplementary Materials).

Polymerase activity assay was also performed for the control oligonucleotide (Control 1, Figure 1, Table S1). Incorporation of new nucleotide units (NU) was observed as an elongated strand (e.g., SSB+1 indicated incorporation of 1NU to the DNA previously cleaved) (Figure 2).

The polymerase efficiency for Control 1 reached 35.16% after 6 h with strand elongation exclusively by 1NU (Figure 2, Figures S2, S3 and S8, Table S3). For the NE presented in the related study, a similar level of polymerases activity was reached after 30 min [20].

### 2.1. The Influence of 5′,8-Cyclo-2′-deoxypurines on the Endonucleolytic Activity in Mitochondria

The total mitochondrial endonucleolytic activity has been taken under consideration and observed as the strand incision at the location of the AP site creating SSB. Double-stranded oligonucleotides with cdA or cdG (5′*S* and 5′*R* isomers) on one strand and with an AP site on the complementary strand were examined. The strand incision was observed on the radiogram as bands corresponding to shorter oligonucleotide fragments: from 13-mer for −7 position up to 27-mer for +7 position (Figure 1, Figure 2, Figure S2, S3, S8 and S9). Almost all examined strands with AP sites accompanied by cdA on the complementary strand were incised after 30 min of incubation with ME with ~80–92% efficiency (Table S4). The exceptions were observed for ScdA/dU+4 and RcdA/dU+4 where it took 6 h to reach 61.4% and 64.41%, respectively (Figures S4 and S5, Table S4). Interestingly, Control 1 (with a single AP site) showed a lower level of strand cleavage (71.92% after 30 min) than duplexes with bi-stranded CDL. It may indicate that the presence of cdA within CDL enhanced the first step of the mtBER process (AP site incision). The fact that the AP site in the +4 position strongly inhibits the endonucleolytic activity of the ME aligns with previous observations [10,20]. Surprisingly, the strand incision level was up to 10% higher for ds-oligos with RcdA than ScdA (Figure S6). This is contrary to previously studied NE where the presence of ScdA within the duplex increased AP site cleavage level more than RcdA [20]. It allows us to conclude that the presence of ScdA in mtDNA is less beneficial than RcdA for enzymatic AP site scission by apurinic/apyrimidinic endonuclease (APE1) or other enzymes (DNA glycosylases/AP lyases) possessing endonucleolytic activity (e.g., endonuclease VIII-like 1 (NEIL1), 8-oxoguanine glycosylase (OGG1), endonuclease III (NTH)). Substrate ds-oligos were incised with the total efficiency increasing in the following order for RcdA and ScdA, respectively (data compared for 30 min reaction time): +4 < +7 < −1 < −4 ~+1 ~ −7 < 0 and +4 < −1 < +7 ~0 < +1 < −4 < −7.

For ds-oligonucleotides containing AP site and cdG, the trends of strand incision varied depending on the distance between these lesions. For ScdG, the lowest efficiency has been noted for ScdG/dU−1 (16.91% after 30 min) and the highest for ScdG/dU0 (97.51% after 30 min) (Table S6). AP sites located in positions 0, −4, −7, and +7 reached incision levels up to 25% higher than Control 1, while for AP sites in positions −1, +1, and +4 SSB formation was inhibited below Control 1 level (compared for 30 min reaction time, Figure S10, Table S6). These results were in agreement with the past NE studies where AP sites located −1, +1, and +4 from ScdA have also shown lower endonucleolytic activity [20]. Oligonucleotides containing RcdG were incised less efficiently than those containing ScdG (except for dU−7 where 5′*R* showed 3.5% higher efficiency than 5′*S*) (Figure S12). Interestingly, ds-oligonucleotides containing cdA presented inverse trend (5′*R* diastereomers reached higher rates of strand incision than 5′*S*, Figure S6). These observations are in good agreement with previous results for the NE study [20]. The strand incision efficiency increased as follows (data compared for 30 min reaction time): −4 < +1 ~ +4 < +7 < −1 < 0 < −7 (RcdG); −1 < +4 < +1 < −7 ~ +7 < −4 < 0 (ScdG). Noteworthy, in the case of AP site located +4 nucleobases from 5′*S* and 5′*R* cdG, the level of total mitochondrial endonucleolytic efficiency reached only ~55–58% after 6 h (Table S6). These levels of strand cleavage were lower than for corresponding oligos with cdA (61.40% for ScdA/dU+4 and 64.41% for RcdA/dU+4 after 6 h, Table S4) and can be considered as inhibitory for this mtBER stage. Surprisingly, an even stronger inhibitory effect was observed for ScdG/dU−1 (31.66% after 6 h) and RcdG/dU−1 (55.27% after 6 h), which was not observed for cdA for ME nor NE [20]. What is more, AP site hydrolysis took more time in the case of ME (30 min) than NE (1 min) [20]. The majority of mitochondrial proteins involved in DNA repair are translocated into mitochondria when needed (after DNA damage is detected). This study concerned cells in their native state (without external damaging factors applied to the cell culture), which might have resulted in the low quantity of proteins with endonucleolytic activity in the ME. One of the known examples is APE1 that is “stored” and translocated from intermembrane space into mitochondrial matrix when needed; therefore, it may take more time before the enzyme processes the damaged mtDNA, and possibly a lower enzyme quantity is present in mitochondria with undamaged DNA [31]. In mitochondria, the AP sites located in both directions from cdPus (in the 5′-end direction (positive numbers) and the 3′-end direction (negative numbers)) were hydrolyzed with similar efficiency. It was contrary to results concerning NE of the same cell line, where AP sites in the 5′-end direction from cdPus were incised slower [20].

### 2.2. The Influence of 5′,8-Cyclo-2′-deoxypurines on DNA Synthesis in Mitochondria

The influence of cdPu located within the CDL on the gap-filling step of mtBER was also taken under consideration. Polymerases play a crucial role in the replication and repair of genetic material in both, nucleus and mitochondria. Previous reports showed that both cdPus diastereomers have a different impact on the DNA synthesis during genome repair. For example, replication in *Escherichia coli* may be blocked by the 5′*S* isomer of cdG and cdA, but not the 5′*R* isomer [15,16,32,33]. The mitochondrial pool of polymerases differs from those present in the nucleus. Apart from the best characterized mitochondrial replicative polymerase γ (PolG), other types of this enzyme have also been detected in mitochondria (e.g., polymerase β (PolB), PrimPol, Rev3 subunit of polymerase ζ (PolZ), polymerase θ (PolQ)) [34]. Interestingly, a recent Bohr et al. study demonstrated that PolB was more efficient in single nucleotide gap-filling during mtBER repair than PolG [35]. In our study, the overall enzymatic activity of mitochondrial polymerases has been examined.

The total polymerases activity was observed on the radiogram as bands corresponding to oligonucleotides fragments denoted as SSB+1 (1NU inserted) or SSB+2 (2NU inserted) (Figure 2). Gap-filling was detected for the majority of investigated substrate oligonucleotides (both isomers of cdA and cdG) (Figure 2, Figure S2, S3, S8 and S9). For the dU0 and dU+1 position, no DNA synthesis occurred, which was in agreement with the previous study concerning NE [20]. However, RcdG/dU0 showed 1NU insertion in all experimental repeats (Figure S9) and incidental SSB+1 band was observed for ScdG/dU0 (Figure S8) and RcdA/dU0 (Figure S3). It may indicate the action of polymerases such as PrimPol (capable of bypassing bulky lesions e.g., oxidative and UV-induced lesions or cyclobutane pyrimidine dimers), Rev3 (capable of bypassing UV-induced DNA damage), or PolQ (capable of inserting new nucleobases opposite AP site or thymine glycol on the template strand) [34]. The majority of examined oligonucleotides revealed incorporation of only 1NU, which can indicate that SP-BER is the main repair pathway in mitochondria for the presented model of bi-stranded CDL. Surprisingly, insertion of 2NU was observed for ScdG/dU−1 and RcdG/dU−7. Such difference in distance and between isomers may come from the fact, that 5′*R* and 5′*S* can force different structural changes of the double helix. Interestingly, for the nuclear extract experiments, bands representing SSB+2 were also noted in individual experiments, but for different substrates than those detected in mitochondria (i.e., RcdA/dU−7, RcdA/dU+10, RcdG/dU+10, ScdG/dU+10, and RcdG/dU-4) [20].

The total strand elongation (gap-filling) efficiency increased in the following order (data compared for 6 h reaction time)—cdA: +4 < +7 < −4 < −1 < −7; ScdG: +4 < +7 < −1 < −4 < −7; RcdG: +4 < −7 < −1 < −4 < 0 < +7 (Figures S4, S5, S10 and S11, Tables S5 and S6). From the ME activity point of view, the most interesting is position dU-7, which revealed the highest level of polymerases activity for ScdA, RcdA, and ScdG. These results are contrary to NE activity, where dU−7 had shown second to lowest efficiency of strand elongation [20]. At the same time, RcdG/dU−7 showed second to lowest polymerase activity in ME (the lowest in NE [20]).

Based on the current and previous study of bi-stranded CDL, the general trend seems to be maintained for nucleus and mitochondria—gaps located on the 5′-end side (positive numbers) of cdPu are worse substrates for mitochondrial polymerases than those located on its 3′-end side (negative numbers). Also, oligonucleotides with gaps in position dU+4 revealed the worst efficiency of strand elongation, which has been previously noted for experiments considering xrs5 cell nuclear extracts [10,20]. When comparing different diastereomers, the overall rate of DNA synthesis was up to 8% higher for RcdA than ScdA (except for ScdA/dU+4) and 21–66% higher for ScdG than RcdG (except for ScdG/dU+7) (Figures S7 and S13, Tables S5 and S7). These observations may point to the ability of mitochondrial polymerases to bypass RcdA, but not ScdA. Interestingly, the polymerases seem to act inversely for cdG diastereomers, which was also observed in earlier studies with NE [20]. In relation to Control 1 (35.16% efficiency after 6 h), the strand elongation was more efficient for oligonucleotides containing gaps in positions −1, −4, and −7 (both isomers) (Table S5). For the ds-oligos containing RcdG within the investigated dsCDL, only RcdG/dU+7 surpassed the control level, while in the case of ScdG, gaps located in positions denoted as dU−1, dU−4, dU−7, and dU+7 have shown higher polymerases’ gap-filling activity than Control 1 (Table S7). As one nucleotide shift may impact the DNA repair differently, it is of high importance to investigate these mitochondrial mechanisms in more detail.

## 3. Materials and Methods

### 3.1. The Substrate Oligonucleotides

The 40-mer oligonucleotides used in the study containing AP sites located on the opposite strand and distanced from 1 to 7 bases in both directions from cdPu were prepared as described previously [20]. Briefly, synthetic oligos were synthesized and HPLC-purified in the Bioorganic Chemistry Department of the Polish Academy of Science (Lodz, Poland) which was followed by mass spectrometry analysis. Their stability in the experimental conditions was also examined. Here, chosen duplexes were tested for their stability in the mitochondrial extracts (ME) to ensure no additional interactions with mitochondrial proteins fraction (Figure S1). Single-stranded oligonucleotides were 5′-end-labeled with 2 μCi of [γ-^32^P]ATP, hybridized with the suitable complementary strand (Table S1), and treated with uracil-DNA glycosylase (UDG) to convert 2′-deoxyuridine residues (dU) to the AP sites. It was followed by the human apurinic/apyrimidinic endonuclease (hAPE1) treatment to confirm AP site formation (APE1 catalyzed the hydrolysis of DNA at AP sites creating single-strand breaks (SSB), which were observed as shorter oligo fragments on the gel). Each step of substrate preparation was verified on the 15% native or denaturing polyacrylamide gel, as reported previously [20]. The full sequences of oligonucleotides are presented in Supplementary Materials (Table S1).

### 3.2. Preparation of the Mitochondrial Extracts

The ME was derived from the Ku80-deficient xrs5 cell line (X-ray sensitive Chinese hamster ovarian mutant cell line), which allows avoiding Ku80 binding to SSB or linear DNA termini. The cells were purchased from ATCC (CRL-2348, Manassas, VA, USA) and cultured in MEM medium (Gibco) with 10% FBS (Biowest, Riverside, MO, USA) supplementation. The cells were harvested in exponential phase and after pelleting, they were treated with Mitochondria Isolation Kit for Cultured Cells (ThermoFisher Scientific, Waltham, MA, USA) according to the manufacturer’s protocol. The whole mitochondria fraction (WM) was stored at −80 °C for no longer than 6 months. The extract was prepared immediately before use by addition of 100 μL of the lysis buffer (300 mM KCl, 1% NP-40) and protease inhibitors (to final volume 1×, ThermoFisher, Rockville, IL, USA) to the pellet of WM, incubation in 4 °C for 60 min with occasional mixing and followed by centrifugation (13,000× *g*, 4 °C, 5 min). The protein concentration in the obtained supernatants (ME) was tested using colorimetric Pierce™ 660 nm Protein Assay (ThermoFisher Scientific, Waltham, MA, USA) and was found at 18.0–19.0 mg/mL. To evaluate the quality of ME in the context of proteins involved in mtBER and to exclude the possibility of contamination with the nuclear BER proteins, the functional activity test of mitochondrial extract has been performed (Figure S14). The total repair activity of ME was assessed by comparison with total repair activity of the WM, nuclear extract (NE), and cytoplasmic extract (CE) of xrs5 cell line using Control 1 (ds-oligonucleotide with single AP site lesion). The NE and CE demonstrated enzymatic activities for strand incision, elongation, and reconstitution (CE showed also strand degradation resulting from exonucleolytic activity). The ME exhibited endonucleolytic and polymerase activity responsible for strand incision and elongation, respectively (Figure S14). The WM showed no enzymatic activity, which led to the conclusion that no nuclear contamination was present in the sample prior to lysis of WM. Hence, enzymatic activities observed in ME repair assays were a consequence of mitochondrial protein release and described solely mitochondrial repair activity.

### 3.3. Repair Assays

The 5′-^32^P-end-labeled double-stranded oligonucleotides (200 cps) were incubated with 20 μg of ME. Titration studies were performed to determine the optimal amount of ME for the repair assays (data not shown). Reactions were carried out in repair buffer (70 mM Tris-HCl (pH 7.5), 5 mM MgCl_2_,10 mM DTT, 4 mM ATP, 40 mM phosphocreatine, 1.6 μg/mL phosphocreatine kinase, 0.1 mM dATP, 0.1 mM dCTP, 0.1 mM dGTP, and 0.1 mM dTTP) at 37 °C for 0, 0.5, 3, and 6 h. After the set time, reactions were stopped and examined via PAGE electrophoresis, as described previously [20]. All experiments were repeated three times for consistency and quantified as reported previously using Quantity One software [20]. Briefly, the activity of proteins was expressed as the percentage of the overall intensity of all bands observed within one lane; results were compared with Control 1 (containing only a single AP site, Table S1) to compensate for the slight differences in enzymatic activities between repeats.

## 4. Conclusions

Mitochondrial repair of clustered DNA lesions containing 5′,8-cyclo-2′-deoxypurines is barely understood. Due to difficulties with investigating solely mitochondrial protein fractions many questions about their function remain unanswered. On the other hand, the repair of mtDNA is crucial for energy metabolism and cell survival. CdPus, as a potential biomarker of oxidative DNA damage, may be considered in the future as a diagnostic tool. As cdPus affect DNA structure, their presence in mtDNA can lead to impaired genome maintenance [10].

The model oligonucleotides containing CDL with AP site on one strand and cdPu on the complementary strand separated by 1–7 bp were investigated in this study. Here, it has been shown that the presence of cdPu influences the two initial stages of mtBER repair (strand incision and elongation) of the accompanying lesion (AP site) within the bi-stranded CDL. The results presented in this article are a valuable complement of observations noted for xrs5 cells nuclear extracts [20].

1. In this study, it has been shown for the first time the efficiency of the initial mtBER steps depends on the distance between AP site and cdPus within bi-stranded CDL, and the type and diastereomeric form of the cdPu.
2. In all cases, mitochondrial strand incision and gap-filling were detected for AP sites accompanied by cdPus within bi-stranded CDL.
3. The strand incision step of mtBER was enhanced in the presence of cdPu within the cluster compared to Control 1 (single AP site).
4. AP site incision was more efficient when AP site was accompanied by RcdA or ScdG than by ScdA or RcdG.
5. The gap-filling step of mtBER was inhibited for AP sites located on the 5′-end side of cdPus while for AP sites located on the 3′-end side of cdPus was enhanced compared to Control 1 (single AP site).
6. AP sites located in positions denoted as dU0 and dU+1 inhibit endonuclease and polymerases activity in ME, which aligns with previous observations for NE [20].
7. Both investigated mtBER stages (strand incision and elongation) showed lower efficiency for AP sites located on the 5′-end side of cdPus, compared to those on the 3′-end side of cdPus. It may be assumed that mtBER is slowed down for AP sites located on the 5′-end side of cdPus.

The mtBER initiation is slower than in the nucleus probably due to the protein translocation from cytosol or intermembrane space before the starting point of the repair process.

Due to a low number of studies on mitochondrial proteins involved in mtBER, further studies are necessary. Despite the results we have managed to obtain and present, it remains unexplained why the presence of a specific isomer of individual cdPus at certain distances from another lesion in CDL facilitates/enables the performance of repair enzymes’ functions and other positions have an inhibitory effect. Nevertheless, indicating which variants of CDL (cdPu type, isomer type, and interlesion distance within the cluster) and how they affect repair is a good starting point for further research, e.g., computational and structural analysis of DNA-protein complexes. It may especially contribute to the field of mitochondrial diseases that are connected to the formation of large amounts of hydroxyl radical that causes cdPu formation.

## Data Availability

Not applicable.

## Supplementary Materials

The following are available online, Table S1: The sequences of double-stranded substrate oligonucleotides, Table S2: Endonuclease activity—Control 1. Raw numerical data, Table S3: Polymerase activity—Control 1. Raw numerical data, Table S4: Endonuclease activity—ScdA and RcdA. Raw numerical data, Table S5: Polymerase activity—ScdA and RcdA. Raw numerical data, Table S6: Endonuclease activity—ScdG and RcdG. Raw numerical data, Table S7: Polymerase activity—ScdG and RcdG. Raw numerical data, Figure S1: The stability of “matrix” oligonucleotides, Figures S2 and S3: The autoradiograms—ScdA and RcdA, Figures S4 and S5: Graphical representation of the results for ScdA and RcdA, Figure S6: Endonuclease activity [%] of ScdA vs. RcdA—comparison of individual strands, Figure S7: Polymerase activity [%] of ScdA vs. RcdA—comparison of individual strands, Figures S8 and S9: The autoradiograms—ScdG and RcdG, Figures S10 and S11: Graphical representation of the results for ScdG and RcdG, Figure S12: Endonuclease activity [%] of ScdG vs. RcdG—comparison of individual strands, Figure S13: Polymerase activity [%] of ScdG vs. RcdG—comparison of individual strands; Figure S14: Functional activity test of mitochondrial extracts.

## Sample Availability

Samples of the compounds are not available from the authors.

## References

[1] Boguszewska K., Szewczuk M., Kaźmierczak-Barańska J., Karwowski B.T. (2020). The similarities between human mitochondria and bacteria in the context of structure, genome, and base excision repair system. Molecules.

[2] Garcia I., Jones E., Ramos M., Innis-Whitehouse W., Gilkerson R. (2017). The little big genome: The organization of mitochondrial DNA. Front. Biosci. (Landmark).

[3] Wasilewski M., Chojnacka K., Chacinska A. (2017). Protein trafficking at the crossroads to mitochondria. Biochim. Biophys. Acta—Mol. Cell Res..

[4] Topf U., Uszczynska-Ratajczak B., Chacinska A. (2019). Mitochondrial stress-dependent regulation of cellular protein synthesis. J. Cell Sci..

[5] Sharma P., Sampath H. (2019). Mitochondrial DNA integrity: Role in health and disease. Cells.

[6] López-Lluch G., Santos-Ocaña C., Sánchez-Alcázar J.A., Fernández-Ayala D.J.M., Asencio-Salcedo C., Rodríguez-Aguilera J.C., Navas P. (2015). Mitochondrial responsibility in ageing process: Innocent, suspect or guilty. Biogerontology.

[7] Martín Giménez V.M., de las Heras N., Ferder L., Lahera V., Reiter R.J., Manucha W. (2021). Potential effects of melatonin and micronutrients on mitochondrial dysfunction during a cytokine storm typical of oxidative/inflammatory diseases. Diseases.

[8] Bader G., Enkler L., Araiso Y., Hemmerle M., Binko K., Baranowska E., de Craene J.O., Ruer-Laventie J., Pieters J., Tribouillard-Tanvier D. (2020). Assigning mitochondrial localization of dual localized proteins using a yeast bi-genomic mitochondrial-split-Gfp. eLife.

[9] Lindahl T. (1993). Instability and decay of the primary structure of DNA. Nature.

[10] Karwowski B.T., Bellon S., O’Neill P., Lomax M.E., Cadet J. (2014). Effects of (5′*S*)-5′,8-Cyclo-2′-deoxyadenosine on the base excision repair of oxidatively generated clustered DNA damage. A Biochemical and theoretical study. Org. Biomol. Chem..

[11] Kuraoka I., Bender C., Romieu A., Cadet J., Wood R.D., Lindahl T. (2000). Removal of oxygen free-radical-induced 5′,8-purine cyclodeoxynucleosides from DNA by the nucleotide excision-repair pathway in human cells. Proc. Natl. Acad. Sci. USA.

[12] Jaruga P., Dizdaroglu M. (2008). 8,5′-Cyclopurine-2′-deoxynucleosides in DNA: Mechanisms of formation, measurement, repair and biological effects. DNA Repair.

[13] Kusumoto R., Masutani C., Iwai S., Hanaoka F. (2002). Translesion synthesis by human DNA polymerase η across thymine glycol lesions. Biochemistry.

[14] Brooks P.J. (2017). The cyclopurine deoxynucleosides: DNA repair, biological effects, mechanistic insights, and unanswered questions. Free Radic. Biol. Med..

[15] Jasti V.P., Das R.S., Hilton B.A., Weerasooriya S., Zou Y., Basu A.K. (2011). (5′*S*)-8,5′-cyclo-2′-deoxyguanosine is a strong block to replication, a potent pol V-dependent mutagenic lesion, and is inefficiently repaired in *Escherichia coli*. Biochemistry.

[16] Jiang Z., Xu M., Lai Y., Laverde E.E., Terzidis M.A., Masi A., Chatgilialoglu C., Liu Y. (2015). Bypass of a 5′,8-cyclopurine-2′-deoxynucleoside by DNA polymerase β during DNA replication and base excision repair leads to nucleotide misinsertions and DNA strand breaks. DNA Repair.

[17] Lomax M.E., Cunniffe S., O’Neill P. (2004). Efficiency of repair of an abasic site within DNA clustered damage sites by mammalian cell nuclear extracts. Biochemistry.

[18] David-Cordonniert M.-H., Laval J., O’Neil P. (2000). Clustered DNA damage, influence on damage excision by XRS5 nuclear extracts and escherichia coli Nth and Fpg proteins. J. Biol. Chem..

[19] Eccles L.J., Menoni H., Angelov D., Lomax M.E., O’Neill P. (2015). Efficient cleavage of single and clustered AP site lesions within mono-nucleosome templates by CHO-K1 nuclear extract contrasts with retardation of incision by purified APE1. DNA Repair.

[20] Boguszewska K., Szewczuk M., Kaźmierczak-Barańska J., Karwowski B.T. (2021). How (5′*S*) and (5′*R*) 5′,8-cyclo-2′-deoxypurines affect base excision repair of clustered DNA damage in nuclear extracts of Xrs5 cells? A biochemical study. Cells.

[21] Karwowski B.T. (2019). The influence of (5′*R*)- and (5′*S*)-5′,8-cyclo-2′-deoxyadenosine on UDG and HAPE1 activity. Tandem lesions are the base excision repair system’s nightmare. Cells.

[22] Izumi T., Mellon I. (2016). Base Excision Repair and Nucleotide Excision Repair.

[23] Singatulina A.S., Pestryakov P.E. (2016). Mechanisms of DNA repair in mitochondria. Biopolym. Cell.

[24] Alexeyev M., Shokolenko I., Wilson G., LeDoux S. (2013). The maintenance of mitochondrial DNA integrity—Critical analysis and update. Cold Spring Harb. Perspect. Biol..

[25] Omar García-Lepe U., Ma Bermúdez-Cruz R. (2019). Mitochondrial genome maintenance: Damage and repair pathways. DNA Repair-An Update.

[26] Karwowski B.T. (2021). (5′*S*) 5′,8-cyclo-2′-deoxyadenosine cannot stop BER. Clustered DNA lesion studies. Int. J. Mol. Sci..

[27] Jaruga P., Rozalski R., Jawien A., Migdalski A., Olinski R., Dizdaroglu M. (2012). DNA damage products (5′*R*)- and (5′*S*)-8,5′-cyclo- 2′-deoxyadenosines as potential biomarkers in human urine for atherosclerosis. Biochemistry.

[28] Kant M., Akış M., Çalan M., Arkan T., Bayraktar F., Dizdaroglu M., İşlekel H. (2016). Elevated urinary levels of 8-oxo-2′-deoxyguanosine, (5′*R*)- and (5′*S*)-8,5′-cyclo-2′-deoxyadenosines, and 8-iso-prostaglandin F2α as potential biomarkers of oxidative stress in patients with prediabetes. DNA Repair.

[29] Jaruga P., Dizdaroglu M. (2010). Identification and quantification of (5′*R*)- and (5′*S*)-8,5′-cyclo-2′-deoxyadenosines in human urine as putative biomarkers of oxidatively induced damage to DNA. Biochem. Biophys. Res. Commun..

[30] Chacinska A., Koehler C.M., Milenkovic D., Lithgow T., Pfanner N. (2009). Importing mitochondrial proteins: Machineries and mechanisms. Cell.

[31] Barchiesi A., Bazzani V., Tolotto V., Elancheliyan P., Wasilewski M., Chacinska A., Vascotto C. (2020). Mitochondrial oxidative stress induces rapid intermembrane space/matrix translocation of apurinic/apyrimidinic endonuclease 1 protein through TIM23 complex. J. Mol. Biol..

[32] Pednekar V., Weerasooriya S., Jasti V.P., Basu A.K. (2014). Mutagenicity and genotoxicity of (5′*S*)-8,5′-cyclo-2′- deoxyadenosine in Escherichia coli and replication of (5′*S*)-8,5′-cyclopurine-2′-deoxynucleosides in vitro by DNA polymerase Iv, exo-free Klenow fragment, and Dpo4. Chem. Res. Toxicol..

[33] Xu M., Lai Y., Jiang Z., Terzidis M.A., Masi A., Chatgilialoglu C., Liu Y. (2014). A 5′, 8-cyclo-2′-deoxypurine lesion induces trinucleotide repeat deletion via a unique lesion bypass by DNA polymerase β. Nucleic Acids Res..

[34] Krasich R., Copeland W.C. (2017). DNA Polymerases in the mitochondria: A critical review of the evidence. Front. Biosci..

[35] Baptiste B.A., Baringer S.L., Kulikowicz T., Sommers J.A., Croteau D.L., Brosh R.M., Bohr V.A. (2021). DNA polymerase β outperforms DNA polymerase γ in key mitochondrial base excision repair activities. DNA Repair.

