# Supplementary material for: The Influence of 5′,8-Cyclo-2′-deoxypurines on the Mitochondrial Repair of Clustered DNA Damage in Xrs5 Cells: The Preliminary Study
Source: Molecules. 2021 Nov 22;26(22):7042. doi: 10.3390/molecules26227042 (PMC8623968; doi:10.3390/molecules26227042)
Supplement: Supplementary file 1 [file molecules-26-07042-s001.zip › molecules-1480139-supplementary.pdf]

The influence of 5′,8-cyclo-2′-deoxypurines on the mitochondrial repair of clustered DNA damage in xrs5 cells. The preliminary study.

Supplementary Materials

Page	Item	Title
2	**Table S1**	The sequences of double-stranded substrate oligonucleotides.
3	**Figure S1**	The stability of „matrix" oligonucleotides.
4	**Table S2**	Endonuclease activity – Control 1. Raw numerical data.
4	**Table S3**	Polymerase activity – Control 1. Raw numerical data.
5	**Figure S2**	The autoradiograms – ScdA.
6	**Figure S3**	The autoradiograms – RcdA.
7	**Table S4**	Endonuclease activity – ScdA and RcdA. Raw numerical data.
8	**Table S5**	Polymerase activity – ScdA and RcdA. Raw numerical data.
9-10	**Figure S4**	Graphical representation of the results for ScdA.
10-11	**Figure S5**	Graphical representation of the results for RcdA.
12-15	**Figure S6**	Endonuclease activity [%] of ScdA vs. RcdA – comparison of individual strands.
16-19	**Figure S7**	Polymerase activity [%] of ScdA vs. RcdA – comparison of individual strands.
20	**Figure S8**	The autoradiograms – ScdG.
21	**Figure S9**	The autoradiograms – RcdG.
22	**Table S6**	Endonuclease activity – ScdG and RcdG. Raw numerical data.
23	**Table S7**	Polymerase activity – ScdG and RcdG. Raw numerical data.
24-25	**Figure S10**	Graphical representation of the results for ScdG.
25-26	**Figure S11**	Graphical representation of the results for RcdG.
27-30	**Figure S12**	Endonuclease activity [%] of ScdG vs. RcdG – comparison of individual strands.
30-34	**Figure S13**	Polymerase activity [%] of ScdG vs. RcdG – comparison of individual strands.
35	**Figure S14**	Functional activity test of mitochondrial extract.

Table S1. The sequences of double-stranded substrate oligonucleotides containing 2′-deoxyuridine (dU) and 5′,8-cyclo-2′-deoxyadenosine (cdA) or 5′,8-cyclo-2′-deoxyguanosine (cdG).

	Oligonucleotide	Sequence
ScdA / RcdA	Control 1	*5′-CTCTTGTCAGGAATATTGTCUCTATGCTCCCACCAAAGGC-3′<br>3′-GAGAACAGTCCTTATAACAGAGATACGAGGGTGGTTTCCG-5′
	Control 2	*5′-GCCTTTGGTGGGAGCATAGXGACAATATTCCTGACAAGAG-3′<br>3′-CGGAAACCACCCTCGTATCTCTGTTATAAGGACTGTTCTC-5′
	dU −7	*5′-CTCTTGTCAGGAAUATTGTCTCTATGCTCCCACCAAAGGC-3′<br>3′-GAGAACAGTCCTTATAACAGXGATACGAGGGTGGTTTCCG-5′
	dU −4	*5′-CTCTTGTCAGGAATATUGTCTCTATGCTCCCACCAAAGGC-3′<br>3′-GAGAACAGTCCTTATAACAGXGATACGAGGGTGGTTTCCG-5′
	dU −1	*5′-CTCTTGTCAGGAATATTGTUTCTATGCTCCCACCAAAGGC-3′<br>3′-GAGAACAGTCCTTATAACAGXGATACGAGGGTGGTTTCCG-5′
	dU 0	*5′-CTCTTGTCAGGAATATTGTCUCTATGCTCCCACCAAAGGC-3′<br>3′-GAGAACAGTCCTTATAACAGXGATACGAGGGTGGTTTCCG-5′
	dU +1	*5′-CTCTTGTCAGGAATATTGTCTUTATGCTCCCACCAAAGGC-3′<br>3′-GAGAACAGTCCTTATAACAGXGATACGAGGGTGGTTTCCG-5′
	dU +4	*5′-CTCTTGTCAGGAATATTGTCTCTAUGCTCCCACCAAAGGC-3′<br>3′-GAGAACAGTCCTTATAACAGXGATACGAGGGTGGTTTCCG-5′
	dU +7	*5′-CTCTTGTCAGGAATATTGTCTCTATGCUCCCACCAAAGGC-3′<br>3′-GAGAACAGTCCTTATAACAGXGATACGAGGGTGGTTTCCG-5′
ScdG / RcdG	Control 1	*5′-CTCTTGTCAGGAATATTGTCUCTATGCTCCCACCAAAGGC-3′<br>3′-GAGAACAGTCCTTATAACAGAGATACGAGGGTGGTTTCCG-5′
	Control 2	*5′-GCCTTTGGTGGGAGCATAGYGACAATATTCCTGACAAGAG-3′<br>3′-CGGAAACCACCCTCGTATCTCTGTTATAAGGACTGTTCTC-5′
	dU −7	*5′-CTCTTGTCAGGAAUATTGTCCCTATGCTCCCACCAAAGGC-3′<br>3′-GAGAACAGTCCTTATAACAGYGATACGAGGGTGGTTTCCG-5′
	dU −4	*5′-CTCTTGTCAGGAATATUGTCCCTATGCTCCCACCAAAGGC-3′<br>3′-GAGAACAGTCCTTATAACAGYGATACGAGGGTGGTTTCCG-5′
	dU −1	*5′-CTCTTGTCAGGAATATTGTUCCTATGCTCCCACCAAAGGC-3′<br>3′-GAGAACAGTCCTTATAACAGYGATACGAGGGTGGTTTCCG-5′
	dU 0	*5′-CTCTTGTCAGGAATATTGTCUCTATGCTCCCACCAAAGGC-3′<br>3′-GAGAACAGTCCTTATAACAGYGATACGAGGGTGGTTTCCG-5′
	dU +1	*5′-CTCTTGTCAGGAATATTGTCCUTATGCTCCCACCAAAGGC-3′<br>3′-GAGAACAGTCCTTATAACAGYGATACGAGGGTGGTTTCCG-5′
	dU +4	*5′-CTCTTGTCAGGAATATTGTCCCTAUGCTCCCACCAAAGGC-3′<br>3′-GAGAACAGTCCTTATAACAGYGATACGAGGGTGGTTTCCG-5′
	dU +7	*5′-CTCTTGTCAGGAATATTGTCCCTATGCUCCCACCAAAGGC-3′<br>3′-GAGAACAGTCCTTATAACAGYGATACGAGGGTGGTTTCCG-5′

U – represents dU as an AP site (after treatment with UDG); X – represents (5′*S*)-5′,8-cyclo-2′-deoxyadenosine (ScdA) or (5′*R*)-5′,8-cyclo-2′-deoxyadenosine (RcdA); Y – represents (5′*S*)-5′,8-cyclo-2′-deoxyguanosine (ScdG) or (5′*R*)-5′,8-cyclo-2′-deoxyguanosine (RcdG); * – represents the 5′-end-labeled strand with [γ-^{32}P]ATP

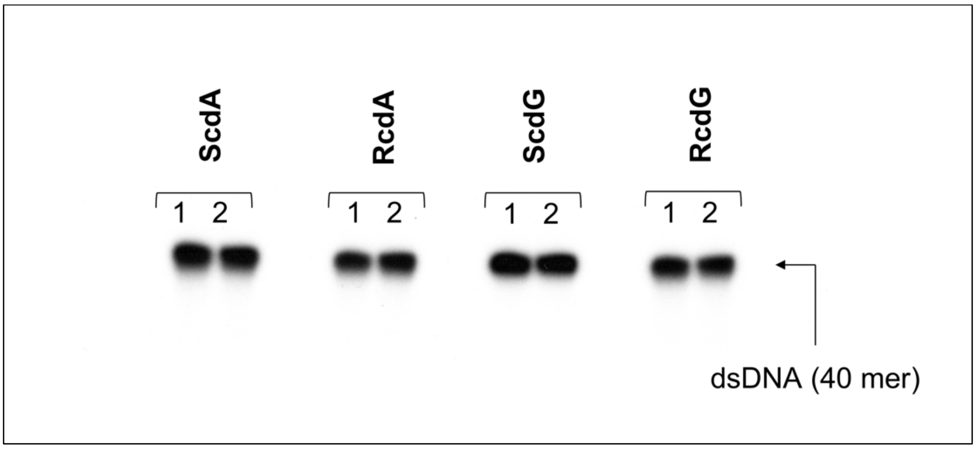

Figure S1. The stability of „matrix" oligonucleotides (Control 2) after treatment with 20 μg of mitochondrial extracts (ME). Each lane number corresponds with different assay time: lane 1 - 0 min; lane 2 - 6 h.

Table S2. Endonuclease activity – Control 1. Raw numerical data of densitometry.

		Time [h]			
		0	0,5	3	6
Strand	Data set	**Endonuclease activity [%]**			
Control 1	1.	1,39	85,92	75,75	72,41
	2.	0,94	82,68	76,83	71,90
	3.	0,31	77,43	73,06	69,80
	4.	0,76	84,68	69,47	59,11
	5.	2,67	70,72	59,86	55,64
	6.	2,42	72,21	62,02	52,42
	7.	1,41	87,65	61,08	51,19
	8.	0,09	74,09	63,99	54,96
	9.	0,00	99,06	83,54	53,02
	Avg	1,35	71,92	63,98	55,75
	SD	1,66	21,93	13,23	11,42

Table S3. Polymerase activity – Control 1. Raw numerical data of densitometry.

		Time [h]			
		0	0,5	3	6
Strand	Data set	**Polymerase activity [%]**			
Control 1	1.	0,00	0,65	12,74	19,65
	2.	0,00	2,07	10,48	19,27
	3.	0,00	0,93	11,08	18,72
	4.	0,04	11,23	26,32	37,15
	5.	0,00	12,05	29,14	34,96
	6.	0,58	6,65	33,60	44,75
	7.	0,00	6,83	35,72	46,86
	8.	0,00	4,75	33,87	44,15
	9.	0,00	0,00	16,28	46,54
	Avg	0,18	5,88	21,65	35,16
	SD	0,47	5,06	10,36	10,53

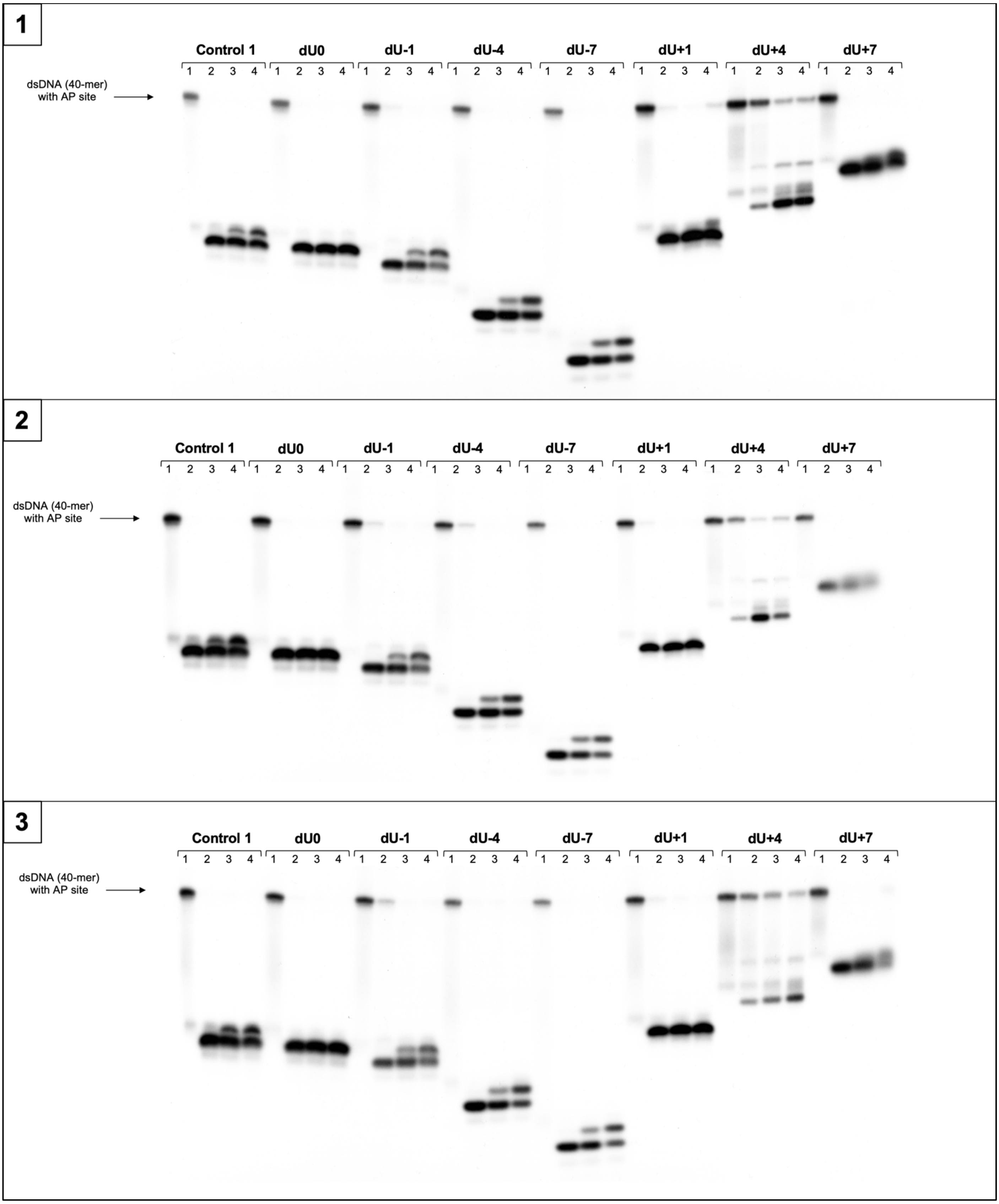

Figure S2. The autoradiograms of denaturing PAGE presenting dsDNA containing clustered damage with AP site in one strand and **ScdA** in the opposing strand. Each lane number indicates different assay time: lane 1 - 0 min; lane 2 - 30 min; lane 3 – 3h; lane 4 – 6h. Three experimental replications are presented.

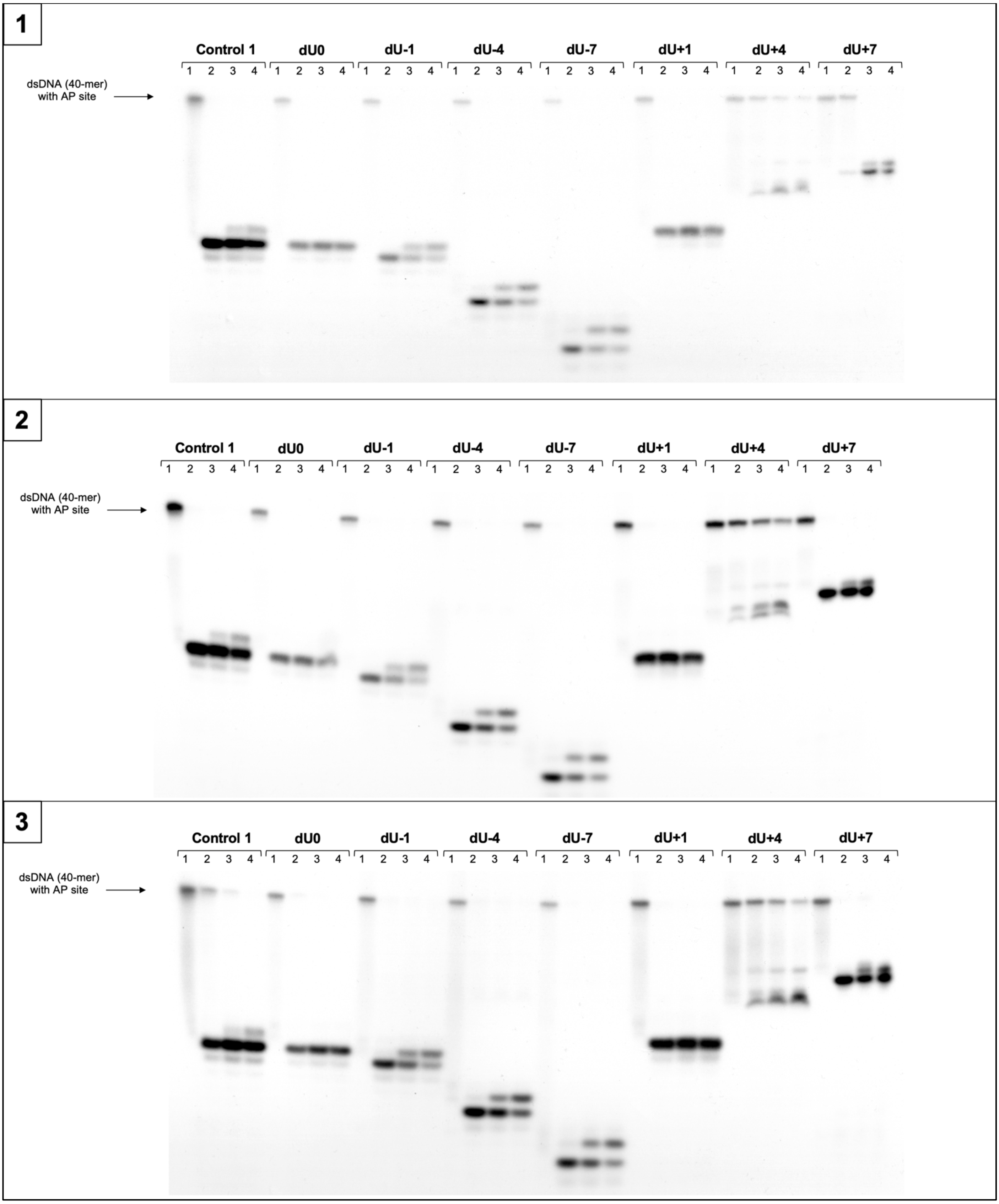

Figure S3. The autoradiograms of denaturing PAGE presenting dsDNA containing clustered damage with AP site in one strand and **RcdA** in the opposing strand. Each lane number indicates different assay time: lane 1 - 0 min; lane 2 - 30 min; lane 3 – 3h; lane 4 – 6h. Three experimental replications are presented.

Table S4. Endonuclease activity – ScdA and RcdA. Raw numerical data of densitometry obtained from Quantity One software.

RcdA		Time [h] 0	0,5	3	6	ScdA		Time [h] 0	0,5	3	6
Strand	Data set	Endonuclease activity [%]				Strand	Data set	Endonuclease activity [%]			
dU0	1.	7,61	86,52	89,54	90,68	dU0	1.	2,41	88,36	87,17	86,87
	2.	4,81	98,53	96,19	99,53		2.	1,44	77,19	77,40	75,55
	3.	3,20	91,53	88,33	87,08		3.	2,14	79,23	77,70	77,85
	Avg	5,21	92,19	91,36	92,43		Avg	2,00	81,60	80,76	80,09
	SD	2,23	6,03	4,23	6,41		SD	0,50	5,95	5,56	5,98
dU-1	1.	0,00	87,13	62,86	45,04	dU-1	1.	3,56	88,02	65,01	45,92
	2.	3,74	99,08	63,51	41,13		2.	1,05	80,86	64,94	47,48
	3.	3,22	81,02	56,42	38,95		3.	2,88	71,05	60,36	45,25
	Avg	2,32	89,08	60,93	41,71		Avg	2,49	79,98	63,44	46,22
	SD	2,02	9,19	3,92	3,09		SD	1,30	8,52	2,66	1,14
dU-4	1.	0,73	95,03	57,62	51,65	dU-4	1.	0,67	89,55	70,80	54,20
	2.	0,00	93,20	67,45	42,47		2.	3,06	87,27	70,39	53,73
	3.	0,74	82,65	64,73	45,40		3.	2,71	88,65	67,32	46,49
	Avg	0,49	90,29	63,27	46,51		Avg	2,15	88,49	69,51	51,47
	SD	0,43	6,68	5,07	4,69		SD	1,29	1,15	1,90	4,32
dU-7	1.	0,00	95,96	63,85	36,73	dU-7	1.	0,00	90,35	64,23	49,59
	2.	0,00	91,19	51,46	37,67		2.	0,96	96,02	68,46	47,22
	3.	4,12	84,93	57,76	37,93		3.	0,91	90,48	70,07	45,66
	Avg	1,37	90,69	57,69	37,44		Avg	0,62	92,28	67,59	47,49
	SD	2,38	5,53	6,20	0,63		SD	0,54	3,23	3,01	1,98
dU+1	1.	0,00	92,47	93,24	93,30	dU+1	1.	0,78	81,26	80,73	67,58
	2.	0,31	92,08	89,43	90,11		2.	0,52	90,49	88,68	87,66
	3.	2,83	86,40	80,96	83,67		3.	1,79	83,76	81,79	82,84
	Avg	1,05	90,32	87,88	89,03		Avg	1,03	85,17	83,73	79,36
	SD	1,55	3,40	6,28	4,91		SD	0,67	4,77	4,32	10,48
dU+4	1.	0,00	25,71	61,62	73,62	dU+4	1.	1,27	32,49	65,11	57,37
	2.	7,21	40,17	69,62	56,70		2.	0,10	31,21	82,32	67,64
	3.	2,19	29,95	48,10	62,93		3.	0,91	33,91	48,46	59,19
	Avg	3,13	31,94	59,78	64,41		Avg	0,76	32,53	65,30	61,40
	SD	3,69	7,44	10,88	8,56		SD	0,60	1,35	16,93	5,48
dU+7	1.	0,34	87,41	80,41	72,03	dU+7	1.	0,59	80,00	64,00	59,18
	2.	2,91	26,95*	79,84	60,60		2.	0,44	87,73	74,19	74,76
	3.	1,68	78,77	66,97	63,10		3.	0,64	76,63	72,02	57,54
	Avg	1,64	83,09	75,74	65,24		Avg	0,56	81,45	70,07	63,82
	SD	1,29	6,11	7,60	6,01		SD	0,10	5,69	5,37	9,50

* Rejected values

Table S5. Polymerase activity – ScdA and RcdA. Raw numerical data of densitometry obtained from Quantity One software.

**RcdA**		Time [h]				**ScdA**		Time [h]			
		0	0,5	3	6			0	0,5	3	6
Strand	Data set	**Polymerase activity [%]**				Strand	Data set	**Polymerase activity [%]**			
	1.	0,00	0,00	0,35	0,21		1.	0,32	2,87	2,90	3,66
	2.	0,00	0,00	0,00	0,00		2.	1,54	8,52	7,47	7,15
dU0	3.	3,12*	0,00	1,31	2,91	dU0	3.	0,13	6,68	7,33	6,79
	Avg	0,00	0,00	0,55	1,04		Avg	0,66	6,02	5,90	5,87
	SD	0,00	0,00	0,68	1,62		SD	0,77	2,88	2,60	1,92
	1.	0,00	3,73	30,12	49,29		1.	0,42	4,56	30,46	50,70
	2.	0,00	0,00	36,19	57,94		2.	0,86	5,70	25,38	46,47
dU-1	3.	0,00	6,30	34,10	52,86	dU-1	3.	1,11	8,38	31,89	49,70
	Avg	0,00	3,34	33,47	53,36		Avg	0,80	6,21	29,24	48,95
	SD	0,00	3,17	3,08	4,35		SD	0,35	1,96	3,42	2,21
	1.	0,00	1,55	38,17	45,40		1.	0,90	2,70	24,24	42,55
	2.	0,00	0,00	27,19	54,04		2.	0,66	3,00	25,33	43,93
dU-4	3.	0,00	7,10	27,82	48,87	dU-4	3.	2,97	4,98	28,10	49,66
	Avg	0,00	2,88	31,06	49,43		Avg	1,51	3,56	25,89	45,38
	SD	0,00	3,74	6,17	4,35		SD	1,27	1,24	1,99	3,77
	1.	0,00	0,00	32,73	58,42		1.	0,01	3,33	31,98	47,54
	2.	0,00	5,12	45,94	58,37		2.	0,00	1,60	29,74	51,25
dU-7	3.	0,00	6,34	33,68	54,37	dU-7	3.	1,75	3,22	25,10	50,76
	Avg	0,00	3,82	37,45	57,05		Avg	0,59	2,72	28,94	49,85
	SD	0,00	3,36	7,37	2,32		SD	1,01	0,97	3,51	2,02
	1.	0,00	0,00	0,00	0,00		1.	0,33	10,39	11,30	21,02
	2.	0,00	0,00	0,42	0,39		2.	0,00	5,03	2,41	3,04
dU+1	3.	0,00	2,67	1,89	1,15	dU+1	3.	0,00	9,27	12,35	9,56
	Avg	0,00	0,89	0,77	0,51		Avg	0,11	8,23	8,69	11,21
	SD	0,00	1,54	1,00	0,58		SD	0,19	2,83	5,46	9,10
	1.	8,18*	4,33	4,30	4,54		1.	0,02	0,13	22,57	29,00
	2.	0,66	12,15	14,27	28,59		2.	0,02	4,48	12,01	16,42
dU+4	3.	0,41	19,59	24,36	19,79	dU+4	3.	0,10	11,82	17,86	24,27
	Avg	0,54	12,02	14,31	17,64		Avg	0,05	5,48	17,48	23,23
	SD	0,18	7,63	10,03	12,17		SD	0,04	5,91	5,29	6,35
	1.	2,41	1,25	14,82	22,23		1.	0,26	18,49	33,96	39,46
	2.	1,98	5,72	17,34	37,97		2.	0,32	8,08	16,15	13,24
dU+7	3.	0,46	14,64	25,45	32,31	dU+7	3.	2,46	16,21	18,78	34,44
	Avg	1,62	7,20	19,20	30,84		Avg	1,02	14,26	22,97	29,05
	SD	1,03	6,82	5,55	7,97		SD	1,25	5,47	9,61	13,92

* Rejected values

A.

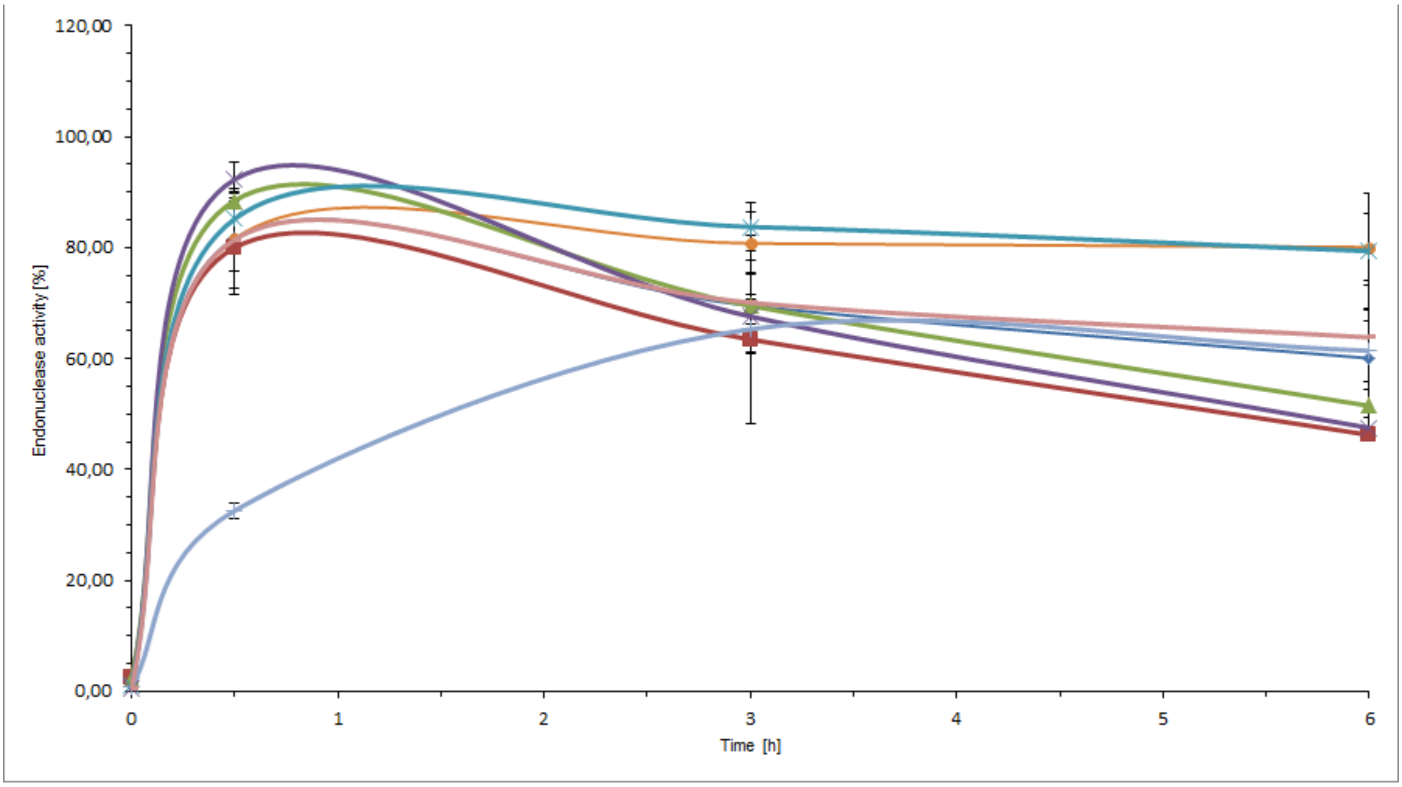

120,00
100,00
80,00
60,00
40,00
20,00
0,00
Endonuclease activity [%]
0
1
2
3
4
5
6
Time [h]

B.

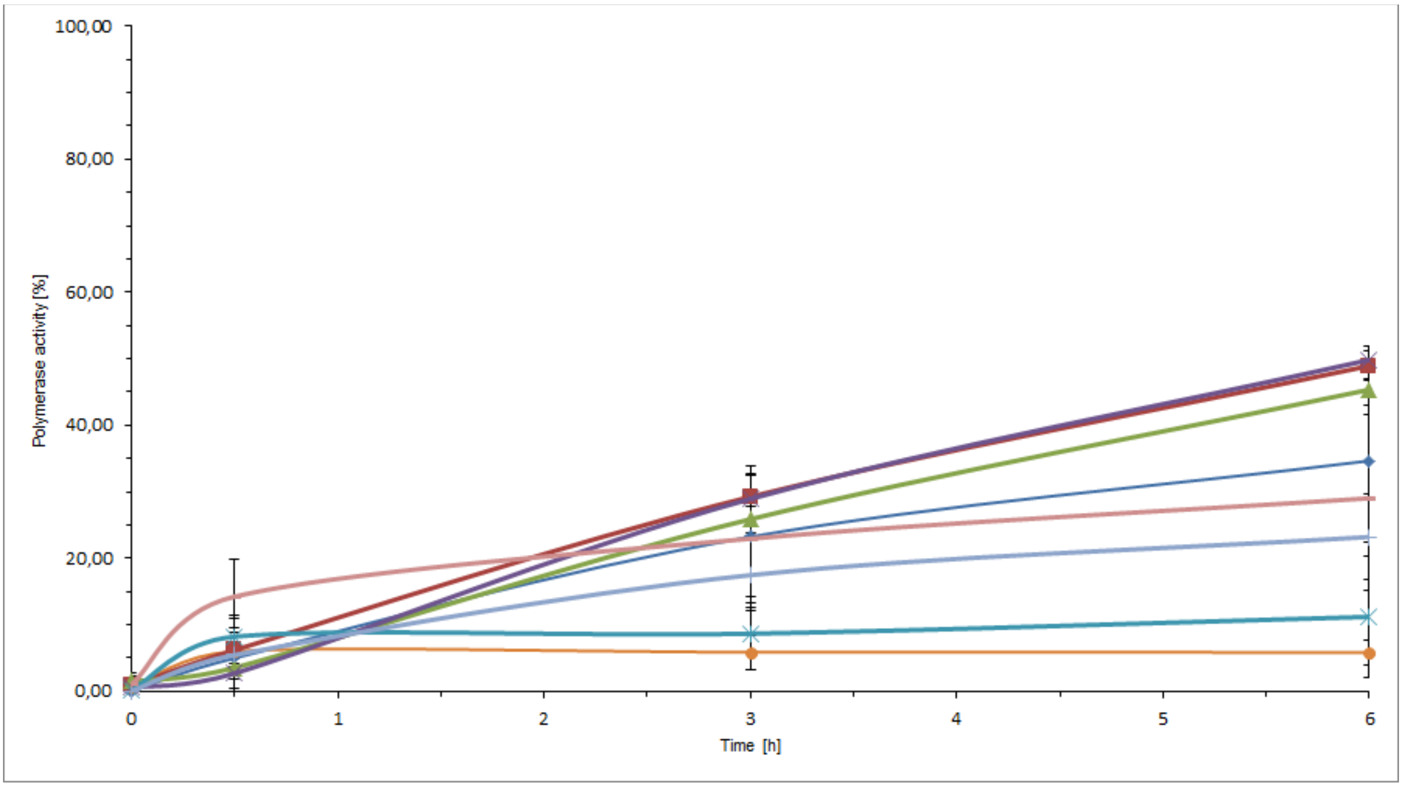

100,00
80,00
60,00
40,00
20,00
0,00
Polymerase activity [%]
0
1
2
3
4
5
6
Time [h]

C.

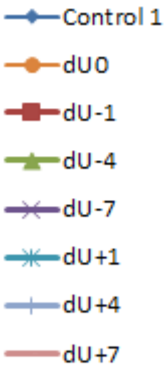

Figure S4. Graphical representation of the results for ScdA. (A) endonuclease activity +SD, (B) polymerase activity + SD; (C) figure legend.

A.

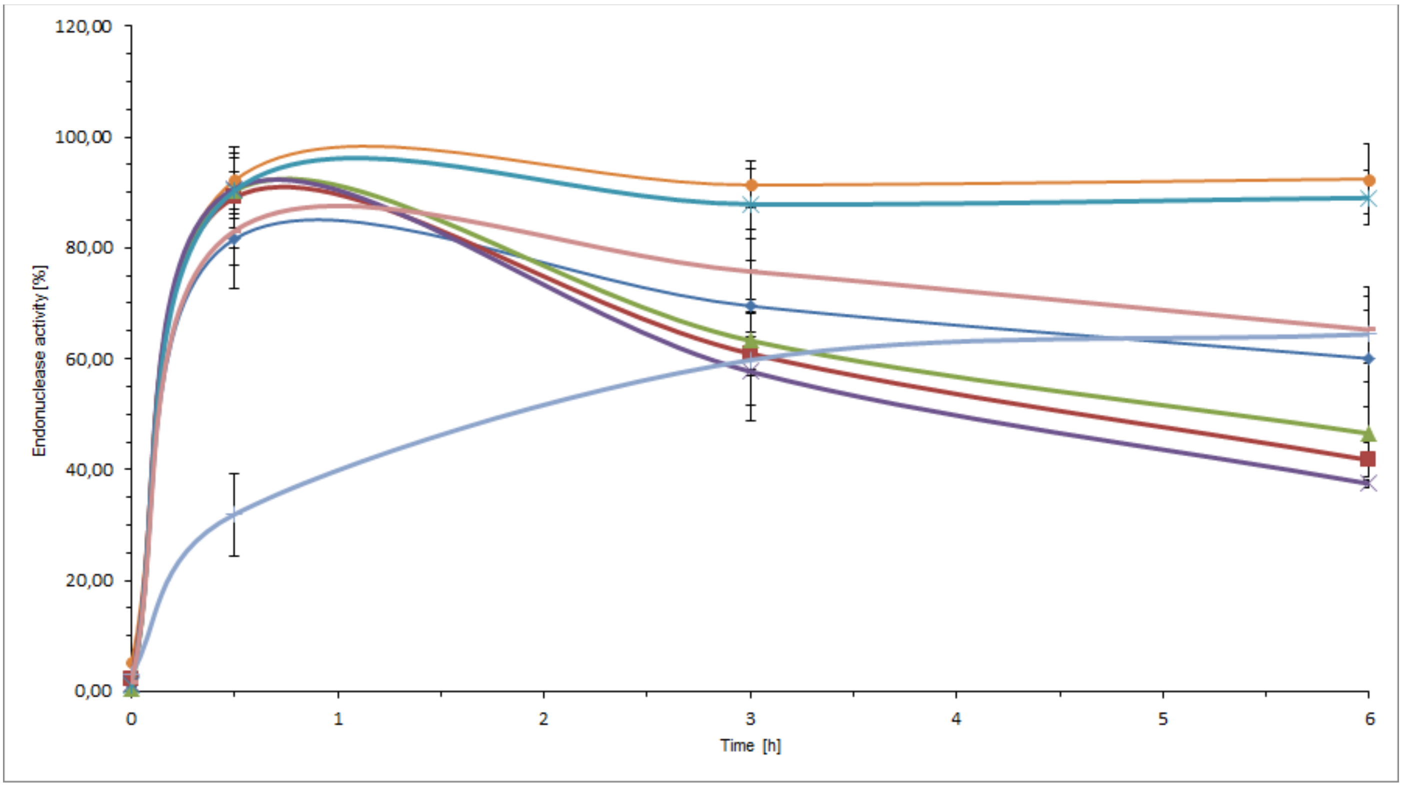

B.

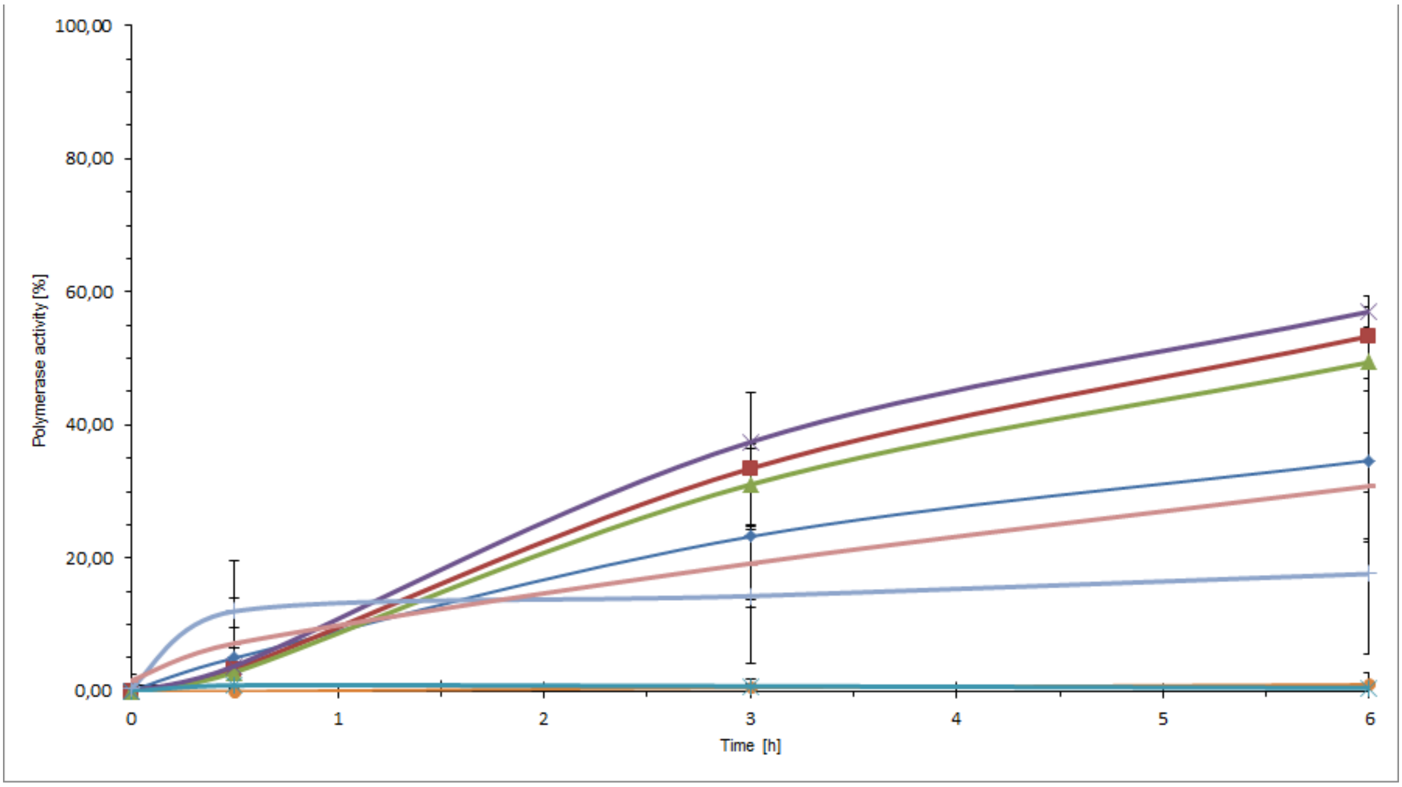

C.

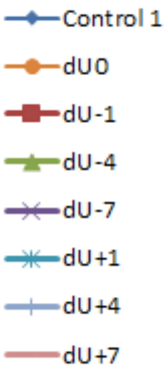

Figure S5. Graphical representation of the results for RcdA. (A) endonuclease activity +SD, (B) polymerase activity + SD; (C) figure legend.

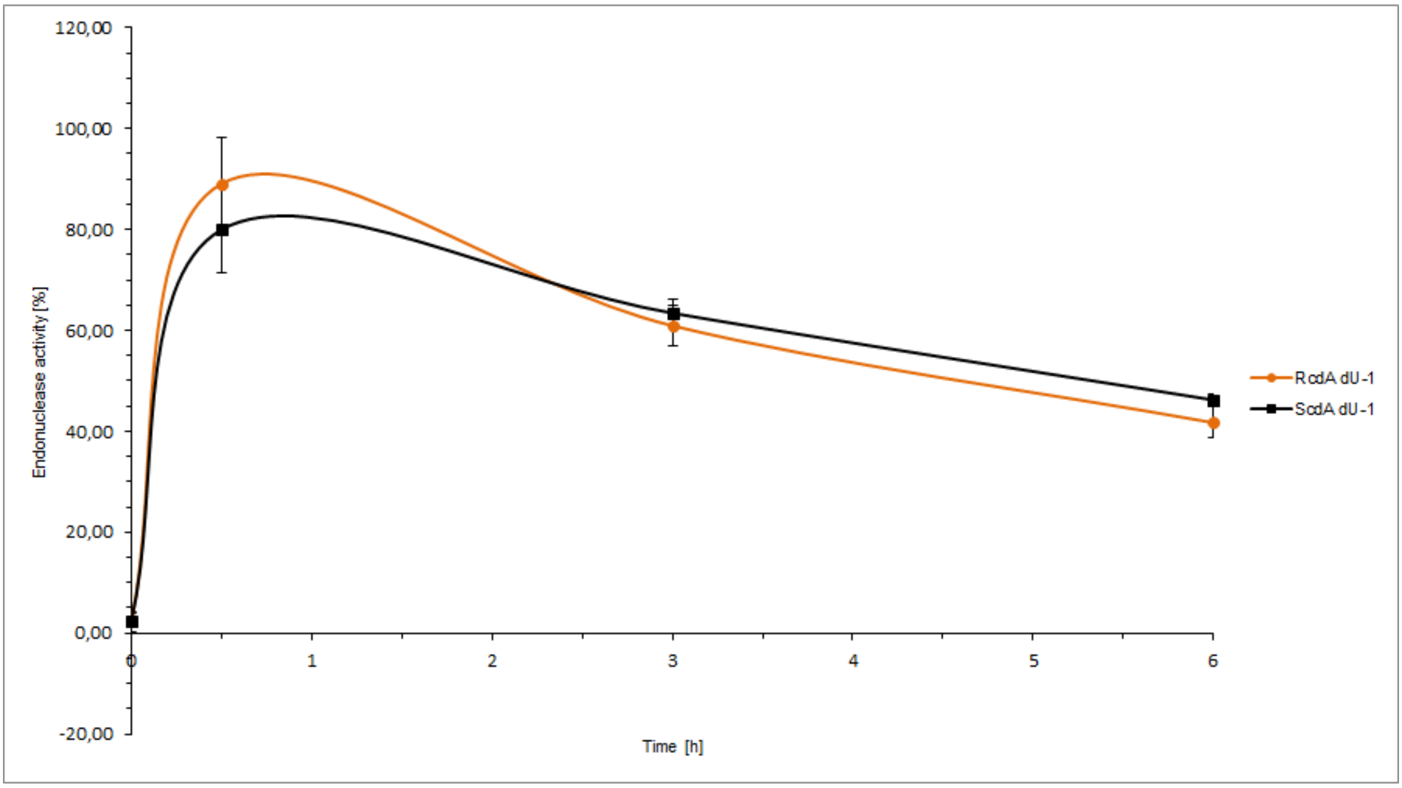
120,00
100,00
80,00
60,00
40,00
20,00
0,00
-20,00
Endonuclease activity [%]
0
1
2
3
4
5
6
Time [h]
RcdA dU-1
ScdA dU-1

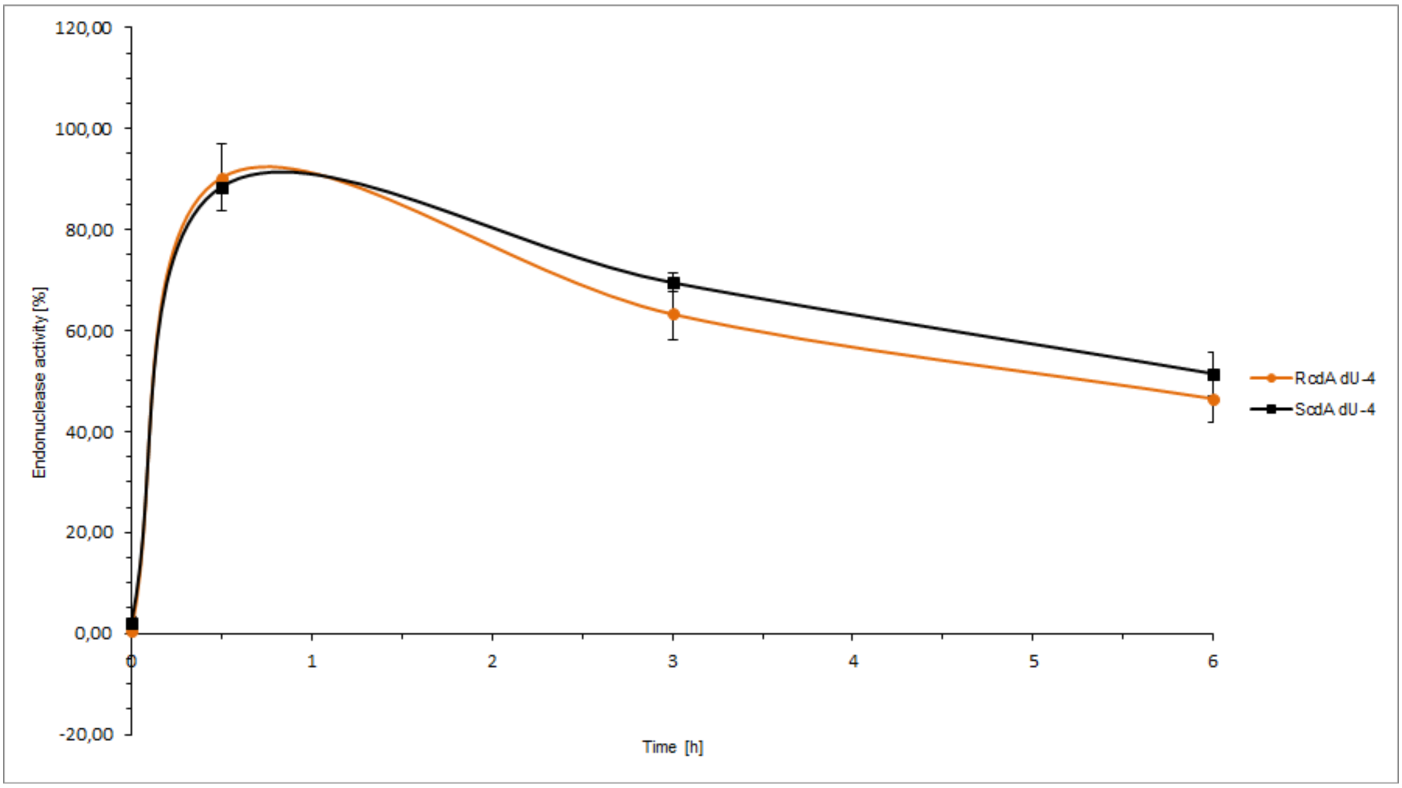
120,00
100,00
80,00
60,00
40,00
20,00
0,00
-20,00
Endonuclease activity [%]
0
1
2
3
4
5
6
Time [h]
RcdA dU-4
ScdA dU-4

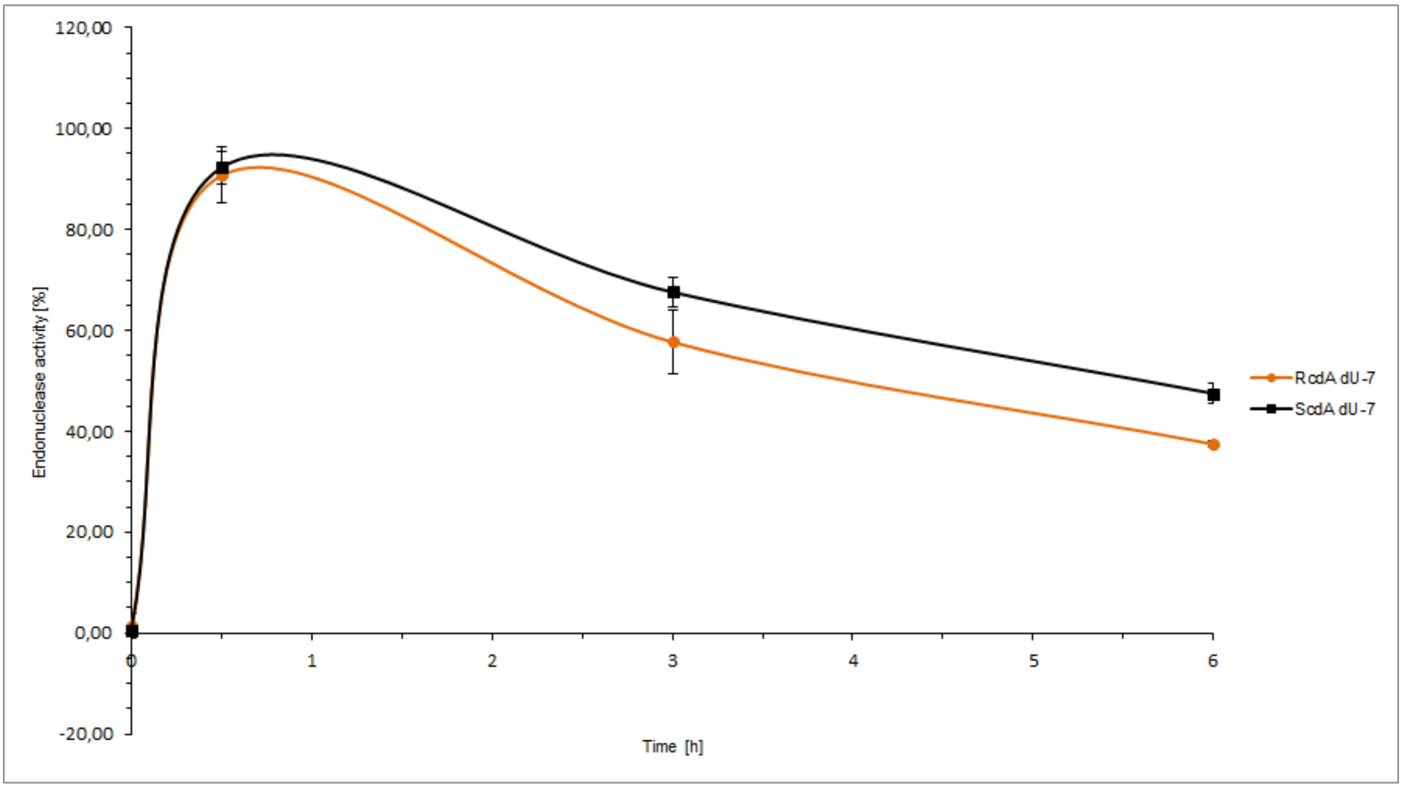

120,00
100,00
80,00
60,00
40,00
20,00
0,00
-20,00
Endonuclease activity [%]
0
1
2
3
4
5
6
Time [h]
RcdA dU-7
ScdA dU-7

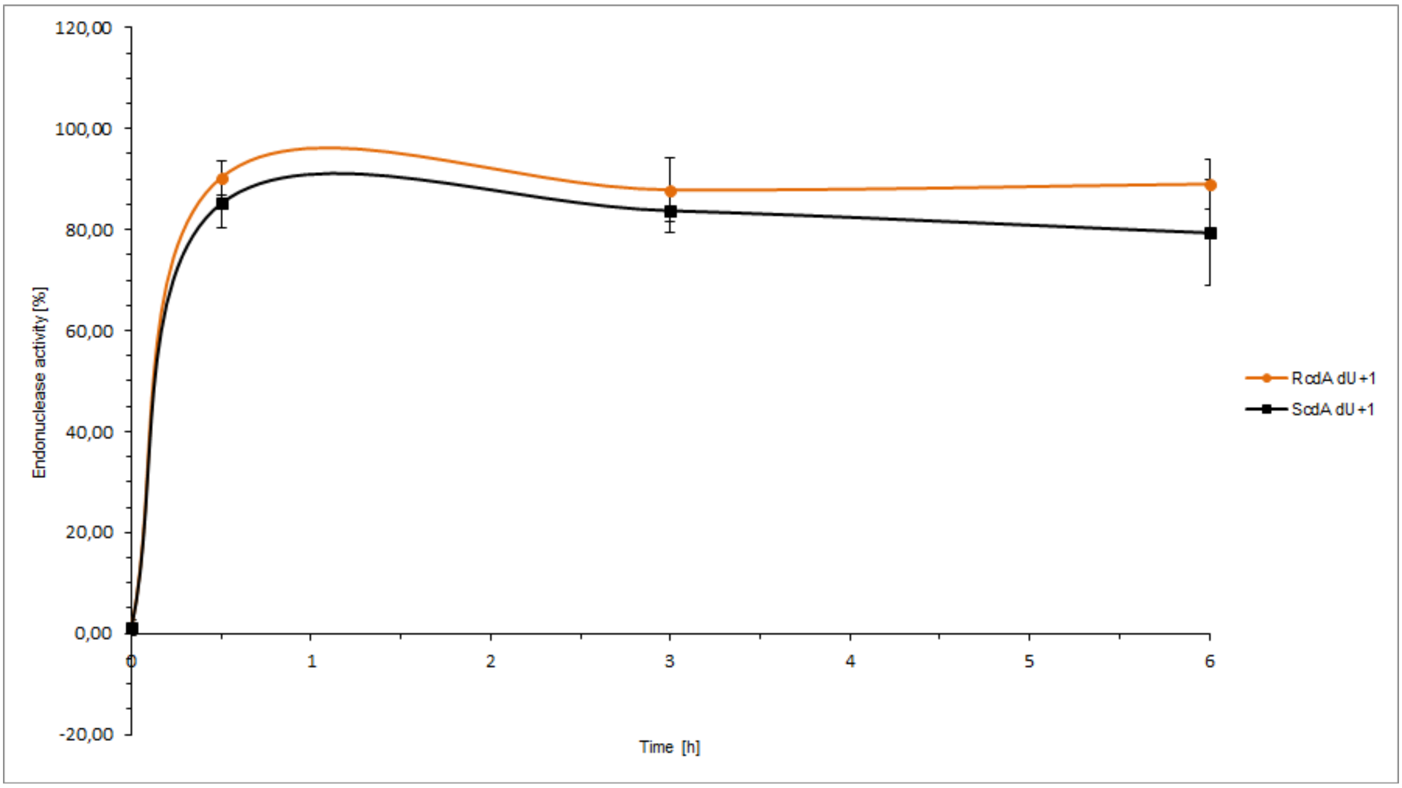

120,00
100,00
80,00
60,00
40,00
20,00
0,00
-20,00
Endonuclease activity [%]
0
1
2
3
4
5
6
Time [h]
RcdA dU+1
ScdA dU+1

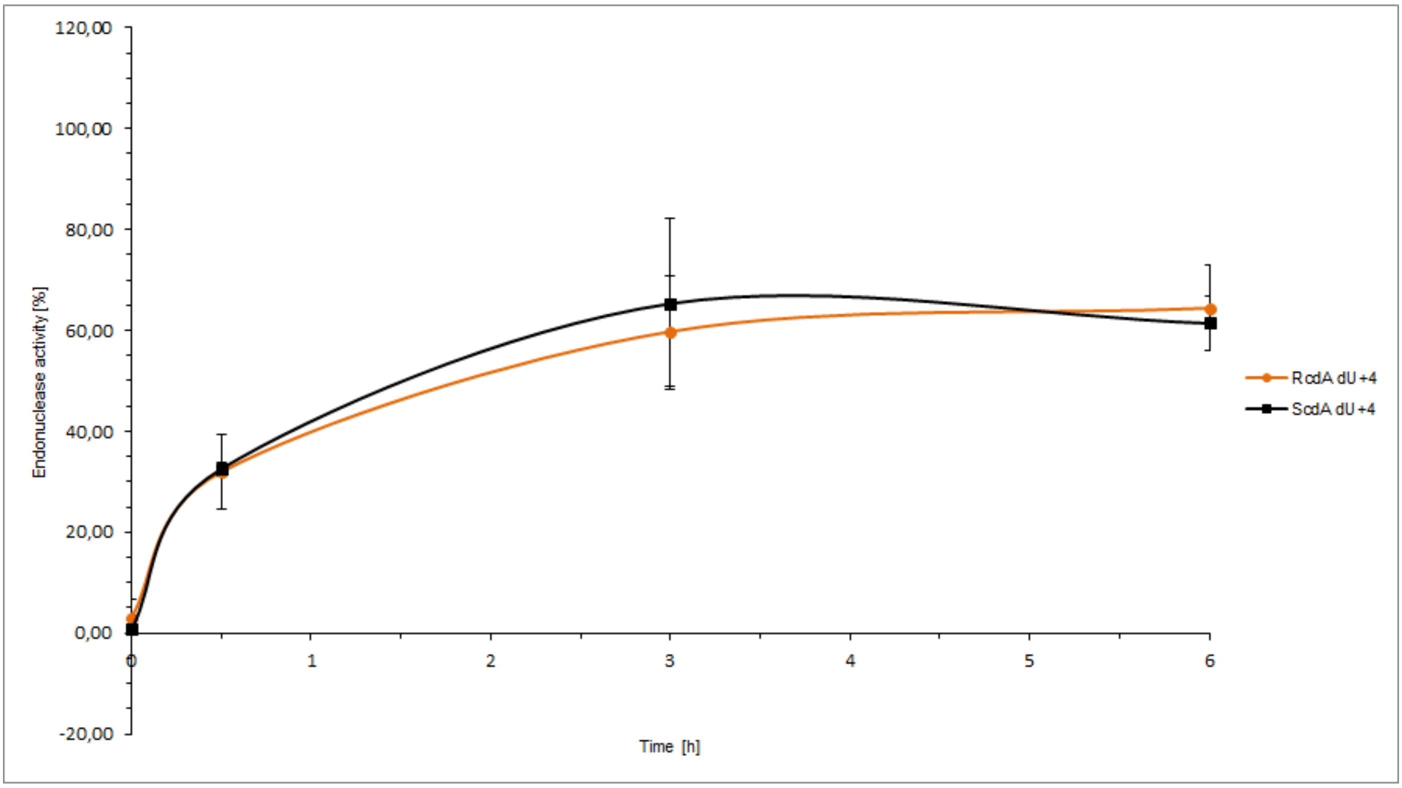
120,00
100,00
80,00
60,00
40,00
20,00
0,00
-20,00
Endonuclease activity [%]
0
1
2
3
4
5
6
Time [h]
RcdA dU+4
ScdA dU+4

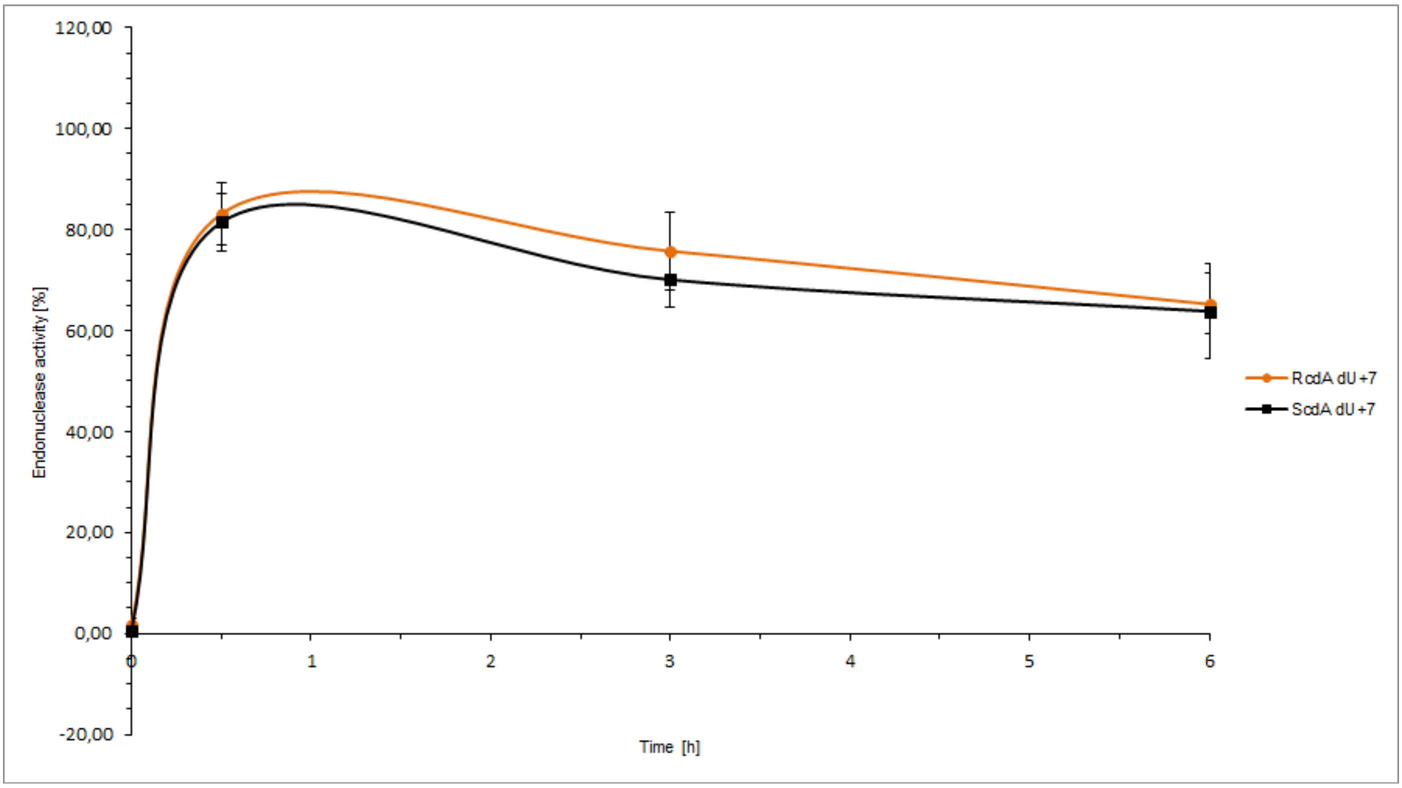
120,00
100,00
80,00
60,00
40,00
20,00
0,00
-20,00
Endonuclease activity [%]
0
1
2
3
4
5
6
Time [h]
RcdA dU+7
ScdA dU+7

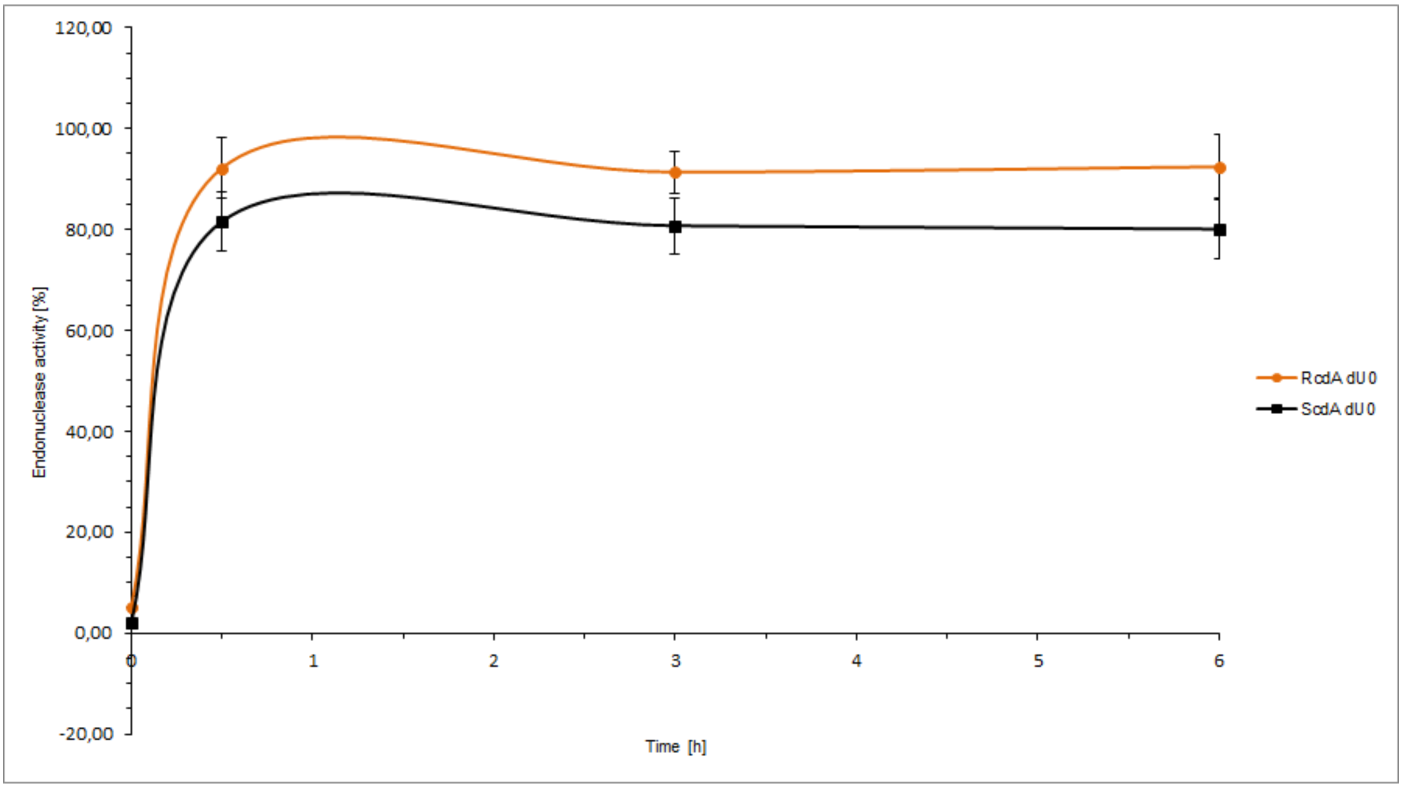

Figure S6. Endonuclease activity [%] of ScdA vs. RcdA – comparison of individual strands.

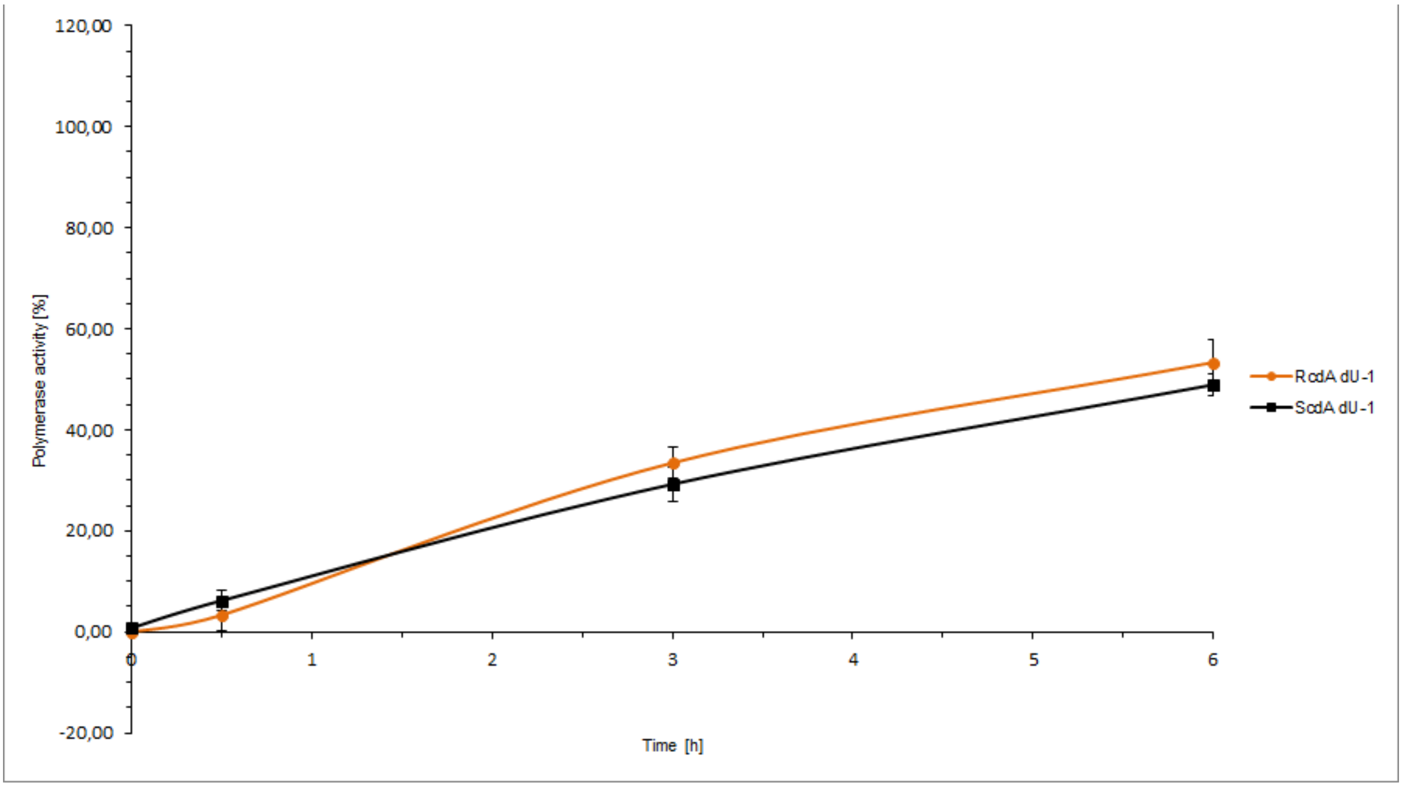
120,00
100,00
80,00
60,00
40,00
20,00
0,00
-20,00
Polymerase activity [%]
0
1
2
3
4
5
6
Time [h]
RcdA dU-1
ScdA dU-1

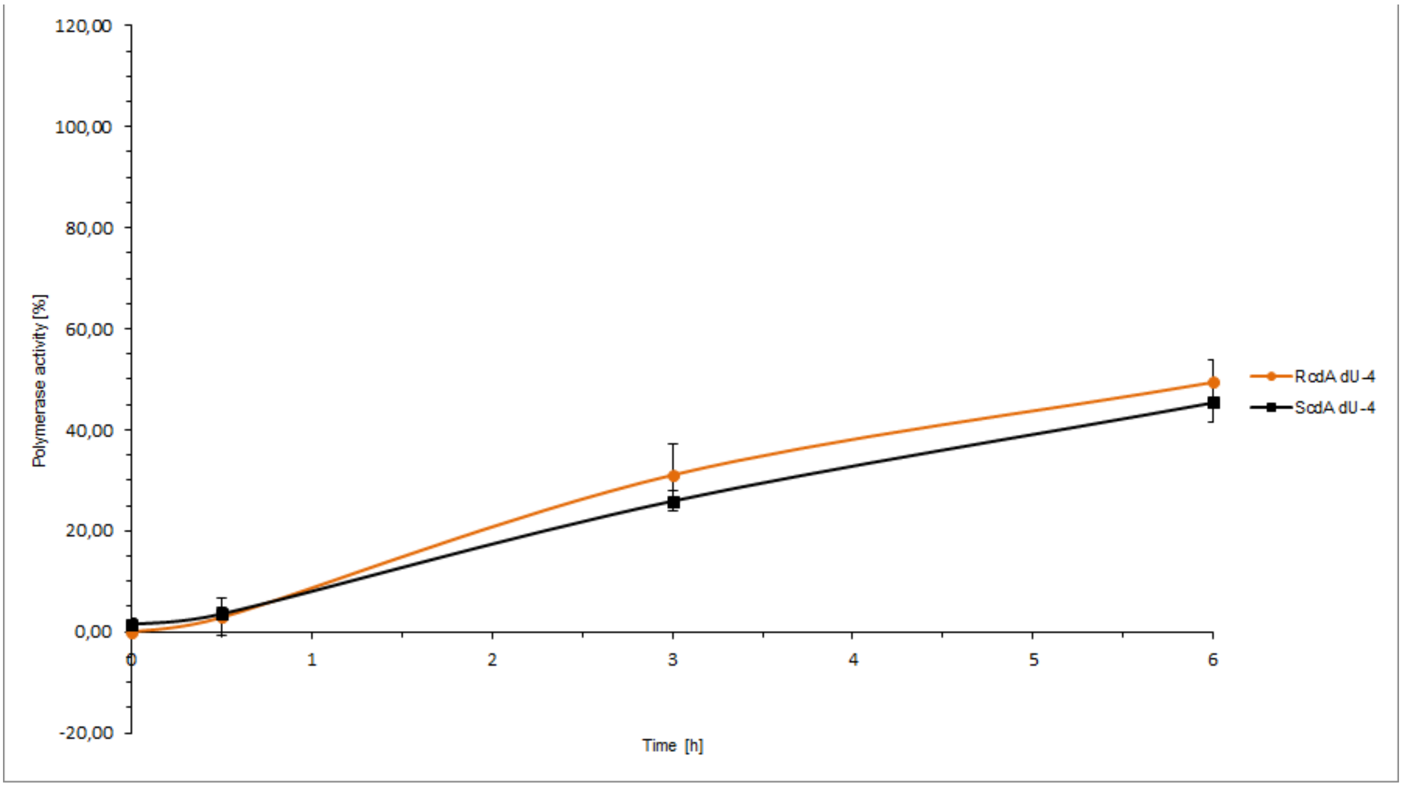
120,00
100,00
80,00
60,00
40,00
20,00
0,00
-20,00
Polymerase activity [%]
0
1
2
3
4
5
6
Time [h]
RcdA dU-4
ScdA dU-4

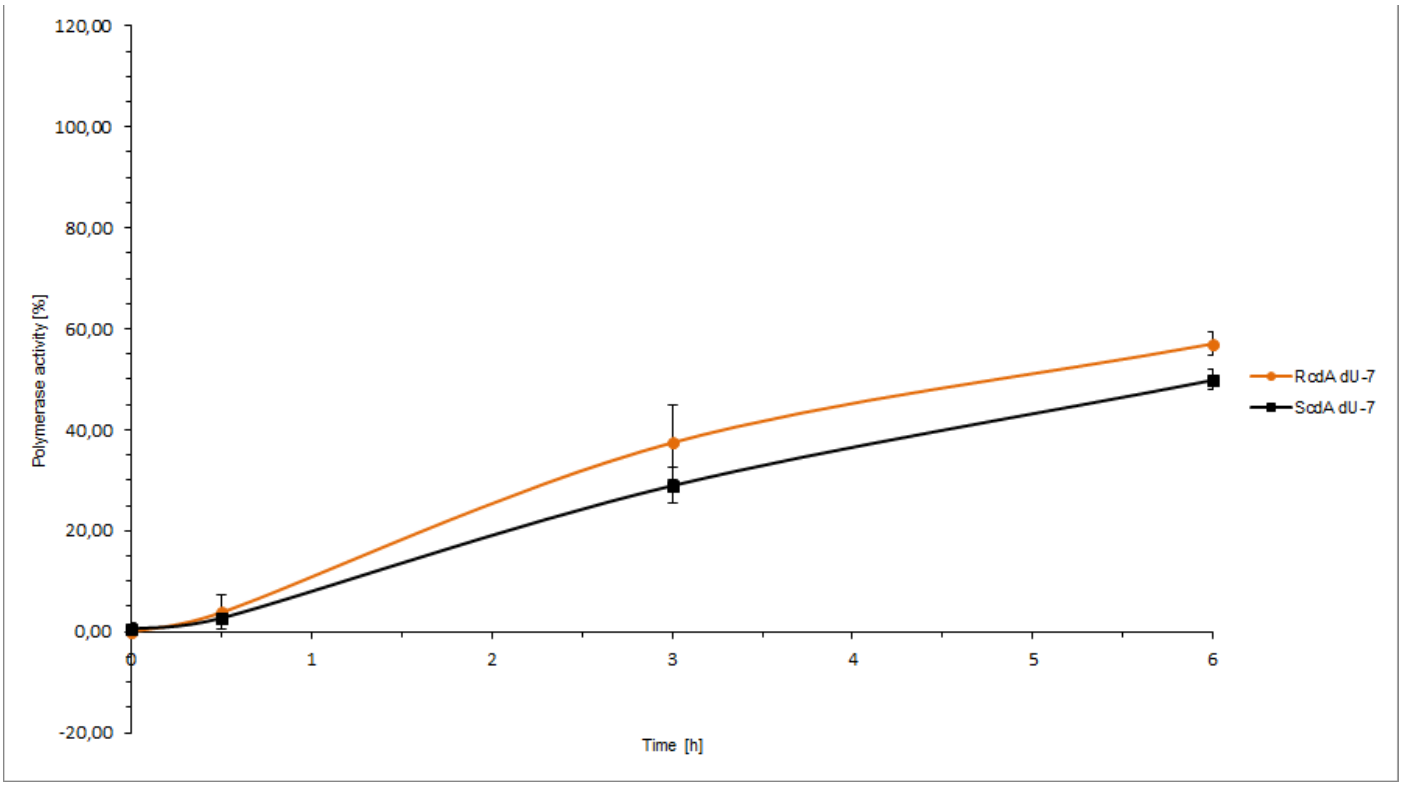
120,00
100,00
80,00
60,00
40,00
20,00
0,00
-20,00
Polymerase activity [%]
0
1
2
3
4
5
6
Time [h]
RcdA dU-7
ScdA dU-7

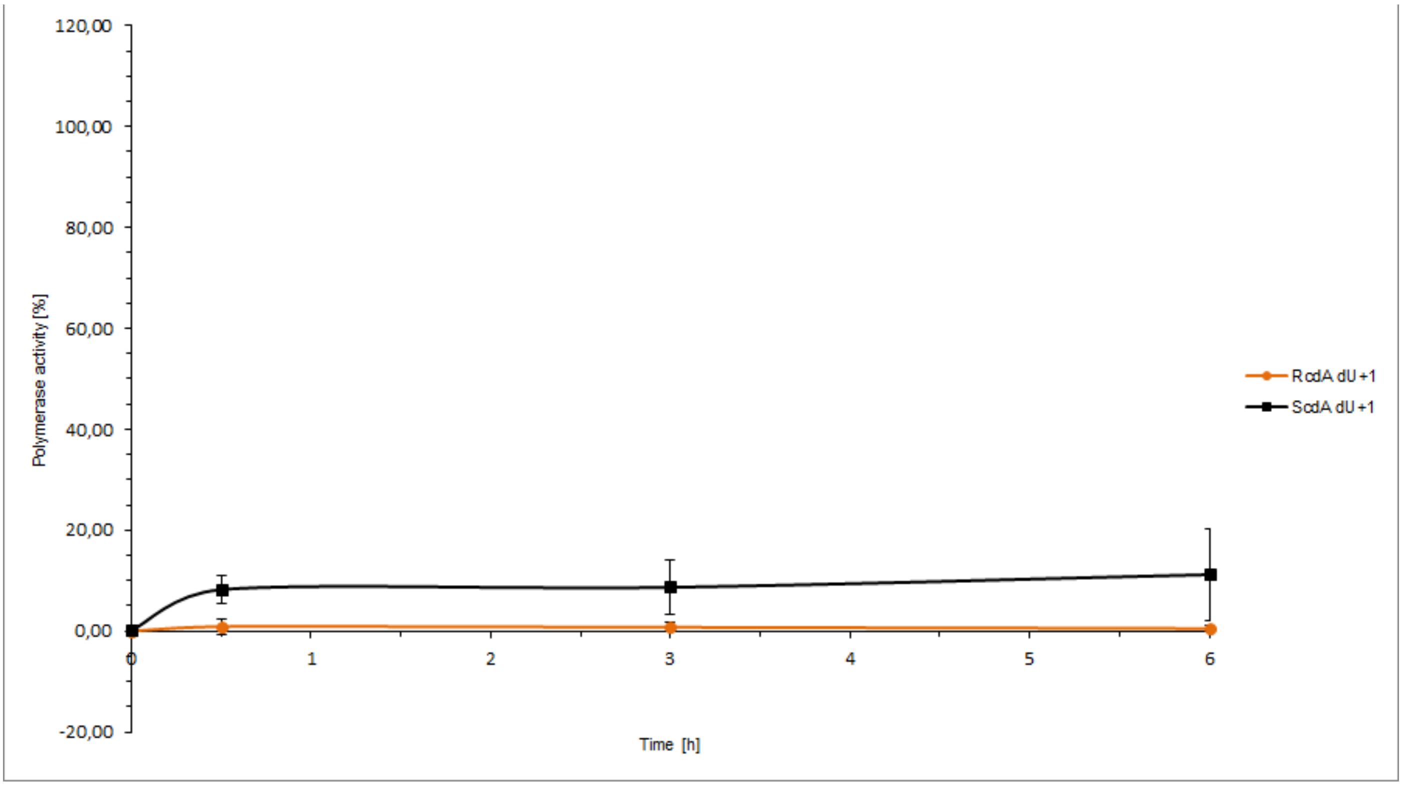
120,00
100,00
80,00
60,00
40,00
20,00
0,00
-20,00
Polymerase activity [%]
0
1
2
3
4
5
6
Time [h]
RcdA dU+1
ScdA dU+1

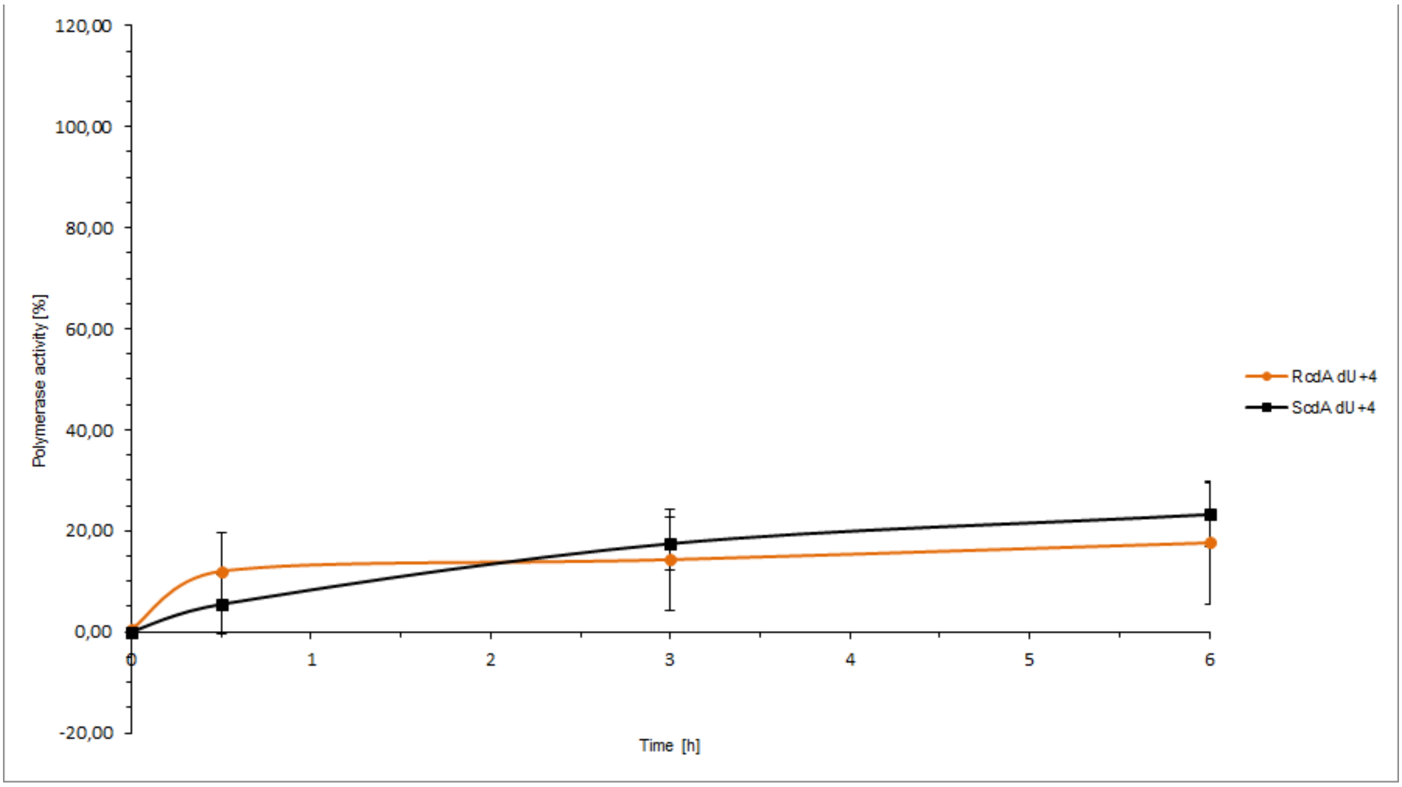
120,00
100,00
80,00
60,00
40,00
20,00
0,00
-20,00
Polymerase activity [%]
0
1
2
3
4
5
6
Time [h]
RcdA dU+4
ScdA dU+4

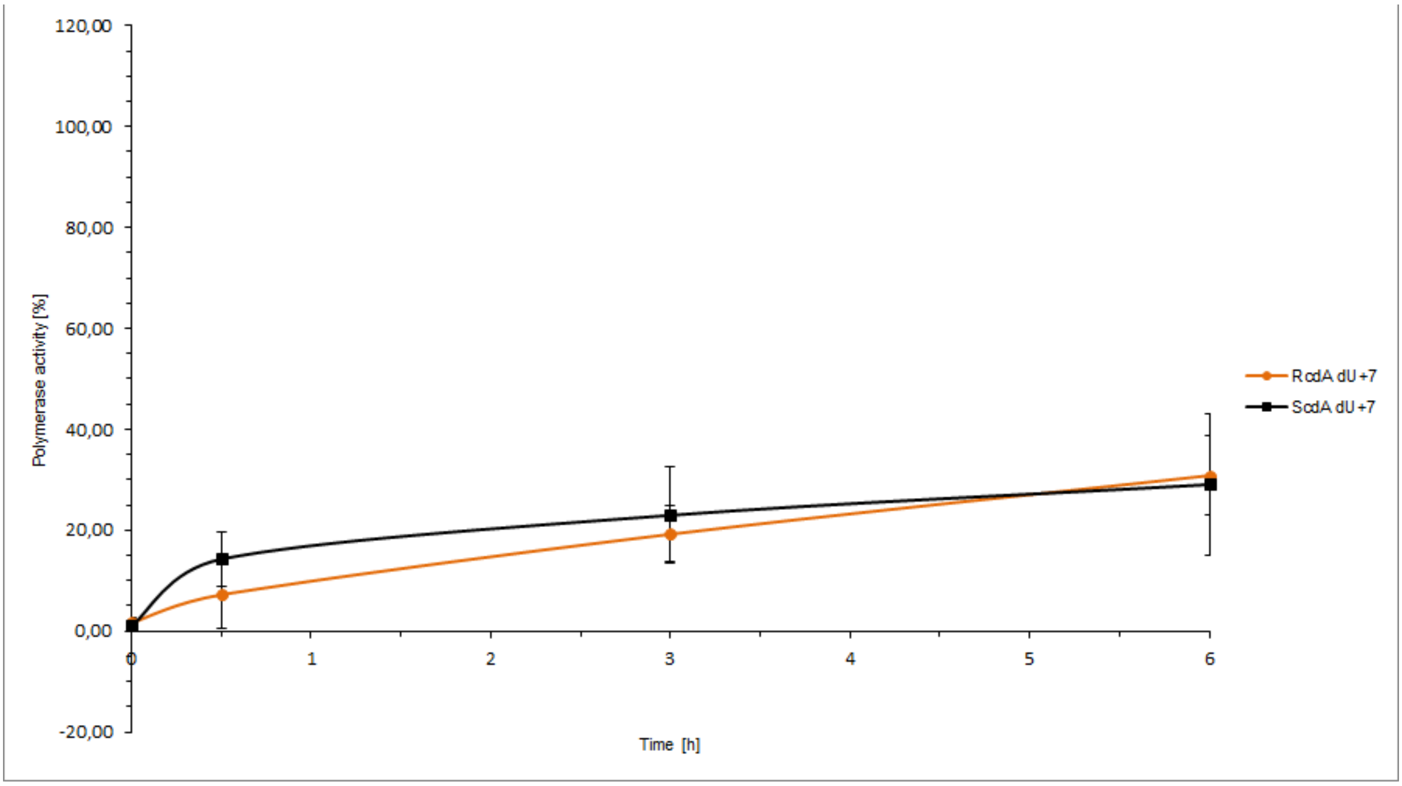
120,00
100,00
80,00
60,00
40,00
20,00
0,00
-20,00
Polymerase activity [%]
0
1
2
3
4
5
6
Time [h]
RcdA dU+7
ScdA dU+7

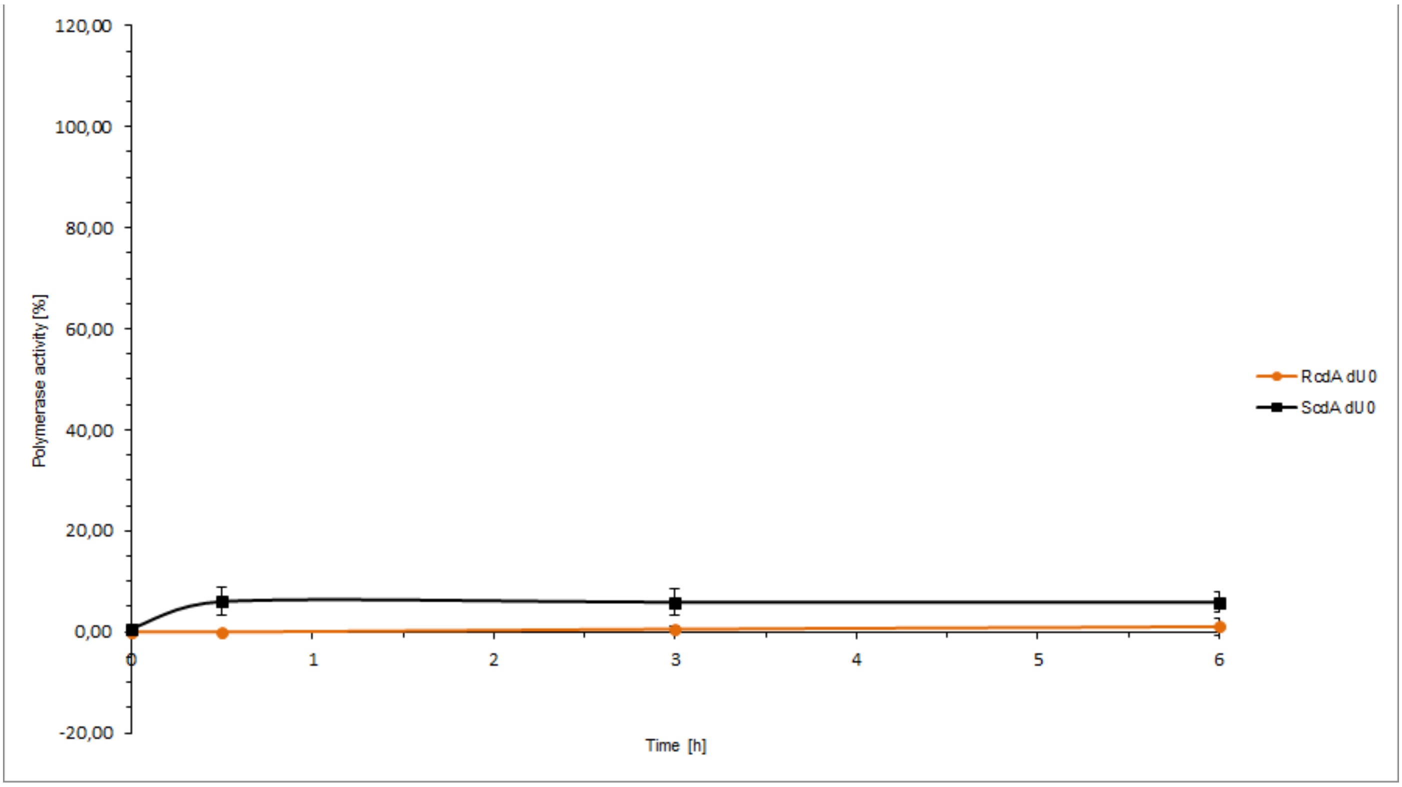

Figure S7. Polymerase activity [%] of ScdA vs. RcdA – comparison of individual strands.

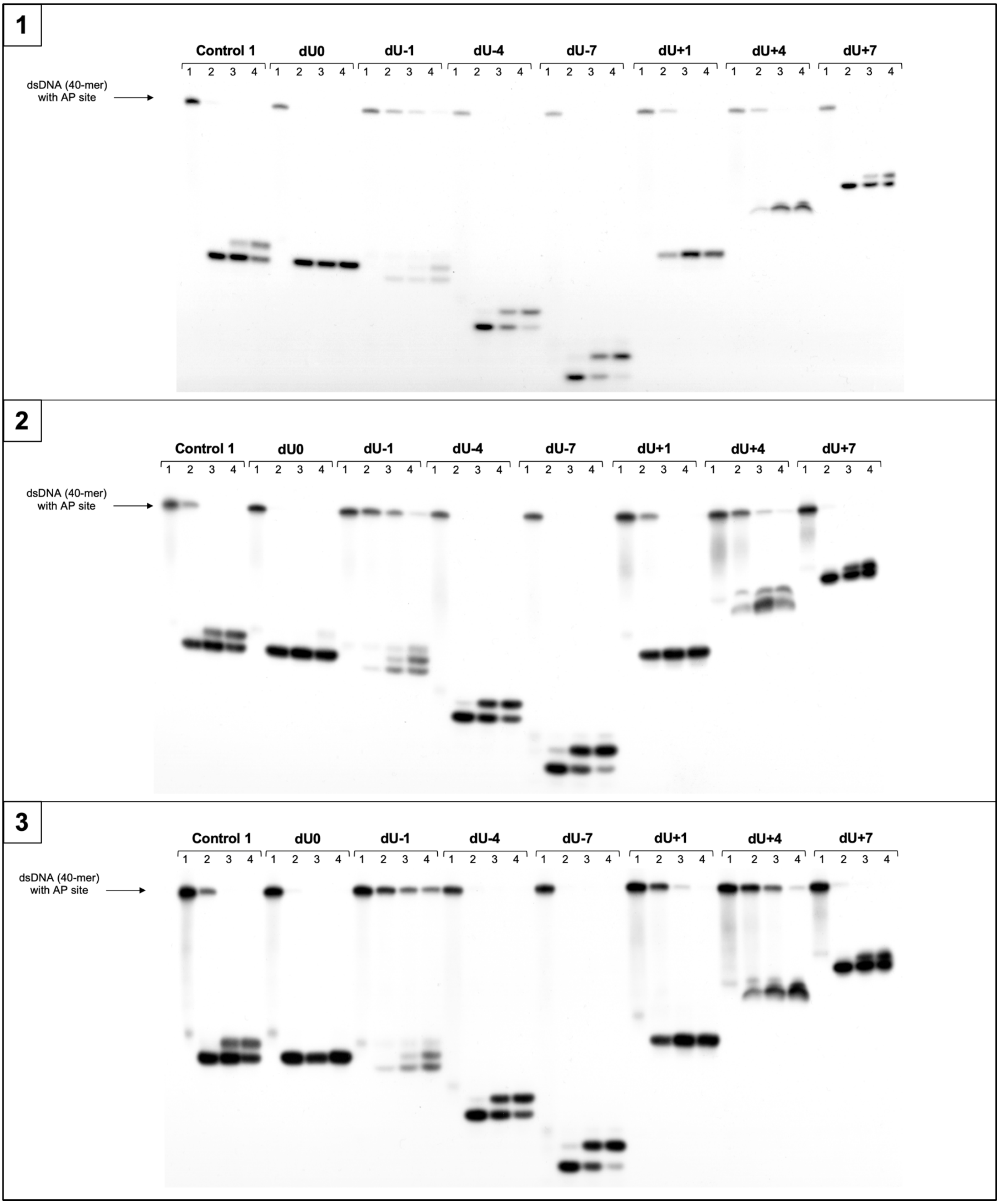

Figure S8. The autoradiograms of denaturing PAGE presenting repair of dsDNA containing clustered damage with AP site in one strand and **ScdG** in the opposing strand. Each lane number indicates different assay time: lane 1 - 0 min; lane 2 - 30 min; lane 3 – 3h; lane 4 – 6h. Three experimental replications are presented.

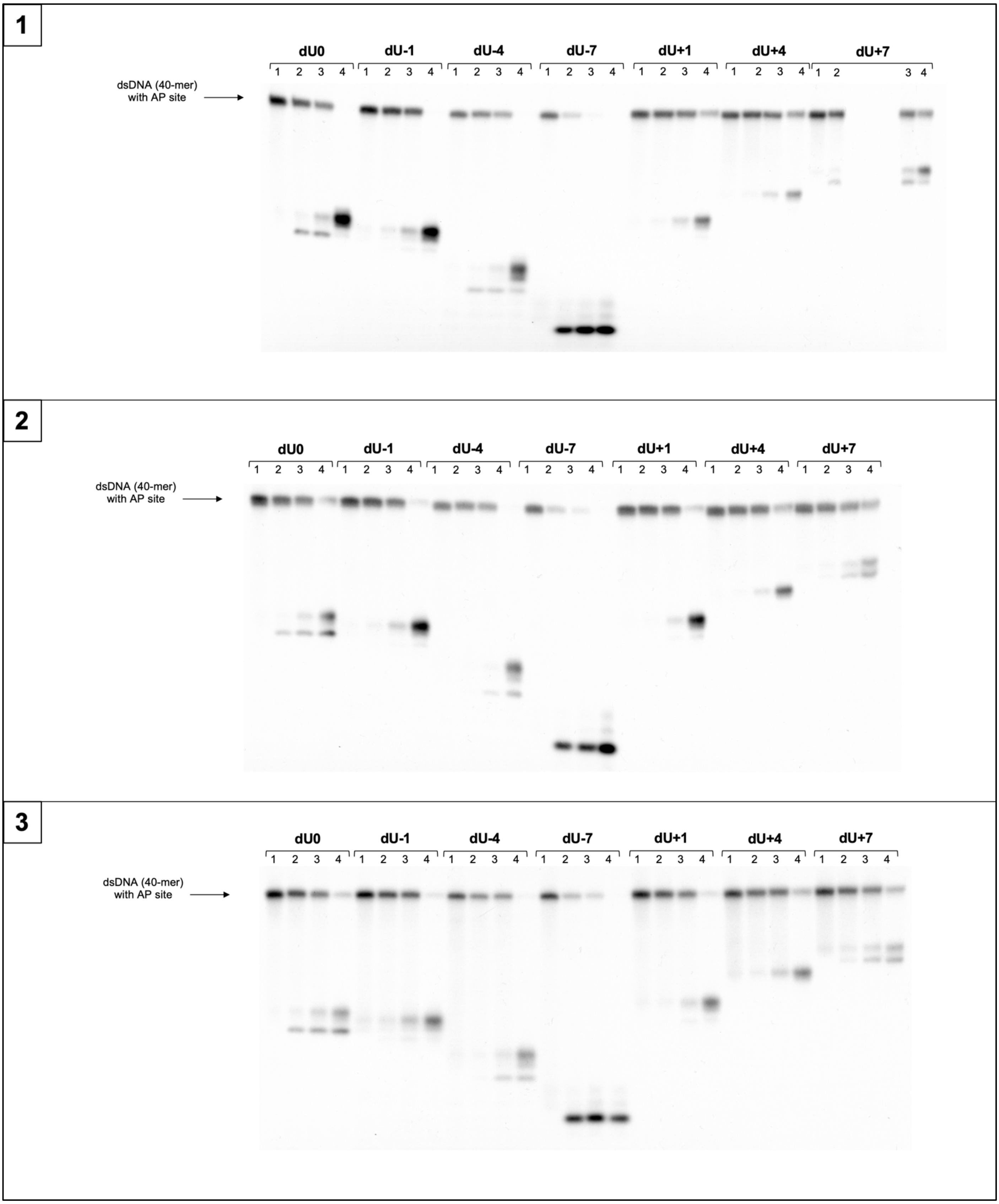

Figure S9. The autoradiograms of denaturing PAGE presenting repair of dsDNA containing clustered damage with AP site in one strand and **RcdG** in the opposing strand. Each lane number indicates different assay time: lane 1 - 0 min; lane 2 - 30 min; lane 3 – 3h; lane 4 – 6h. Three experimental replications are presented.

Table S6. Endonuclease activity – ScdG and RcdG. Raw numerical data of densitometry obtained from Quantity One software.

**RcdG**		Time [h]				**ScdG**		Time [h]			
		0	0,5	3	6			0	0,5	3	6
Strand	Data set	**Endonuclease activity [%]**				Strand	Data set	**Endonuclease activity [%]**			
dU0	1.	1,34	3,52	20,80	80,18	dU0	1.	0,92	99,54	99,53	94,41
	2.	0,15	11,18	19,91	34,40		2.	0,61	93,19	96,39	91,56
	3.	0,29	16,50	26,36	38,06		3.	0,00	99,82	99,91	99,87
	Avg	0,59	10,40	22,36	50,88		Avg	0,51	97,51	98,61	95,28
	SD	0,65	6,52	3,49	25,44		SD	0,47	3,75	1,93	4,22
dU-1	1.	0,00	6,45	24,32	74,24	dU-1	1.	0,00	8,07	38,34	33,08
	2.	2,20	5,31	23,15	82,50		2.	0,00	11,18	34,38	36,89
	3.	0,33	3,20	6,32	9,06		3.	1,15	31,47	27,36	25,02
	Avg	0,84	4,99	17,93	55,27		Avg	0,38	16,91	33,36	31,66
	SD	1,19	1,65	10,07	40,23		SD	0,66	12,71	5,56	6,06
dU-4	1.	0,00	0,00	7,57	63,04	dU-4	1.	0,00	89,74	52,47	38,80
	2.	0,55	0,97	6,30	78,72		2.	0,00	91,49	54,07	39,32
	3.	0,00	2,24	18,02	23,49		3.	1,24	57,67	48,13	44,72
	Avg	0,18	1,07	10,63	55,08		Avg	0,41	79,63	51,56	40,95
	SD	0,32	1,12	6,43	28,46		SD	0,71	19,04	3,07	3,28
dU-7	1.	2,53	73,82	81,68	77,08	dU-7	1.	0,28	79,48	39,88	18,78
	2.	0,00	82,62	93,85	87,47		2.	1,87	90,66	41,51	13,46
	3.	0,00	77,19	84,31	94,72		3.	0,72	52,53	39,74	28,23
	Avg	0,84	77,88	86,61	86,42		Avg	0,96	74,22	40,38	20,16
	SD	1,46	4,44	6,41	8,86		SD	0,82	19,60	0,98	7,48
dU+1	1.	1,43	3,17	18,68	58,64	dU+1	1.	0,00	69,95	86,05	85,67
	2.	0,60	1,19	13,39	82,12		2.	0,00	57,01	88,82	95,16
	3.	0,13	4,81	19,43	74,05		3.	0,49	47,27	58,87	69,80
	Avg	0,72	3,06	17,17	71,60		Avg	0,16	58,08	77,91	83,54
	SD	0,66	1,81	3,29	11,93		SD	0,28	11,38	16,55	12,81
dU+4	1.	1,19	2,64	13,43	45,41	dU+4	1.	1,07	27,69	66,03	56,93
	2.	0,89	1,77	10,91	66,59		2.	2,90	31,21	53,65	66,57
	3.	0,33	7,52	22,42	61,76		3.	2,24	37,85	42,39	43,19
	Avg	0,80	3,98	15,58	57,92		Avg	2,07	32,25	54,02	55,56
	SD	0,44	3,10	6,05	11,10		SD	0,93	5,16	11,83	11,75
dU+7	1.	0,45	10,43	19,35	13,91	dU+7	1.	2,27	86,81	63,04	54,57
	2.	1,27	2,84	16,54	30,64		2.	0,21	84,48	66,20	60,46
	3.	1,47	5,89	19,34	32,14		3.	1,08	53,61	52,08	53,08
	Avg	1,06	6,39	18,41	25,56		Avg	1,18	74,96	60,44	56,04
	SD	0,54	3,82	1,62	10,12		SD	1,03	18,53	7,41	3,90

Table S7. Polymerase activity – ScdG and RcdG. Raw numerical data of densitometry obtained from Quantity One software.

**RcdG**		Time [h]				**ScdG**		Time [h]			
		0	0,5	3	6			0	0,5	3	6
Strand	Data set	**Polymerase activity [%]**				Strand	Data set	**Polymerase activity [%]**			
dU0	1.	0,54	0,47	0,71	12,18	dU0	1.	0,00	0,11	0,39	5,48
	2.	1,13	2,33	12,25	47,64		2.	0,00	5,08	3,60	6,88
	3.	2,08	5,22	17,95	44,40		3.	0,00	0,00	0,00	0,00
	Avg	1,25	2,67	10,30	34,74		Avg	0,00	1,73	1,33	4,12
	SD	0,78	2,39	8,78	19,60		SD	0,00	2,90	1,97	3,64
dU-1	1.	0,00	0,00	0,00	2,69	dU-1	1.	1,41	5,04	28,71	61,81
	2.	0,62	0,71	1,09	3,69		2.	1,94	1,16	21,72	39,25
	3.	1,15	11,76	29,72	81,46		3.	0,00	8,99	23,41	51,96
	Avg	0,59	4,16	10,27	29,28		Avg	1,11	5,06	24,61	51,01
	SD	0,58	6,60	16,86	45,19		SD	1,00	3,92	3,64	11,31
dU-4	1.	0,00	0,00	0,00	0,60	dU-4	1.	0,00	9,70	47,47	61,13
	2.	0,00	0,00	0,00	0,13		2.	0,00	6,09	45,34	60,05
	3.	0,00	5,34	17,32	72,03		3.	0,00	30,25	47,09	52,01
	Avg	0,00	1,78	5,77	24,25		Avg	0,00	15,35	46,64	57,73
	SD	0,00	3,08	10,00	41,37		SD	0,00	13,03	1,13	4,98
dU-7	1.	0,00	9,40	12,05	18,83	dU-7	1.	2,50	17,11	58,97	81,01
	2.	0,00	0,00	0,36	11,33		2.	0,34	6,89	57,65	85,37
	3.	1,36	0,83	4,27	4,20		3.	8,89	27,44	54,82	67,03
	Avg	0,45	3,41	5,56	11,45		Avg	3,91	17,15	57,15	77,80
	SD	0,78	5,20	5,95	7,32		SD	4,45	10,28	2,12	9,58
dU+1	1.	0,33	0,31	0,84	5,83	dU+1	1.	0,81	2,73	2,16	2,07
	2.	0,25	0,20	0,42	2,48		2.	0,00	1,29	3,45	0,33
	3.	2,22	1,69	2,09	4,62		3.	0,00	4,89	8,57	5,15
	Avg	0,93	0,73	1,12	4,31		Avg	0,27	2,97	4,73	2,51
	SD	1,11	0,83	0,87	1,70		SD	0,47	1,81	3,39	2,44
dU+4	1.	1,07	0,77	0,90	1,07	dU+4	1.	0,00	14,91	24,25	37,13
	2.	1,03	0,97	0,77	1,87		2.	0,05	14,51	18,46	27,86
	3.	0,44	4,30	5,06	4,35		3.	0,00	25,93	36,99	38,93
	Avg	0,85	2,01	2,24	2,43		Avg	0,02	18,45	26,57	34,64
	SD	0,35	1,98	2,44	1,71		SD	0,03	6,48	9,48	5,94
dU+7	1.	2,72	3,97	15,31	47,85	dU+7	1.	0,00	11,11	36,85	45,40
	2.	1,64	3,18	10,41	36,39		2.	0,04	10,19	32,92	39,18
	3.	1,35	9,93	15,28	33,45		3.	1,93	16,69	30,97	33,33
	Avg	1,90	5,69	13,66	39,23		Avg	0,66	12,66	33,58	39,30
	SD	0,72	3,69	2,82	7,61		SD	1,10	3,52	3,00	6,03

A.

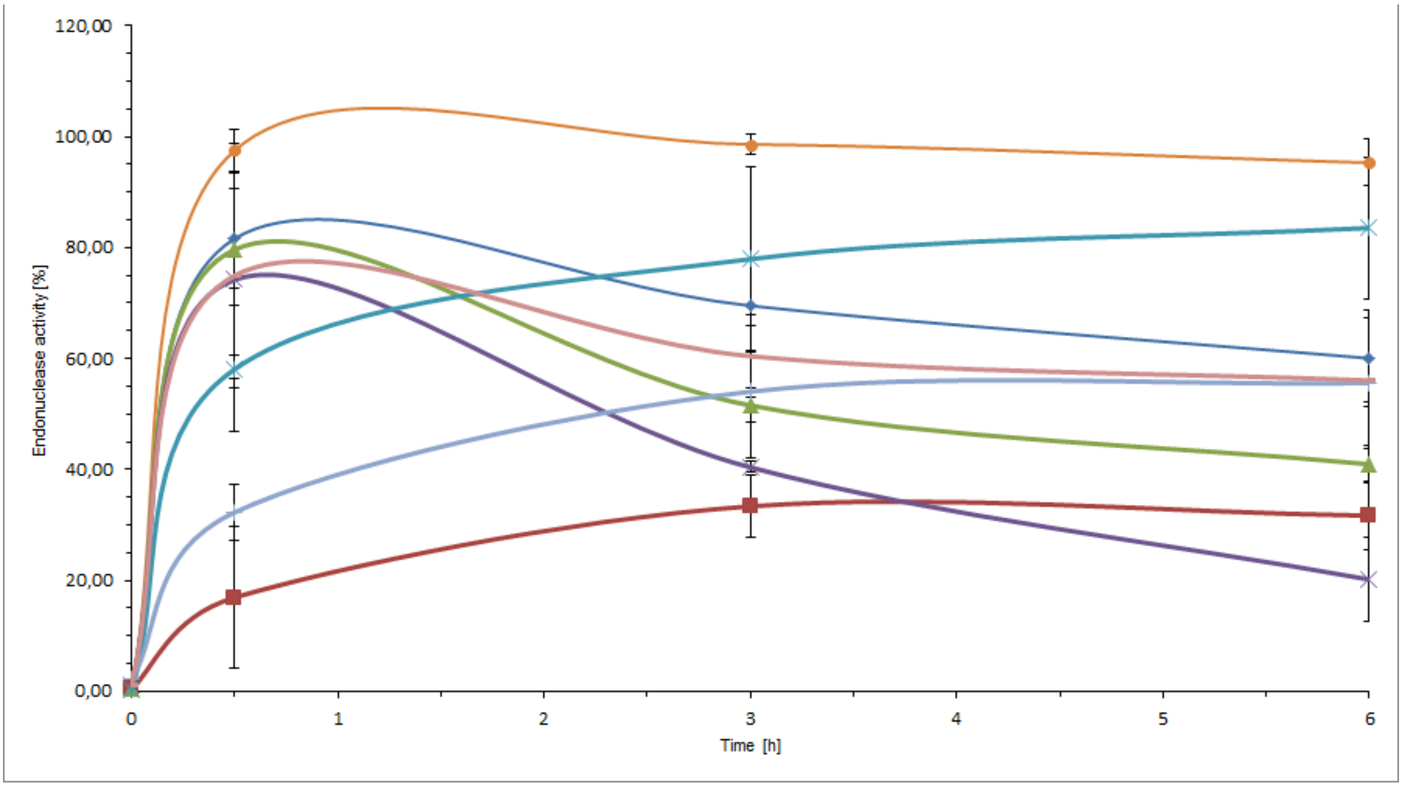

120,00
100,00
80,00
60,00
40,00
20,00
0,00
Endonuclease activity [%]
0
1
2
3
4
5
6
Time [h]

B.

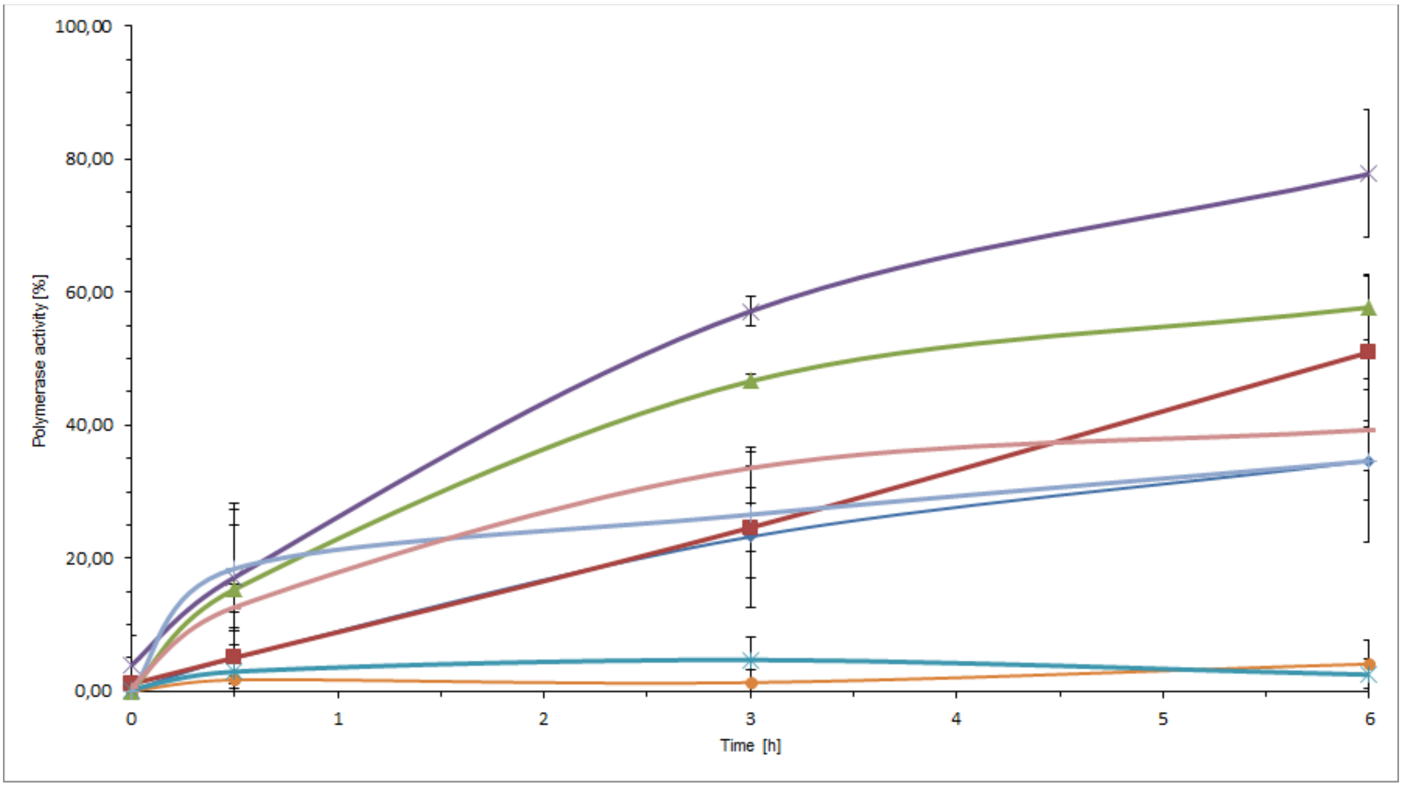

100,00
80,00
60,00
40,00
20,00
0,00
Polymerase activity [%]
0
1
2
3
4
5
6
Time [h]

C.

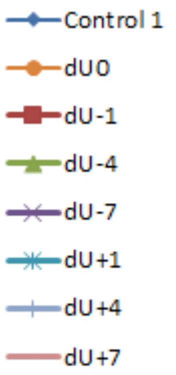

Figure S10. Graphical representation of the results for ScdG. (A) endonuclease activity +SD, (B) polymerase activity + SD; (C) figure legend.

A.

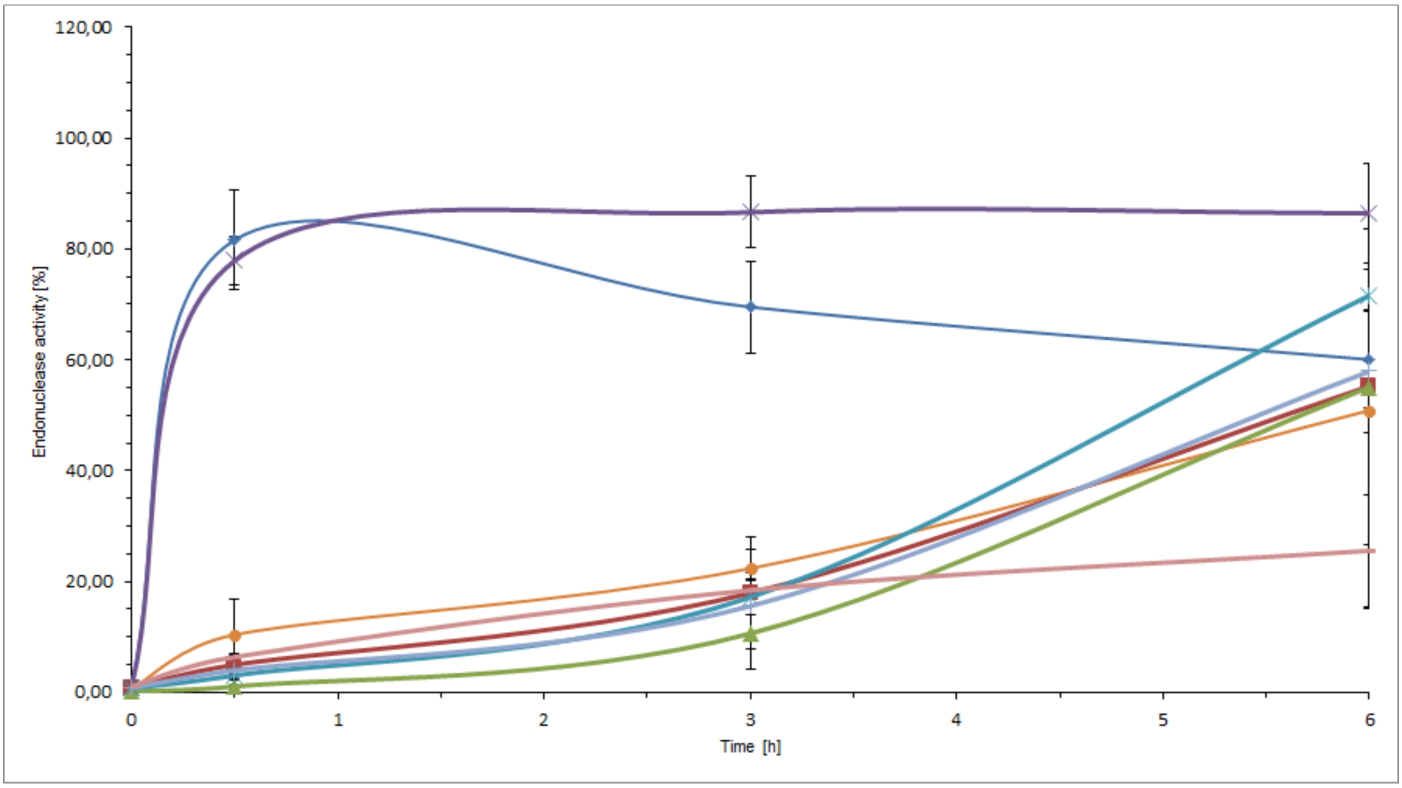

B.

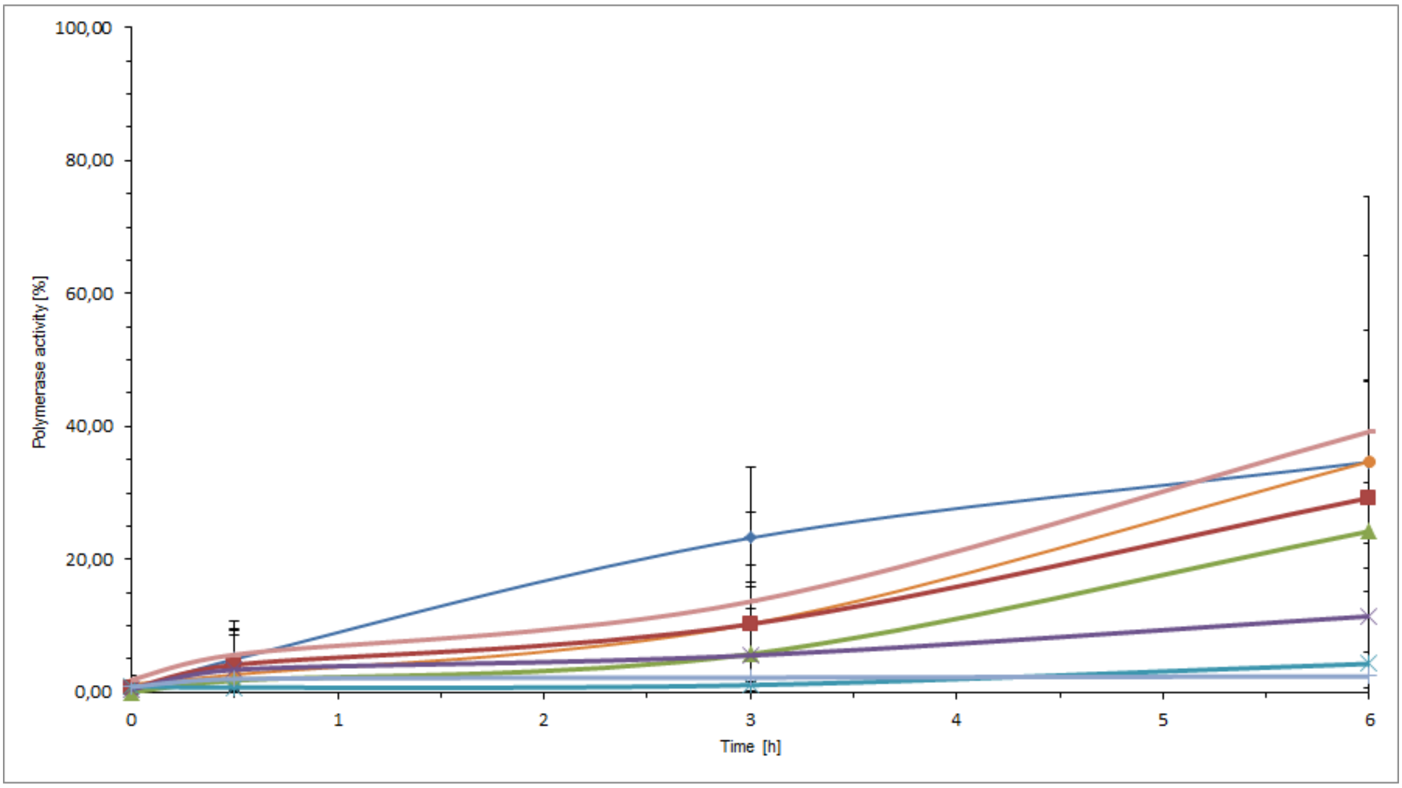

C.

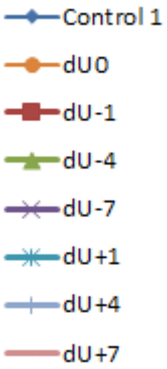

Figure S11. Graphical representation of the results for RcdG. (A) endonuclease activity +SD, (B) polymerase activity + SD; (C) figure legend.

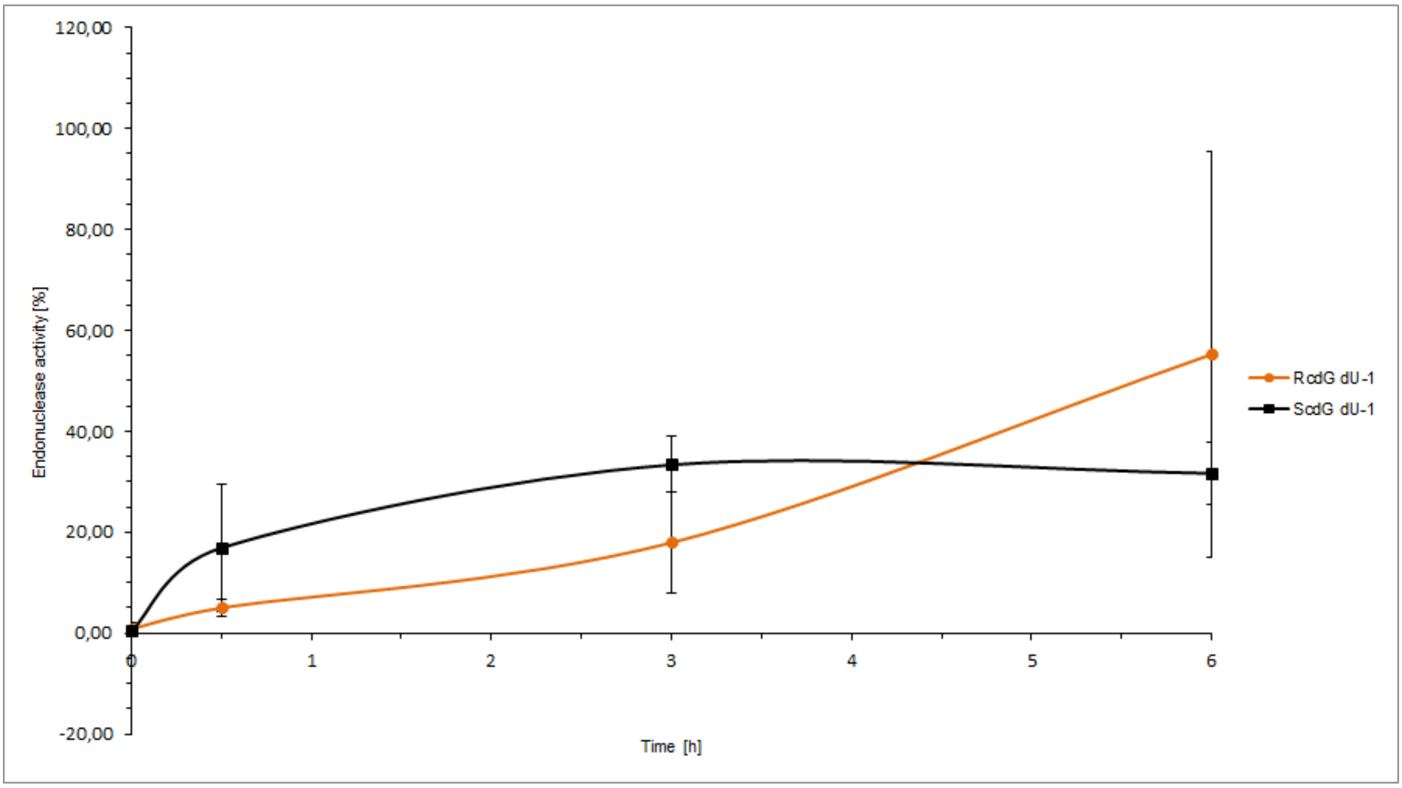
120,00
100,00
80,00
60,00
40,00
20,00
0,00
-20,00
Endonuclease activity [%]
0
1
2
3
4
5
6
Time [h]
RcdG dU-1
ScdG dU-1

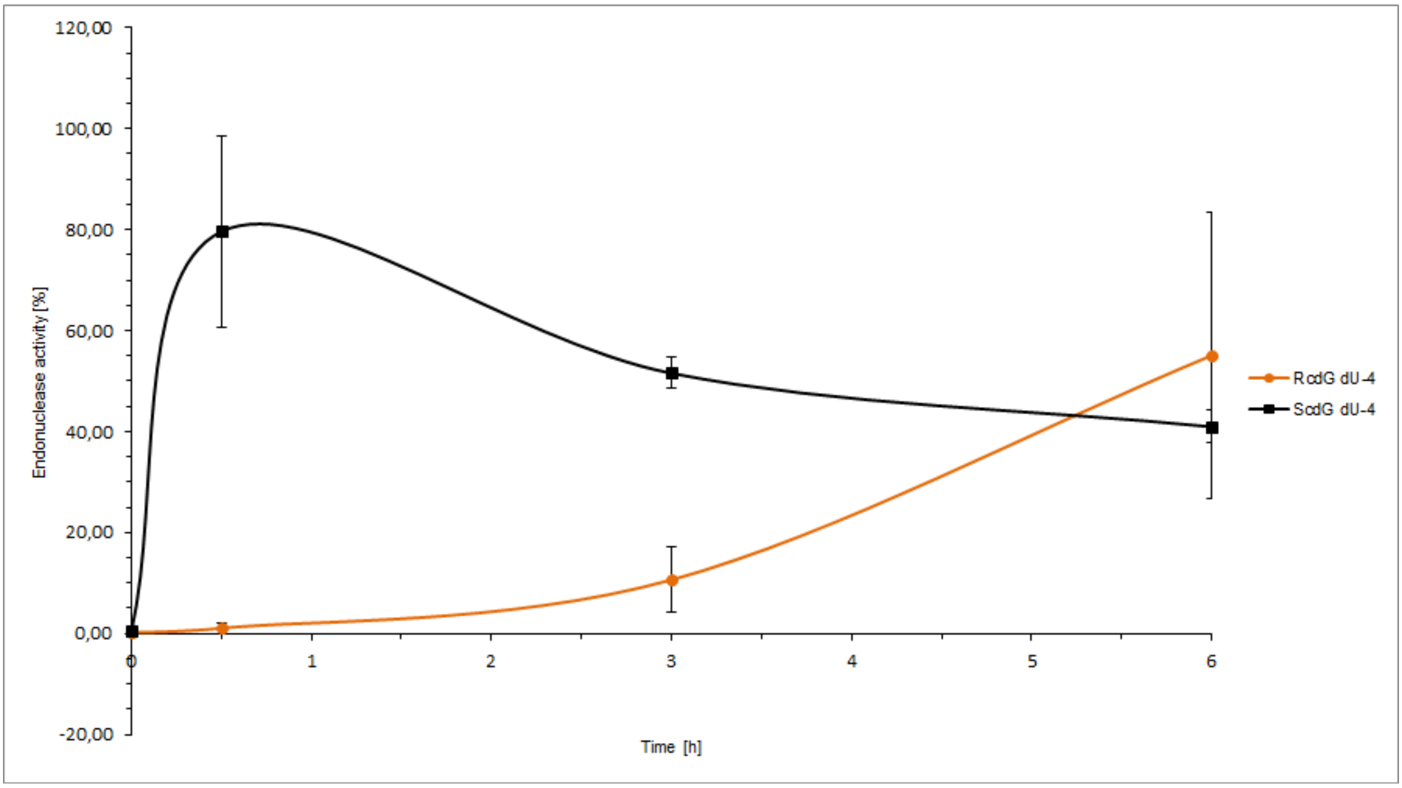
120,00
100,00
80,00
60,00
40,00
20,00
0,00
-20,00
Endonuclease activity [%]
0
1
2
3
4
5
6
Time [h]
RcdG dU-4
ScdG dU-4

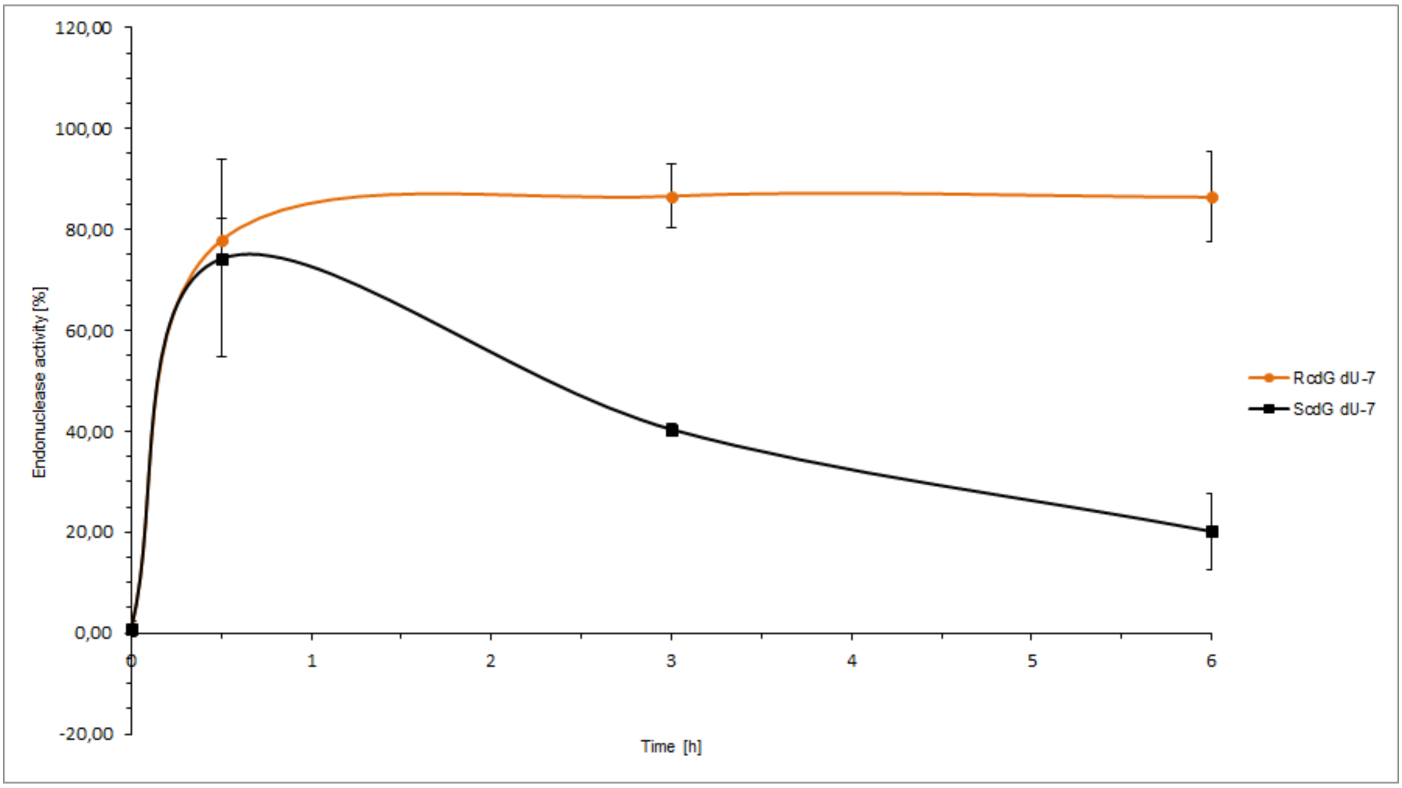
120,00
100,00
80,00
60,00
40,00
20,00
0,00
-20,00
Endonuclease activity [%]
0
1
2
3
4
5
6
Time [h]
RcdG dU-7
ScdG dU-7

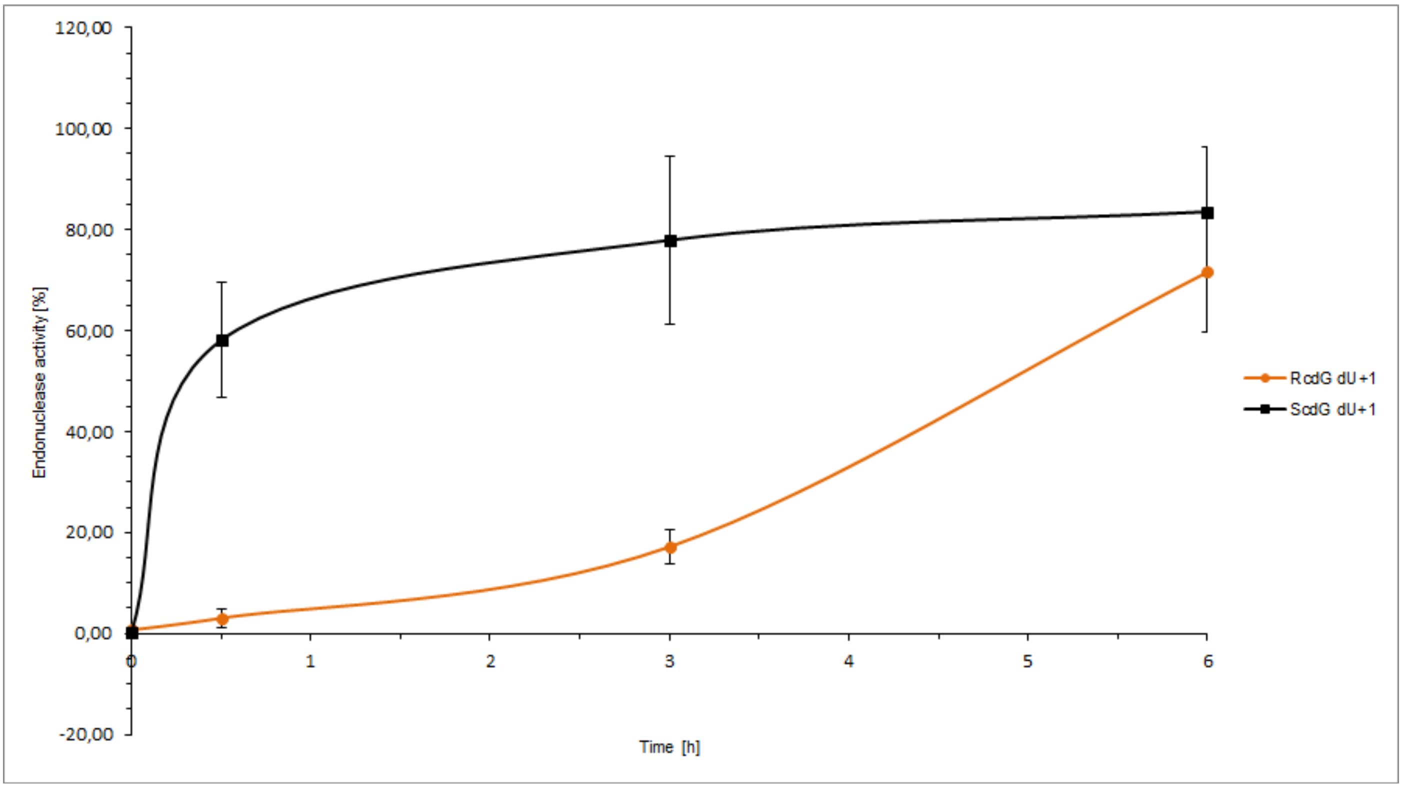
120,00
100,00
80,00
60,00
40,00
20,00
0,00
-20,00
Endonuclease activity [%]
0
1
2
3
4
5
6
Time [h]
RcdG dU+1
ScdG dU+1

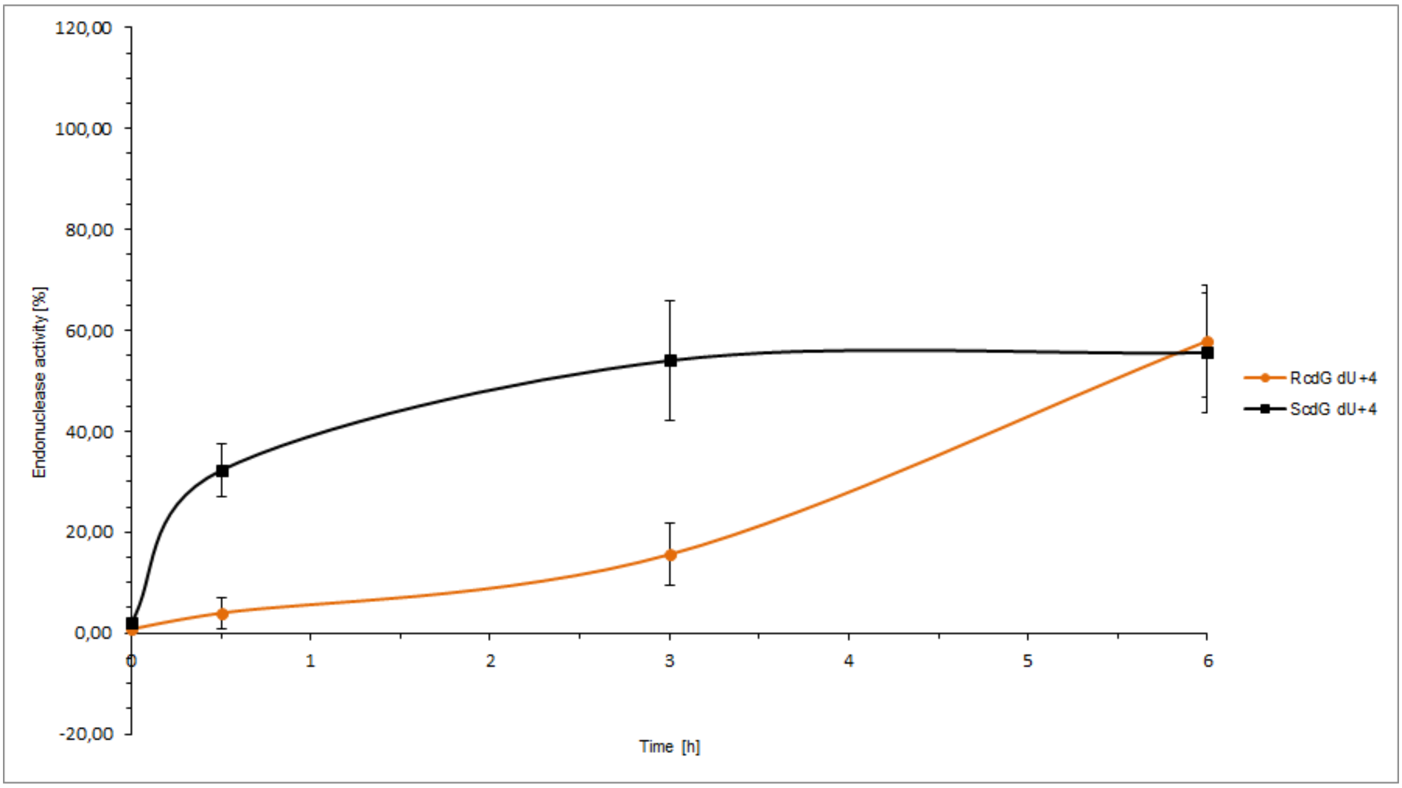

120,00
100,00
80,00
60,00
40,00
20,00
0,00
-20,00
Endonuclease activity [%]
0
1
2
3
4
5
6
Time [h]
RcdG dU+4
ScdG dU+4

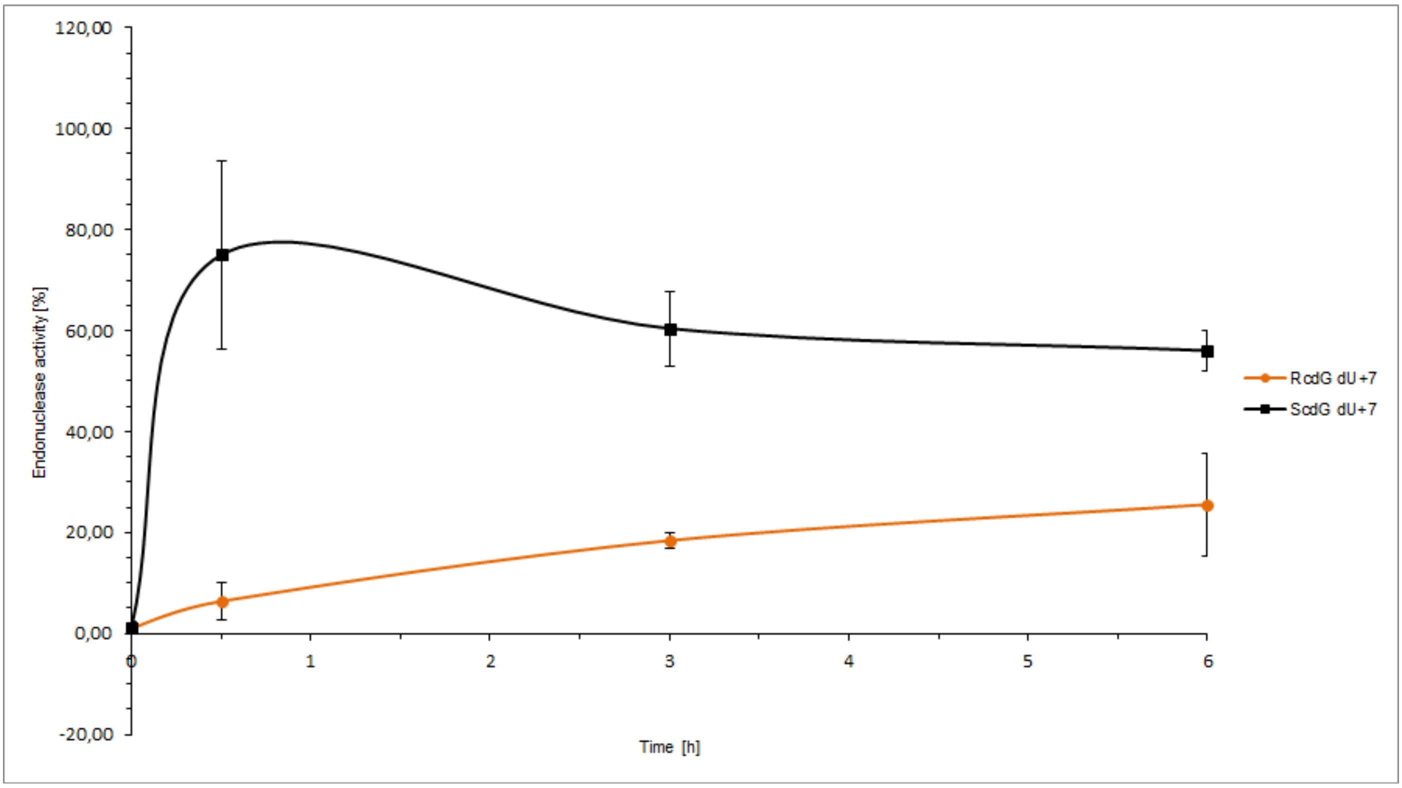

120,00
100,00
80,00
60,00
40,00
20,00
0,00
-20,00
Endonuclease activity [%]
0
1
2
3
4
5
6
Time [h]
RcdG dU+7
ScdG dU+7

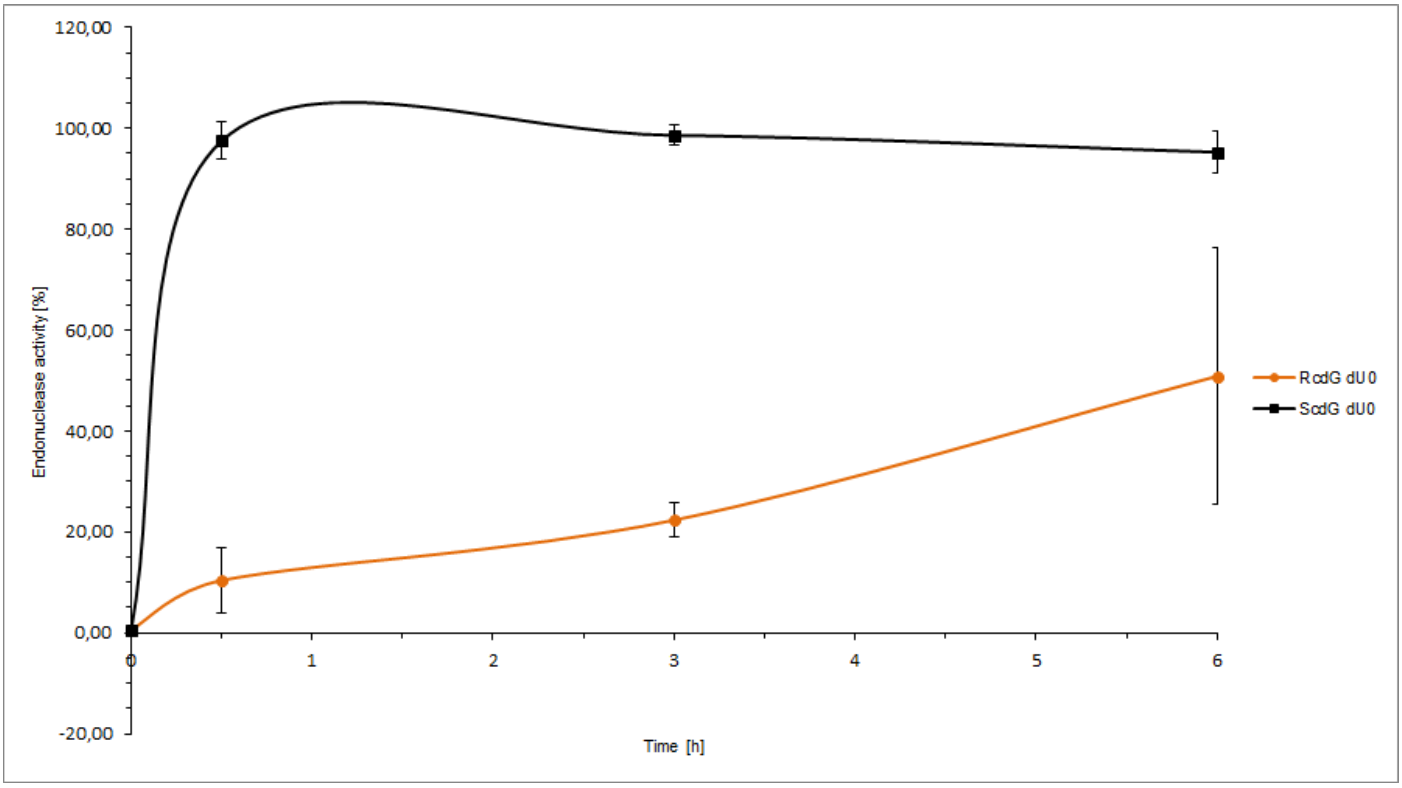

Figure S12. Endonuclease activity [%] of ScdG vs. RcdG – comparison of individual strands.

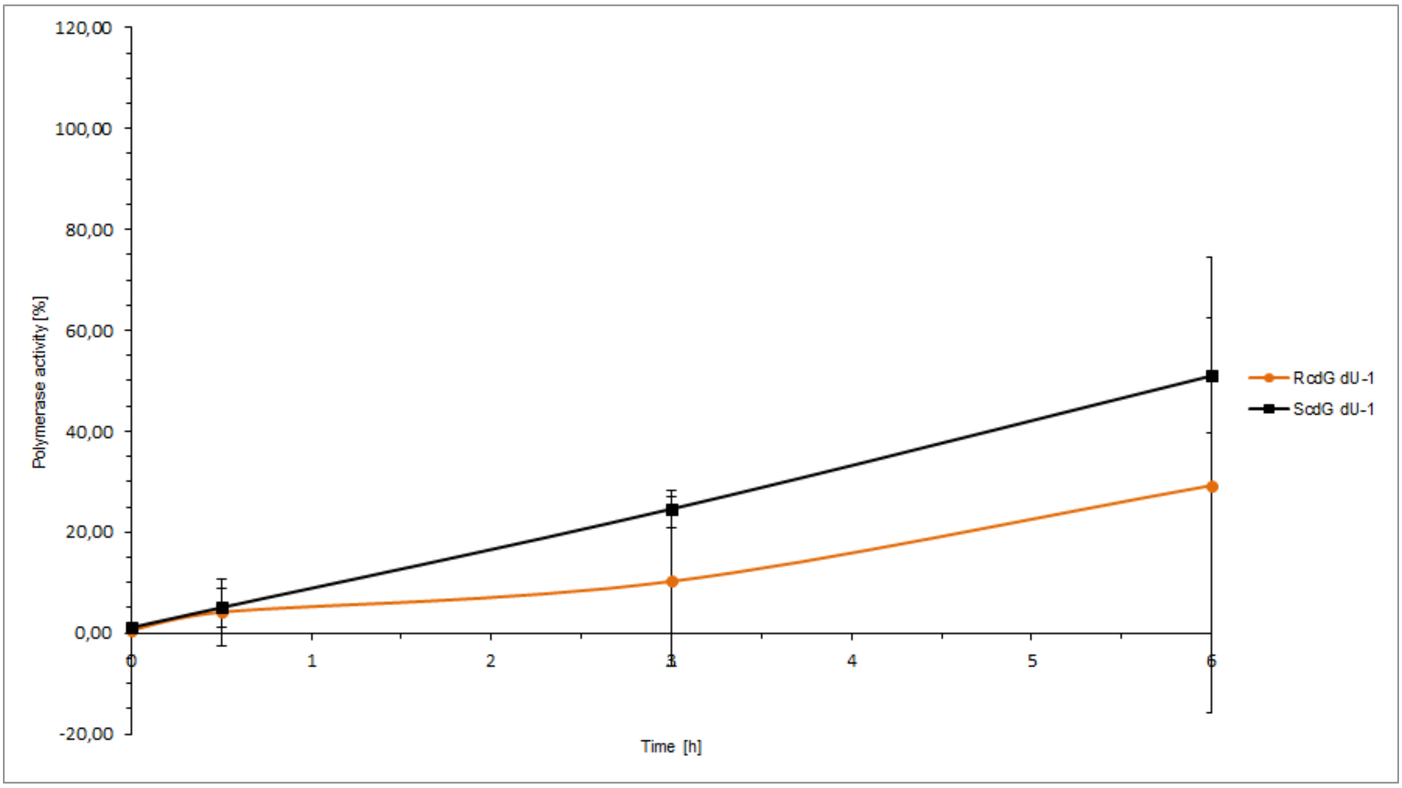

120,00
100,00
80,00
60,00
40,00
20,00
0,00
-20,00
Polymerase activity [%]
0
1
2
3
4
5
6
Time [h]
RcdG dU-1
ScdG dU-1

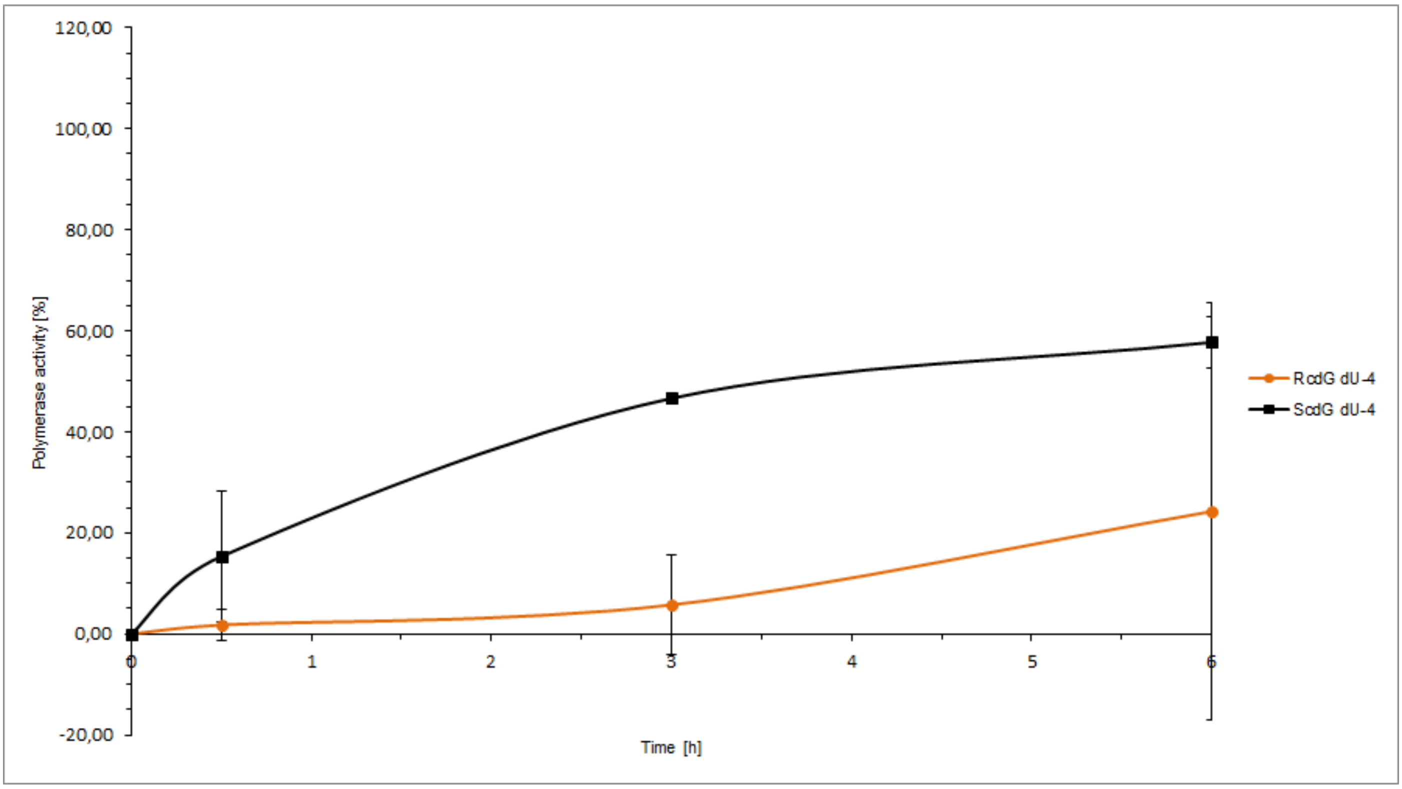

120,00
100,00
80,00
60,00
40,00
20,00
0,00
-20,00
Polymerase activity [%]
0
1
2
3
4
5
6
Time [h]
RcdG dU-4
ScdG dU-4

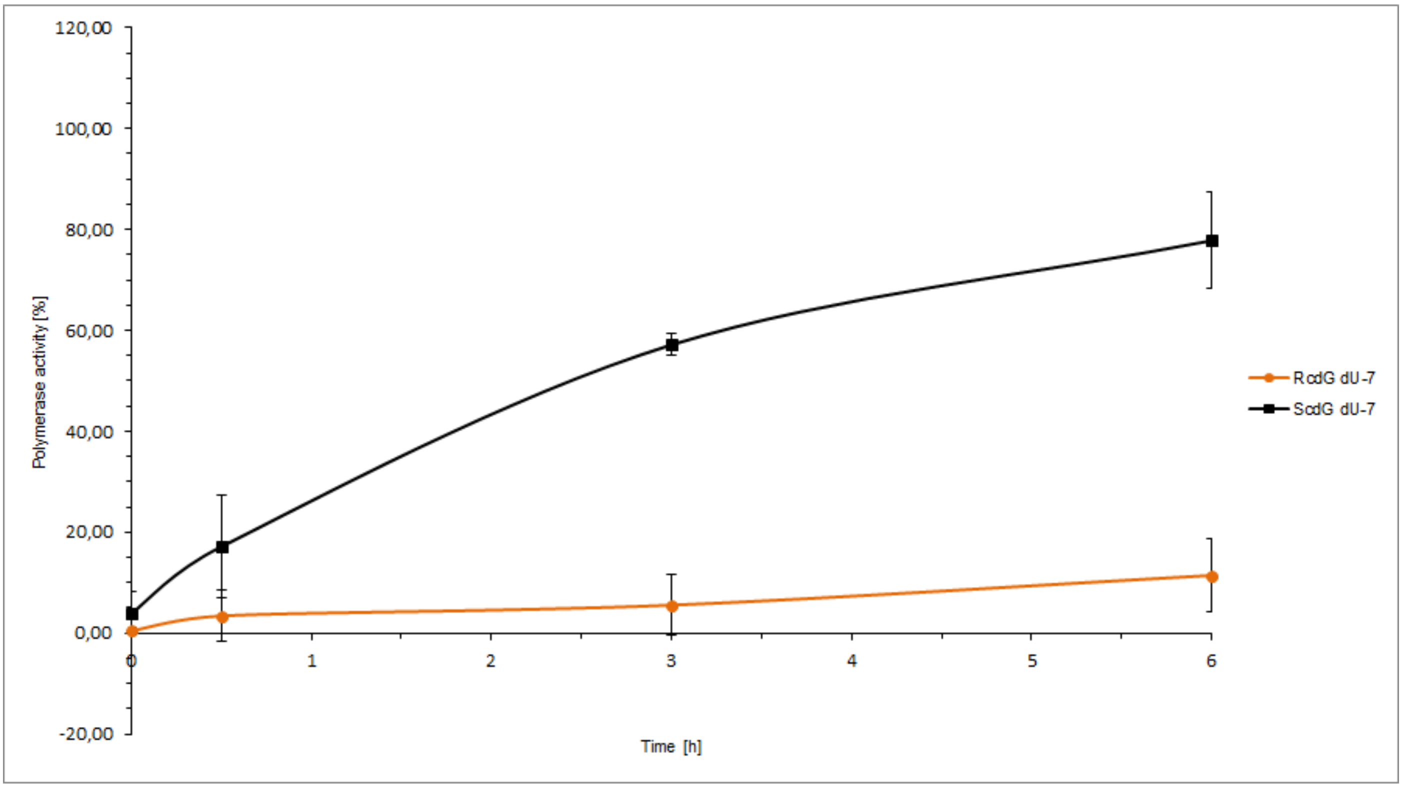
120,00
100,00
80,00
60,00
40,00
20,00
0,00
-20,00
Polymerase activity [%]
0
1
2
3
4
5
6
Time [h]
RcdG dU-7
ScdG dU-7

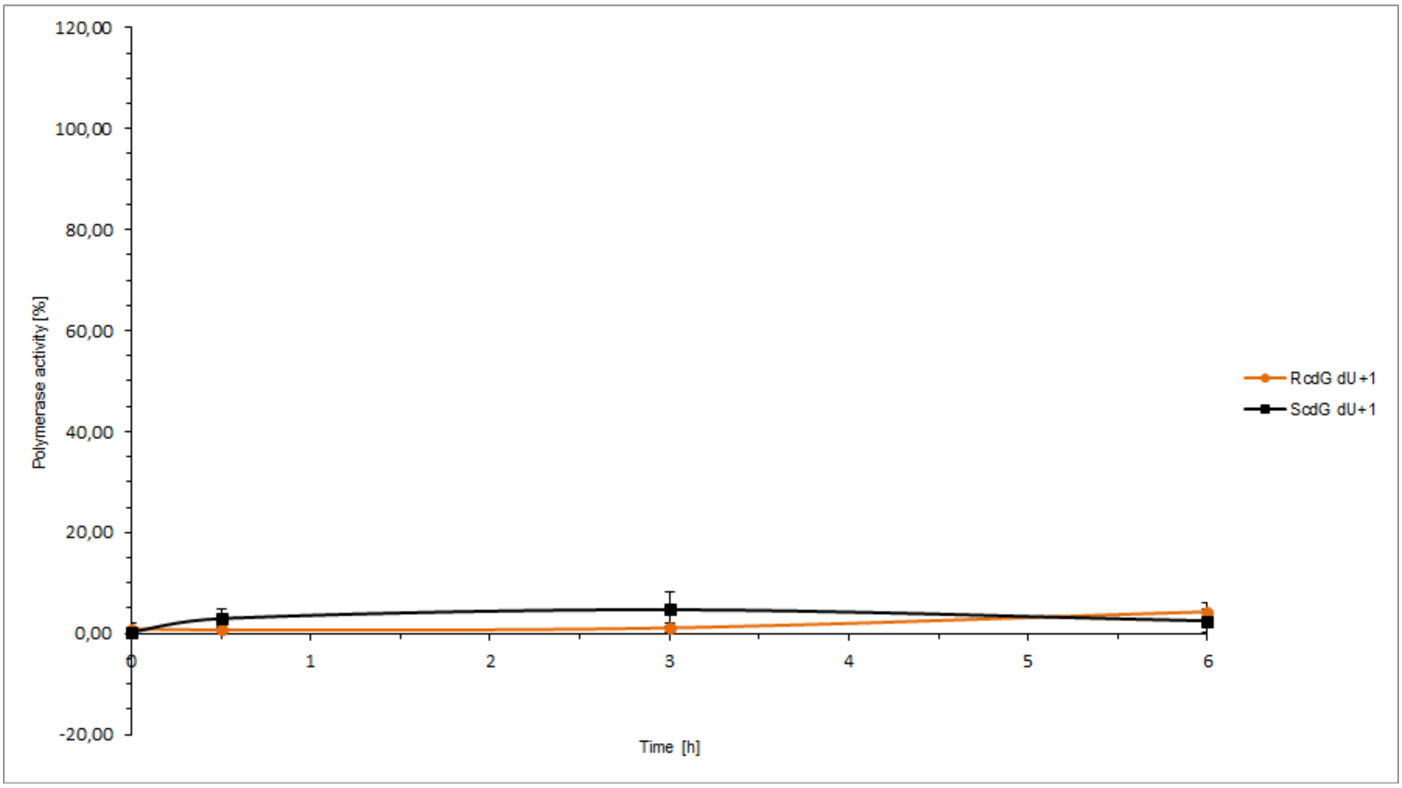
120,00
100,00
80,00
60,00
40,00
20,00
0,00
-20,00
Polymerase activity [%]
0
1
2
3
4
5
6
Time [h]
RcdG dU+1
ScdG dU+1

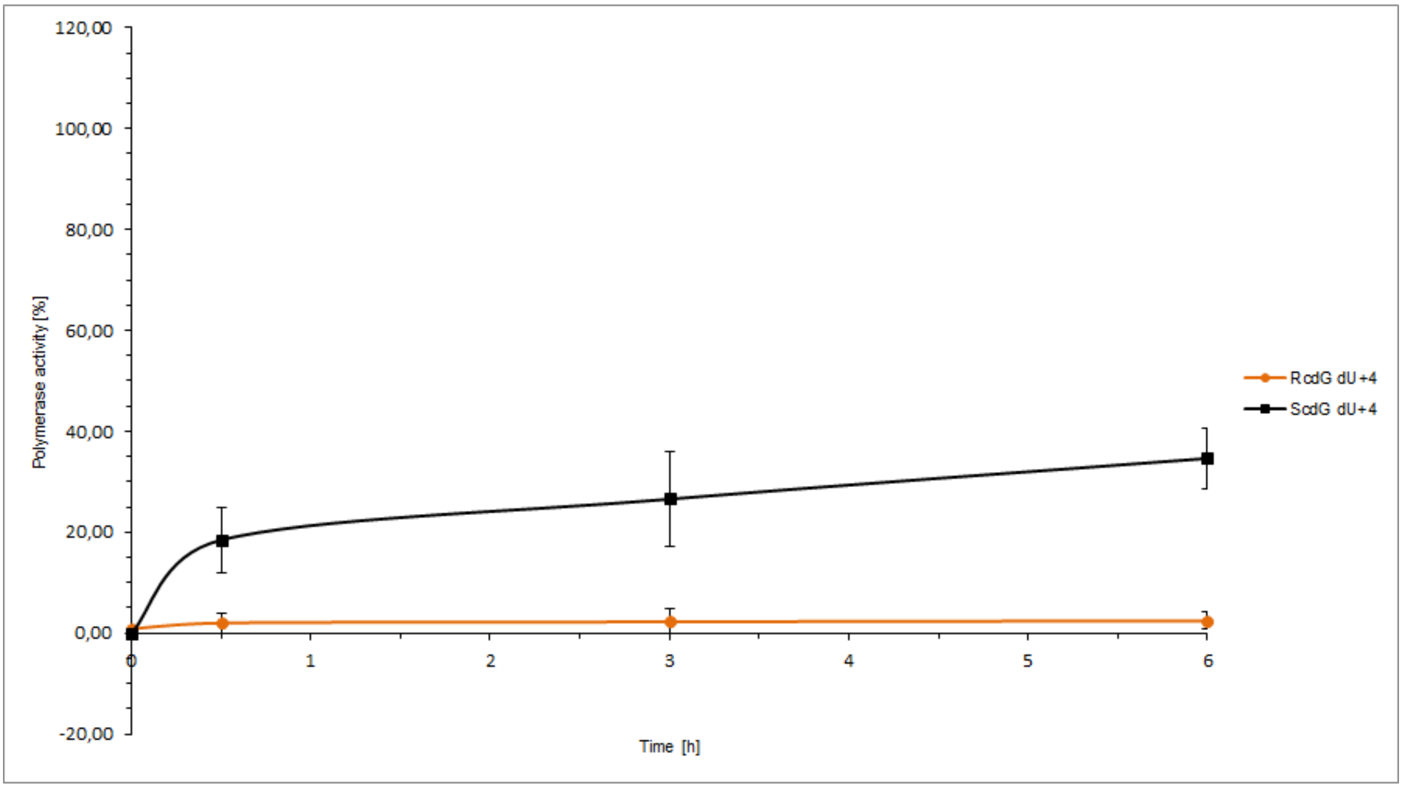
120,00
100,00
80,00
60,00
40,00
20,00
0,00
-20,00
Polymerase activity [%]
0
1
2
3
4
5
6
Time [h]
RcdG dU+4
ScdG dU+4

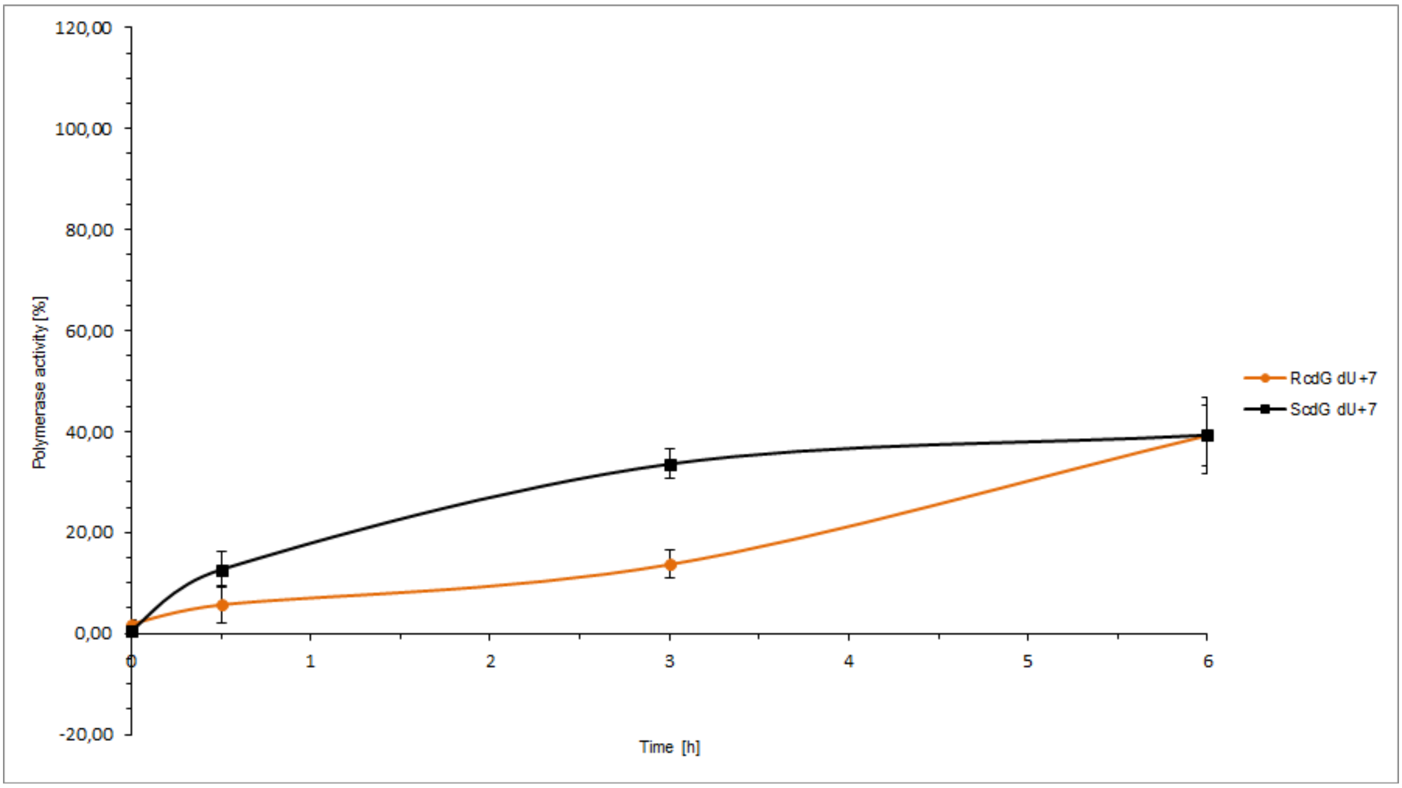
120,00
100,00
80,00
60,00
40,00
20,00
0,00
-20,00
Polymerase activity [%]
0
1
2
3
4
5
6
Time [h]
RcdG dU+7
ScdG dU+7

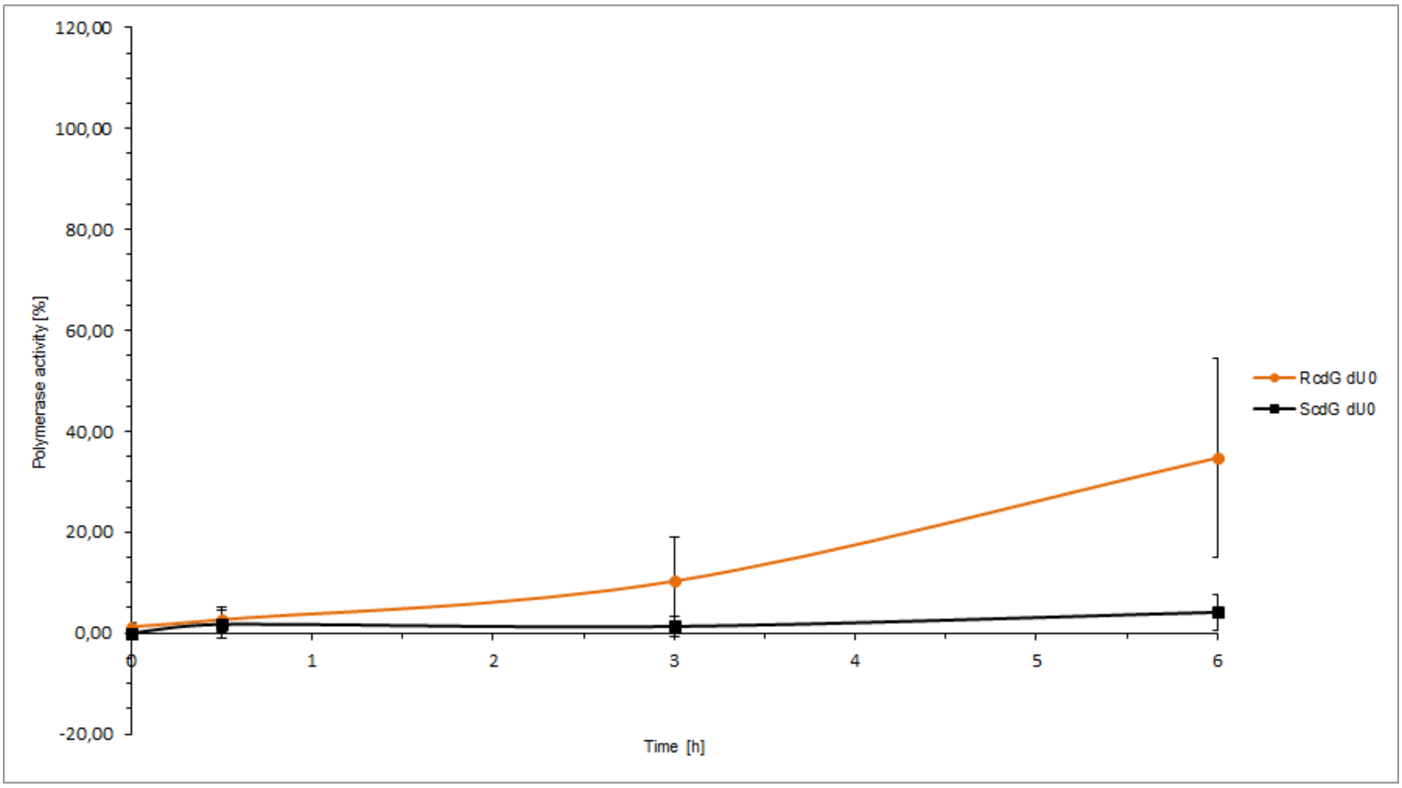

Figure S13. Polymerase activity [%] of ScdG vs. RcdG – comparison of individual strands.

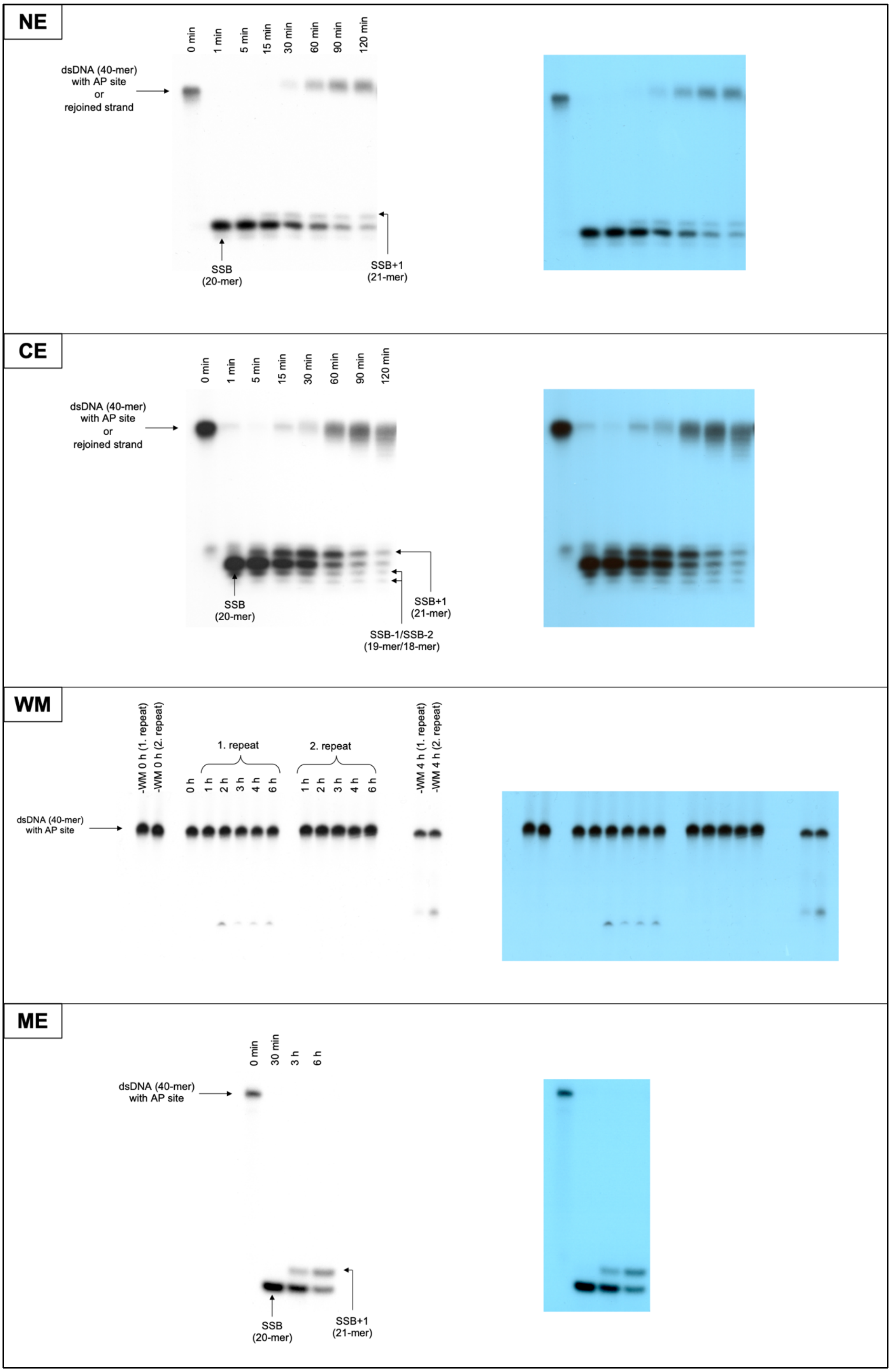

Figure S14. Functional activity test of mitochondrial extract (ME, 20 µg) by comparison with whole mitochondria fraction (WM, 20 µg), nuclear extract (NE, 10 µg), and cytoplasmic extract (CE, 10 µg) of xrs5 cell line using Control 1 (ds-oligo with single AP site lesion). SSB – single strand break (AP site cleavage resulting from endonucleolytic activity of ME, NE, and CE), SSB+1 - cleaved strand with 1 nucleotide incorporated (resulting from polymerase activity of ME, NE, and CE); SSB-1/SSB-2 - cleaved strand with 1 or 2 nucleotides cleaved (resulting from exonucleolytic activity of CE). Original scans of X-ray films are presented on the right, and annotated, graphically processed radiograms are presented on the left.
